# Supplementary material for: Bernoulli’s principle-mediated Cl2 electrosynthesis
Source: Nat Commun. 2026 Jan 24;17:1062. doi: 10.1038/s41467-025-66643-6 (PMC12852893; doi:10.1038/s41467-025-66643-6)
Supplement: Supplementary file 1 — Supplementary Information [file 41467_2025_66643_MOESM1_ESM.pdf]

Supplementary Note 1:

Reagents

Sodium thiosulfate ($Na_2S_2O_3$, 99%, Shanghai Yi'en Chemical Technology Co., Ltd), potassium iodide (KI, 99%, Meryer Technologies Co., Ltd.), potassium dichromate ($K_2Cr_2O_7$, AR, Sinopharm Chemical Reagent Co., Ltd), starch ($(C_6H_{10}O_5)_n$, RG, Shanghai Adamas Reagent Co., Ltd), Ammonium ferrous sulfate ($FeH_8N_2O_8S_2$, 99%, Shanghai Yi'en Chemical Technology Co., Ltd), N,N-Diethyltoluidine ($C_{10}H_{16}N_2$, 99%, Shanghai Adamas Reagent Co., Ltd), 2,6 naphthoic acid ($C_{12}H_8O_4$, 98%, Meryer Technologies Co., Ltd.), N,N-Dimethylformamide (C_3H_7NO, 99.5%, Shanghai Aladdin Biochemical Technology Co., Ltd) Tetraisopropyl Titanate (C_3H_7NO, 99.9%, Shanghai Adamas Reagent Co., Ltd), Sodium hydroxide (NaOH, 99.9%, Shanghai Aladdin Biochemical Technology Co., Ltd), Hydrochloric acid (HCl, AR, Sinopharm Chemical Reagent Co., Ltd), Sulfuric acid (H_2SO_4, AR, Sinopharm Chemical Reagent Co., Ltd), Sodium chloride (NaCl, AR, Sinopharm Chemical Reagent Co., Ltd). All aqueous solutions were prepared with high-purity de-ionized water (DI-water, resistance 18 MΩ. Proton exchange membrane is using Nafion 117.

Supplementary Note 2:

Property calculation

In the literature, there are many studies discussing the *OCl and/or *Cl intermediate formation during CER process. As a typical example, Zhang and co-workers have detected by *in situ* Raman spectrum both *Cl and *OCl intermediates on Ir-based active sites.[1] They have discussed the origin of these intermediates from the perspective of engineering coordination environment (Ir_1O_4 and Ir_1O_6). On another study, the authors have attributed the key of forming *OCl intermediate to appropriate adsorption energy caused by potential differences in electrochemical reaction processes.[2] Further, in

this work, we have experimentally observed *OCl intermediate through *in-situ* Raman experiments (Figure 5a-b). Therefore, we start the DFT calculations from *Cl and *OCl) in the CER process.

The charge difference was computed through the following equation:

$$\rho_{diff} = \rho_{abs} - \rho_{atom} \tag{1}$$

where ρ_{abs} is the electron distribution of the overall system, ρ_{atom} is from corresponding atom systems.

The detailed Gibbs free energy calculation for CER has been carried out as it follows: (G=E + G_{corr}). In G_{corr}, the zero-point energies and entropic contributions were included (G_{corr}=ZPE – TS).[3] The free energy of each intermediate step could be expressed as follows:

$$\Delta G_1 = E(*OCl) - E(*O) - 1/2E(Cl_2) + (\Delta ZPE - T\Delta S)_1 - eU + \Delta G_{ref} \tag{2}$$

$$\Delta G_2 = E(*Cl) - E(*) - 1/2E(Cl_2) + (\Delta ZPE - T\Delta S)_2 - eU + \Delta G_{ref} \tag{3}$$

Where E(*O), E(*OCl), E(*) and E(*Cl) are the total energy of the base material and the adsorbed with Cl, respectively. E(Cl_2) is the energy of the Cl_2 molecule by calculation. The ΔG_{ref} correction was considered as 1.36 eV for chlorine evolution reaction, correlated to the reversible potentials of the chlorine and hydrogen electrodes. eU simulates the value of externally applied potential.

For competitive oxygen evolution, the free energy of Ti–MOF could be presented as follows:

$$\Delta G_3 = E(*OH) - E(*) - E(H_2O) + 1/2E(H_2) + (\Delta ZPE - T\Delta S)_3 - eU \tag{4}$$

$$\Delta G_4 = E(*O) - E(*OH) + 1/2E(H_2) + (\Delta ZPE - T\Delta S)_4 - eU \tag{5}$$

$$\Delta G_5 = E(*OOH) - E(*O) - E(H_2O) + 1/2E(H_2) + (\Delta ZPE - T\Delta S)_5 - eU \tag{6}$$

$$\Delta G_6 = E(*) - E(*OOH) - E(O_2) + 1/2E(H_2) + (\Delta ZPE - T\Delta S)_6 - eU \tag{7}$$

Where E(*), E(*OH), E(*O) and E(*OOH) are the total energy of the base material and the adsorbed with OH, O and OOH, respectively. $E(H_2)$, $E(O_2)$ and $E(H_2O)$ is the energy of the H_2, O_2, H_2O molecule by calculation. eU simulates the value of externally applied potentials.

The Pourbaix diagram of Ti-MOF was constructed by plotting the surface changes under the corresponding U_{SHE} and pH. The Ti-MOF substrate and two intermediate species, *OH and *O, were calculated by examining the formation of materials and intermediates.[4] The following is the quantitative relationship between △G, pH and U:

$$\Delta G_{*OH}\ (pH, U_{SHE})=\Delta E(*OH) + \Delta ZPE - T\Delta S - 0.059pH - eU_{SHE} \quad (8)$$

$$=\Delta G_1 - 0.059pH - eU_{SHE}$$

$$\Delta G_{*O}\ (pH, U_{SHE}=\Delta E(*O) + \Delta ZPE - T\Delta S - 2 \times 0.059pH - 2eU_{SHE} \quad (9)$$

$$=\Delta G_2 - 0.118pH - 2eU_{SHE}$$

where the algorithm for ΔG is the same as the algorithm for Gibbs free energy.

Supplementary Note 3:

Technic-economic analyses

The accuracy of simulation models was evaluated by comparison with calculations conducted according to Faraday's Law. For Ti-MOF, it can facilitate the electrochemical reduction of NaCl to Cl_2 with FE of 70% at the current density is 1.14 A cm^{-2}.

The total current is:

$$I = I_S \times s_{rea} = 1.14\frac{A}{cm^2} \times 500\ m^2 \times \frac{10000\ cm^2}{m^2} = 5700000\ A$$

The power needed is given as:

$$power = UI = 2.4\,V \times 5700000\,A = 13680000\,W = 13680\,kW$$

The yield rate of NaOH and Cl_2:

$$\frac{m_{Cl_2}}{hr} = \frac{I \times M \times t \times FE}{n \times F \times 1\,hr} = \frac{5700000\,A \times 71\frac{g}{mol} \times 3600\,s \times 70\%}{2 \times 96485\frac{C}{mol} \times 1\,hr} = 5284.99\,kg/hr$$

$$\frac{m_{NaOH}}{hr} = \frac{I \times M \times t \times FE}{n \times F \times 1\,hr} = \frac{5700000\,A \times 40\frac{g}{mol} \times 3600\,s \times 70\%}{1 \times 96485\frac{C}{mol} \times 1\,hr} = 5954.92\,kg/hr$$

In the process of generating NaOH and Cl_2, NaCl is constantly consumed, so the mass of NaCl consumed per hour in the whole process is:

$$\frac{m_{NaCl}}{hr} = \frac{I \times M \times t \times FE}{n \times F \times 1\,hr} = \frac{5700000\,A \times 58.44\frac{g}{mol} \times 3600\,s \times 70\%}{2 \times 96485\frac{C}{mol} \times 1\,hr} = 8709.06\,kg/hr$$

The H_2O consumption for the anodic HER reaction is:

$$cathode(H_2O) = \frac{I \times M \times t \times 2}{n \times F} = \frac{5700000\,A \times 18\,g/mol \times 3600\,s \times 2}{4 \times 96485\,g/mol \times 1\,hr} = 1914.08\,kg/hr$$

The H_2 for the anodic HER reaction is:

$$cathode(H_2) = \frac{I \times M \times t}{n \times F \times 1\,hr} = \frac{5700000\ \ A \times 2\,g/mol \times 3600\,s}{2 \times 96485\,g/mol \times 1\,hr} = 212.68\,kg/hr$$

The above calculations consist well with our simulation results (please see Supplement Table 8), thus confirming the accuracy of the simulation models.

Further, the technical and economic analyses of DSA-based system with membranes have also been conducted based on the electrochemical data from literature (1 A cm^{-2}, Faradaic efficiency of 99.5%).[5] The specific calculation steps have been placed in Supplementary Tables 7 and 11.

The calculation of capital input cost

According to aspen's estimation, the electrolyzer, evaporation device, etc. of the whole process

and their installation costs as follows:

$$Electrolyzer\ cost = \$1120350$$

$$Evaporating\ plant\ cost = \$2738300$$

$$Installation\ cost = \$2738300$$

We depreciate the cost of fixed capital to each year with a period of 20 years of project operation, and the interest rate is 3.25%.

$$Depreciation\ of\ fixed\ capital = \frac{(\$1120350 + \$2738300 + \$4071700) \times 3.25\%}{1 - \frac{1}{(1 + 3.25\%)^{20}}}$$

$$= \$545440.62/year$$

The calculation of operating costs

There are 8000 hrs of operation every year, and the rest is used for equipment maintenance. Consequently, we simulate the energy consumption of various operations including heat exchanger, pump and compressor. Specifically,

Regarding electricity price, we consider the discrepancy in electricity pricing data stemming from different consuming sectors. Particularly, U.S. electricity rates exhibit significant variations across residential, commercial, and industrial sectors. Given that significant role of electricity price for production cost estimates, we have retrieved a number of state-of-the-art literatures,[6,7,8,9] and select the electricity price as 0.03 \$ kWh^{-1}.[10,11] The electricity cost is calculated according to the electricity demand and price:

$$Electrolysis\ electricity\ cost = 13680\ kW \times \frac{\$0.03}{kWh} \times 8000\frac{h}{year} = \$3283200/year$$

$$Separation\ electricity\ cost = 20934\ kW \times \frac{\$0.03}{kWh} \times 8000 \frac{h}{year} = \$5024160/year$$

The maintenance cost is assumed 2.5% of capital cost per year (from H_2A):

$$Maintenance\ cost = \frac{\$545440.62}{year} \times 2.5\% = \$13636/year$$

The price of NaCl is \$0.0928/kg, and the price required for raw materials is as follows:

$$NaCl\ cost = \frac{8709.06\ \frac{kg}{hr} \times\ 8000\ hr}{year} \times \$0.0928/\text{kg} = \$6465608.81/year$$

The price of water is \$0.409/ton, and the price required for water is as follows:

$$Water\ cost = \frac{1914.08 \frac{kg}{hr} \times\ 8000\ hr}{year \times 1000\ kg} \times \$0.409/\text{ton} = \$6262.87/year$$

In addition, if proton membrane is needed, according to the membrane price of \$700/m^2, the operation cost of each film replacement accounts for 30% of the equipment cost. The frequency of film replacement is once every two years, and each replacement and commissioning takes about 30 days (657 hrs). Therefore, the total cost of using the membrane is as follows:

$$Membrane\ cost$$

$$= \frac{\frac{\$700}{m^2} \times 500\ m^2}{20year} \times 10\ times + \frac{\$559076}{2\text{year}} \times 30\%$$

$$+ \left(\frac{5223.61kg}{hr} \times \frac{\$0.45}{kg} + \frac{4635.95kg}{hr} \times \frac{\$0.054}{kg} + \frac{186.56kg}{hr} \times \frac{\$4.77}{kg}\right.$$

$$\left. - \frac{7639.53kg}{hr} \times \frac{\$0.0928}{kg} - \frac{1679.017kg}{hr} \times \frac{\$0.000409}{kg}\right) \times \frac{657hr}{2year}$$

$$= \frac{\$175000}{year} + \frac{\$227843.295}{year} + \frac{\$913631.39}{year} = \$1172492.8/year$$

According to the average wage of the American manufacturing industry, it is estimated that the labor cost in the first year will be \$500000, and the annual wage will increase by 5%. Therefore, the total labor cost for 20 years is \$16532977.05.

The cost price of product (NaOH+Cl_2) (without membrane) is:

$$\begin{aligned}
&\mathit{The\ cost\ price\ of}\ \text{product}\\
&\quad= \text{Raw material cost} + \text{Separation cost} + \text{Equipment cost} + \text{Electrolysis cost}\\
&\quad= [\left(\frac{\$597050}{year} + \frac{\$3283200}{year} + \frac{\$6465608.8}{year} + \frac{\$6262.9}{year} + \frac{\$13636}{year} + \frac{\$5024160}{year}\right)\\
&\quad\times 20\ year + \$16532977.05]/(\frac{5223.61 + 52584.99\ kg}{h} / 2 \times \frac{8000h}{year} \times 20\ year)\\
&\quad= \$0.36\ kg^{-1}
\end{aligned}$$

The cost price of product (NaOH+Cl_2) (with membrane) is:

$$\begin{aligned}
&\mathit{The\ cost\ price\ of}\ \text{product}\\
&\quad= \text{Raw material cost} + \text{Separation cost} + \text{Equipment cost} + \text{Electrolysis cost}\\
&\quad+ \text{membrane cost}\\
&\quad= \left[\left(\frac{\$597050}{year} + \frac{\$3283200}{year} + \frac{\$6465608.8}{year} + \frac{\$6262.9}{year} + \frac{\$13636}{year} + \frac{\$5024160}{year}\right.\right.\\
&\quad\left.\left. + \frac{\$1172492.8}{\text{year}}\right) \times 20\ year\right] / (\frac{5223.61 + 52584.99\ kg}{h} / 2 \times \frac{8000h}{year} \times 20\ year)\\
&\quad= \$0.39\ kg^{-1}
\end{aligned}$$

The is given by the product revenue minus operating costs, and 25% tax.

In addition, The FNPV is analyzed, indicating that the scheme is feasible and the investment benefit is good (Supplementary Tables 8–10).

$$FNPV = \sum_{t=0}^{n} (Cl - CO)_t \times (1 + i)^{-t} \tag{10}$$

Where *Cl* is the present value of future cash flow, *CO* is the present value of the original investment, *i* is the discount rate, and *t* is the duration.

Supplementary Figures and tables

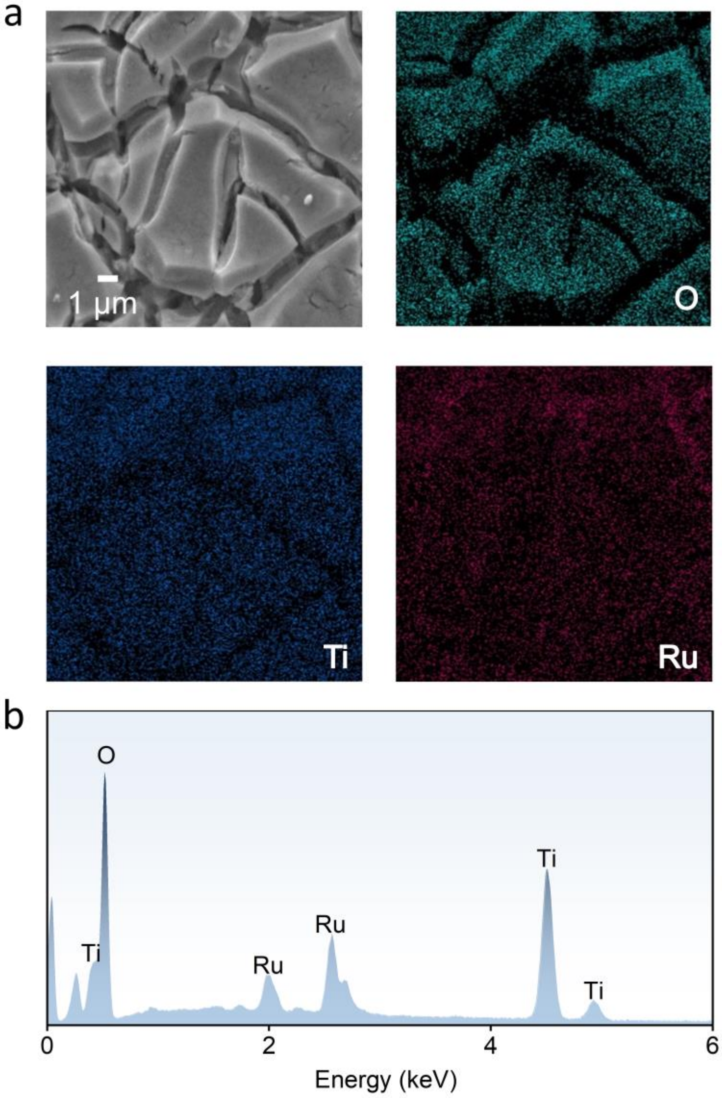

Supplementary Figure 1. Morphology characterizations of commercial DSA. a, SEM and EDS mappings. **b**, Element percentages calculated from EDS.

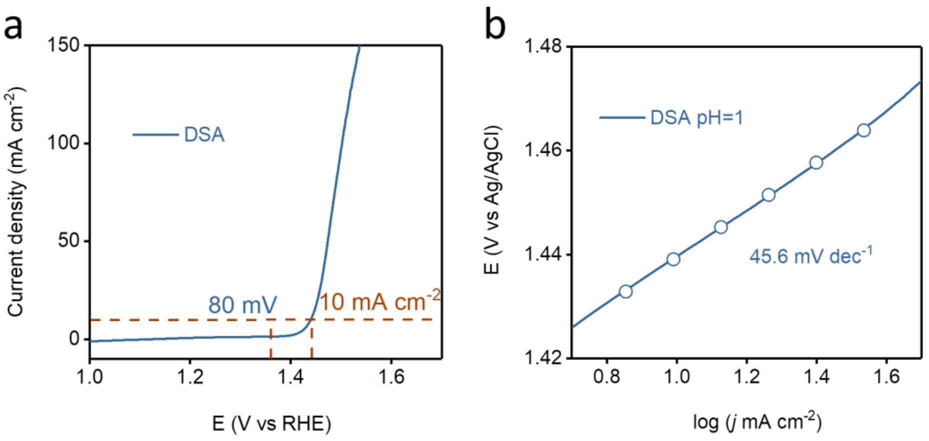

Supplementary Figure 2. Electrochemical data of DSA for CER. a, Linear sweep voltametry curves (LSV). **b**, The corresponding Tafel slope from LSV.

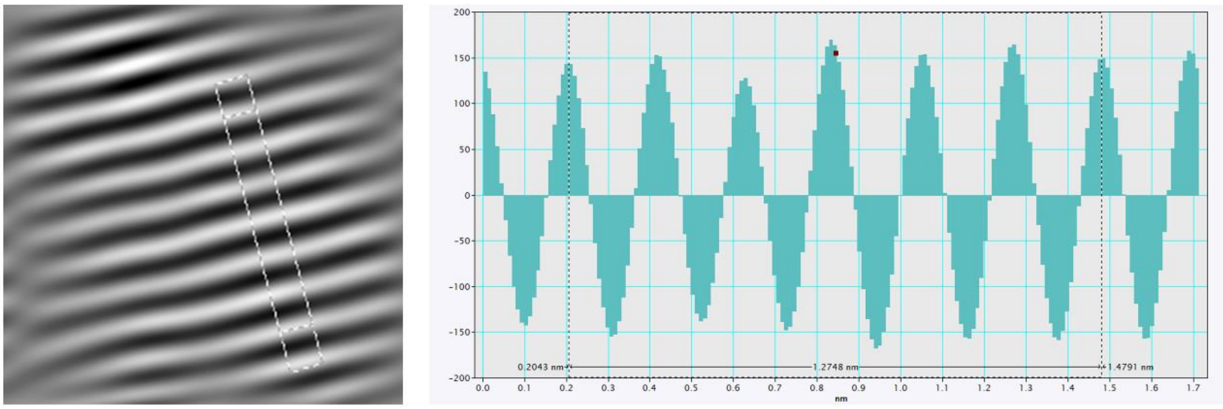

Supplementary Figure 3. The dislocation analyses for measuring crystal plane spacing of Ti-MOF.

In the main text, Figure 2a (inset) demonstrates clear lattice fringe with interplanar spacing of 0.21 nm. This result is consistent to Supplementary Figure 3, which validates the crystallinity through Fast Fourier Transform (FFT)-processed dislocation analyses and quantified interplanar spacing measurements. More specifically, the lattice region within red box in Figure 2a (main text) was FFT-processed and measured across six consecutive intervals (total distance: 1.2748 nm), yielding a mean spacing of 0.21 nm.

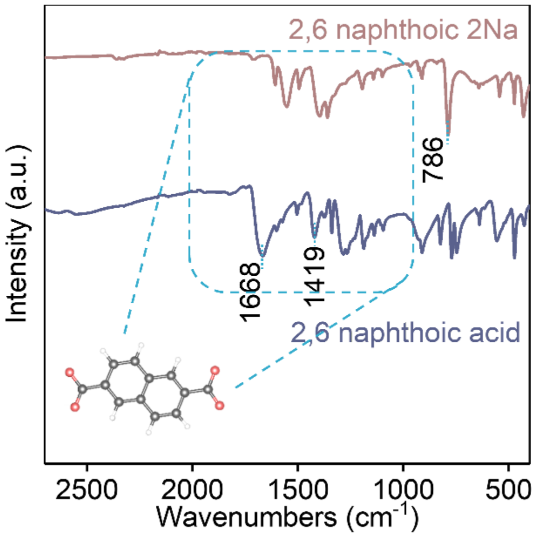

Supplementary Figure 4. Fourier Transform infrared spectroscopy (FTIR) of 2,6 naphthoic acid and 2,6 naphthoic 2Na.

The characteristic vibrational peaks of H_2NDC at 1668 and 1415 cm^{-1} remain present in Ti-MOF. Notably, no detectable peaks appear in the fingerprint region (500-1000 cm^{-1}) resembling the intense bands in Figure 2c of the main text (attributed to Ti-O vibrations).[12] The FTIR spectrum of disodium 2,6 naphthoic 2Na further exhibits a sharp, intense peak at 786 cm^{-1}, providing spectroscopic evidence for sodium-mediated deprotonation *via* hydrogen atom transfer.[13] Collectively, these results confirm effective coordination between Ti centers and carboxylate groups in the synthesized MOF.

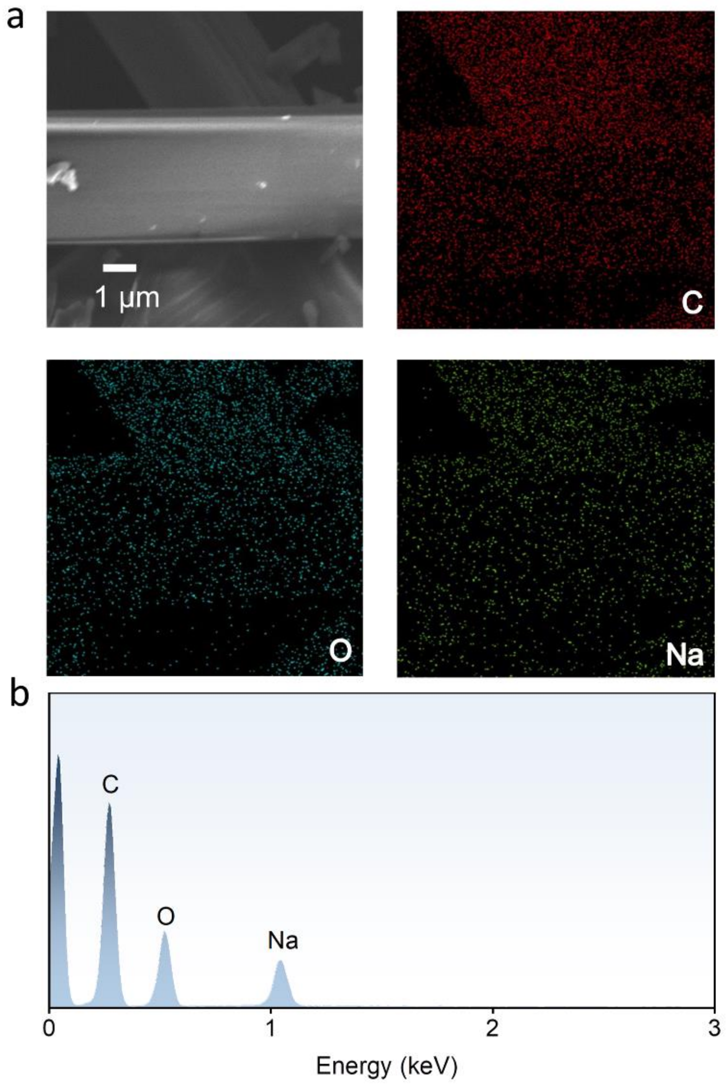

Supplementary Figure 5. Morphology characterization of 2,6 naphthalene-2Na precursor synthesized by mixing 2,6 naphthoic acid and sodium hydroxide. a, SEM and EDS mappings. **b,** Element percentages from EDS.

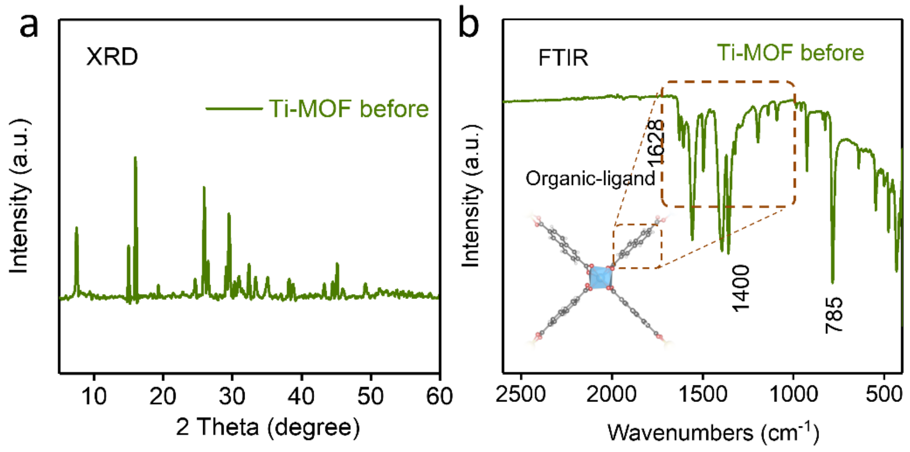

Supplementary Figure 6. Structural characterization of Ti-MOF before calcination. a, X-Ray diffraction (XRD) and **b,** Fourier Transform infrared spectroscopy (FTIR) of Ti-MOF precursor.

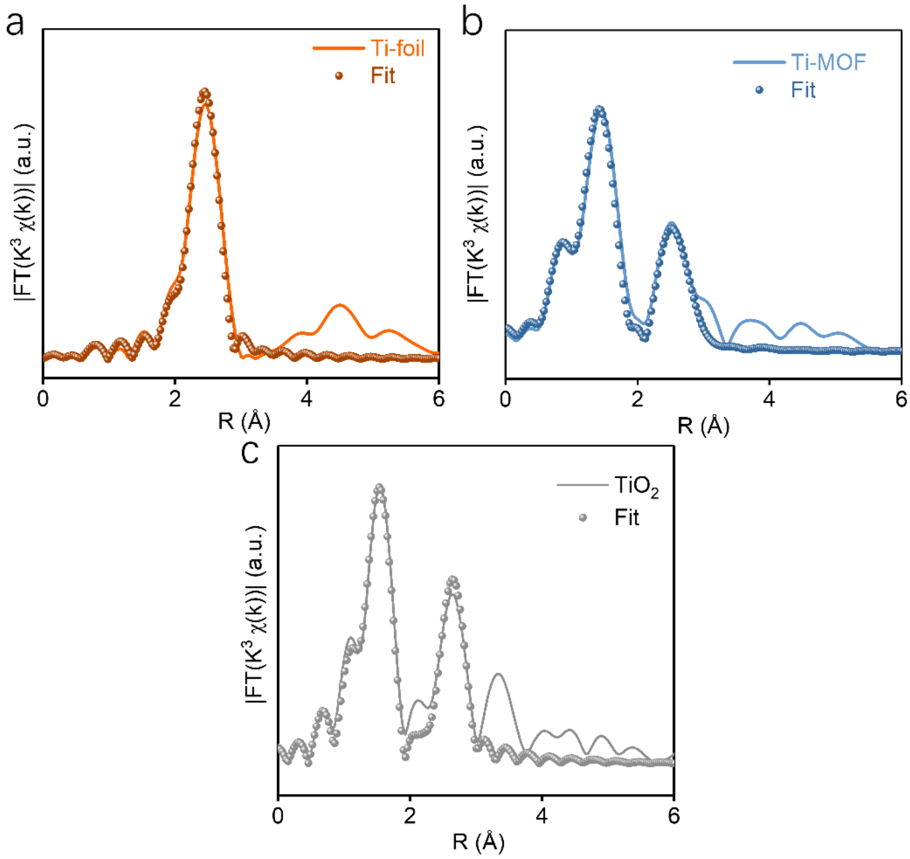

Supplementary Figure 7. Extended X-ray absorption fine structure (EXAFS) and data fitting.

a, Ti-foil. **b,** Ti-MOF. c, TiO_2.

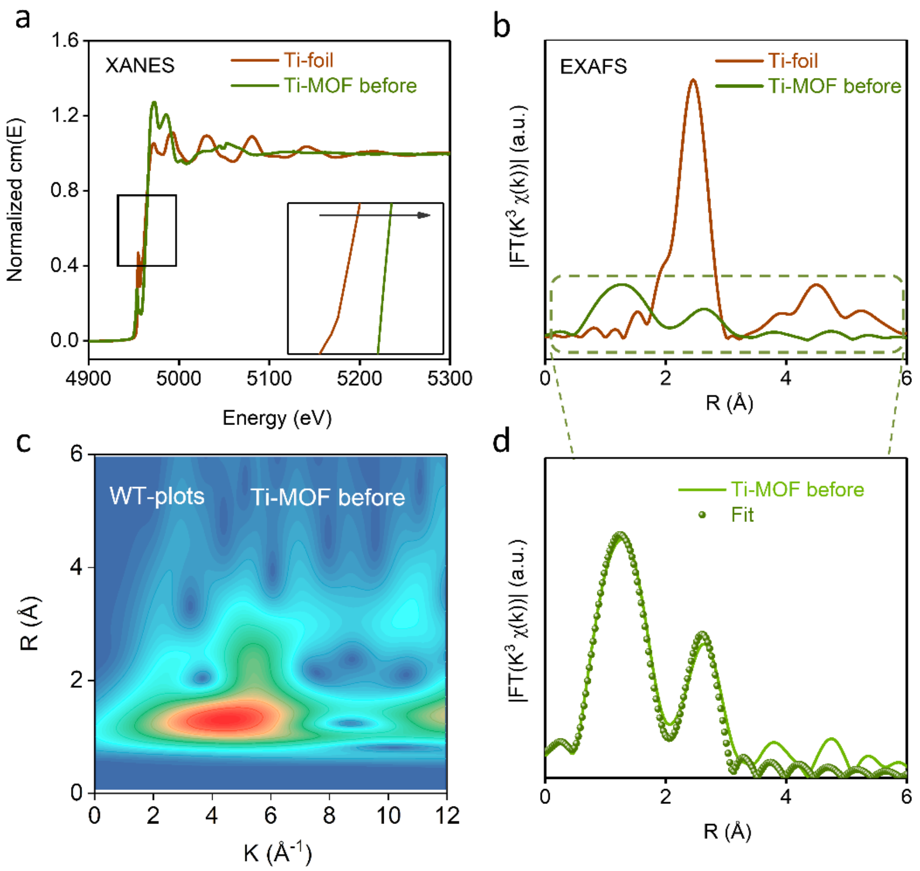

Supplementary Figure 8. Synchrotron characterizations of Ti-MOF before calcination. a, X-ray absorption near edge structure (XANES). **b, d,** Extended X-ray absorption fine structure (EXAFS). **c,** Wavelet transform plots (WT-plots).

WT analysis can serve as a key method to resolve ambiguous structures. In the present study, it can be used to distinguished the overlapping contributions of Ti-O shell (1.8–2.0 Å)[14] and Ti-Ti shell (2.8–3.5 Å):[15] *i*) the WT intensity maximizes at k = 4–6 $Å^{-1}$ (Ti-O) and k = 6–8 $Å^{-1}$ (Ti-Ti) providing independent validation of the atomic pair assignments for fitting models; *ii*) WT analysis maps the EXAFS signals in the k-space (energy) and R-space (distance), thus providing a two-dimensional representation. This can clearly separate these overlapping shells based on their scattering amplitudes and phase differences. The high spatial resolution is very helpful in confirming the coexistence of multiple coordination environments, which is crucial for understanding the structural evolution of the

catalyst under reaction conditions.

Secondly, WT analysis can serve as a powerful qualitative tool for probing coordination environments in the present study (Supplementary table 2). When processing and analyzing images, WT better mimics the human visual system's perception mechanisms, aligning closely with human visual cognition patterns. Therefore, it enables more effective capture and processing of fine details and edge information within images, resulting in processed visuals that appear more natural and sharper. This enhancement facilitates researchers' accurate extraction of information from images, thereby improving the precision and reliability of image-based analysis.

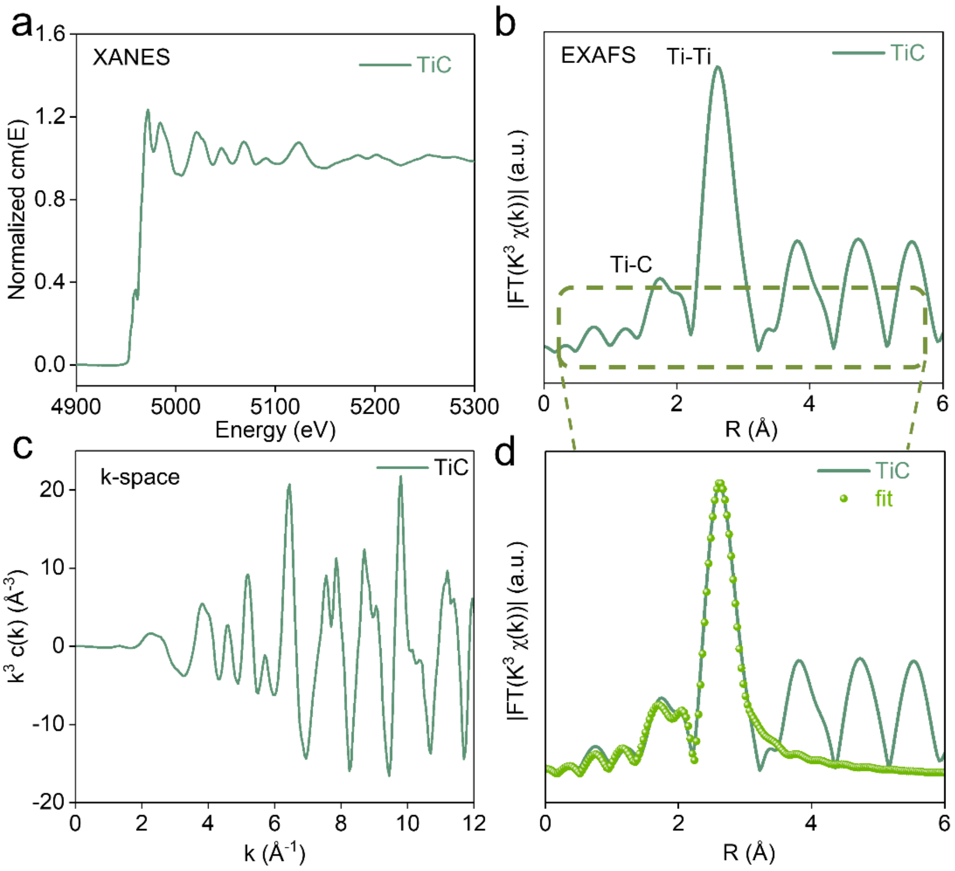

Supplementary Figure 9. Synchrotron radiation of TiC. a, XANES spectra of reference TiC. **b, d** EXAFS spectra of reference TiC and data fitting. **c,** the k-space XAFS data for edges of reference TiC.

The additional TiC reference data, as presented in Supplementary Figure 9 and Supplementary Table 2, reveal a Ti-C bond distance of 2.14 Å. This length is significantly longer than Ti-O bonds (1.92 Å), thereby precluding the inclusion of Ti-C configurations within the structural framework of our material system.[16]

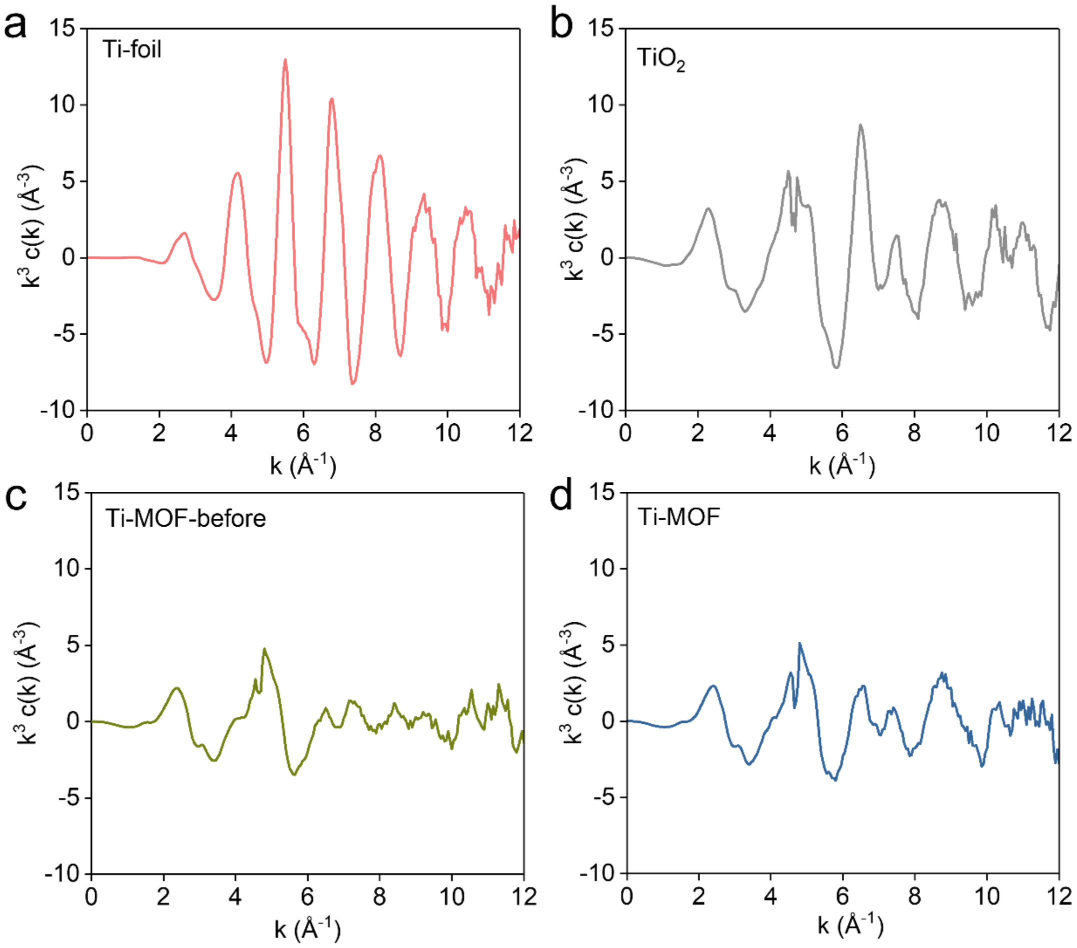

Supplementary Figure 10. The k-space XAFS data for edges for different samples. **a,** Ti-foil. **b,** TiO_2. **c,** Ti-MOF before calcination. **d,** Ti-MOF.

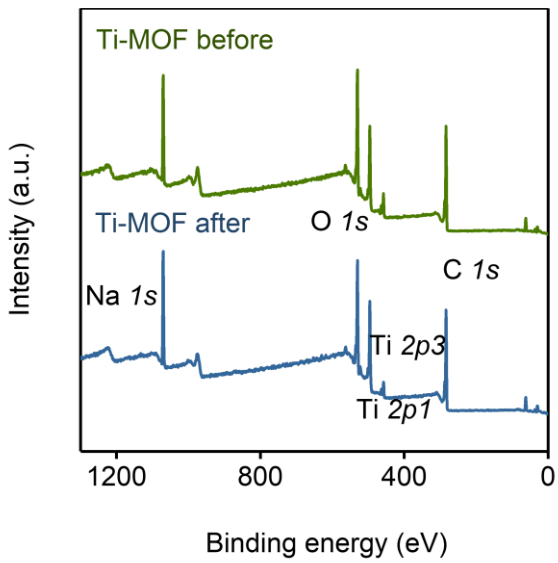

Supplementary Figure 11. XPS overall survey of Ti-MOF before and after calcination at 450 °C for 3 hrs at a heating rate of 5 °C min^{-1} under argon atmosphere.

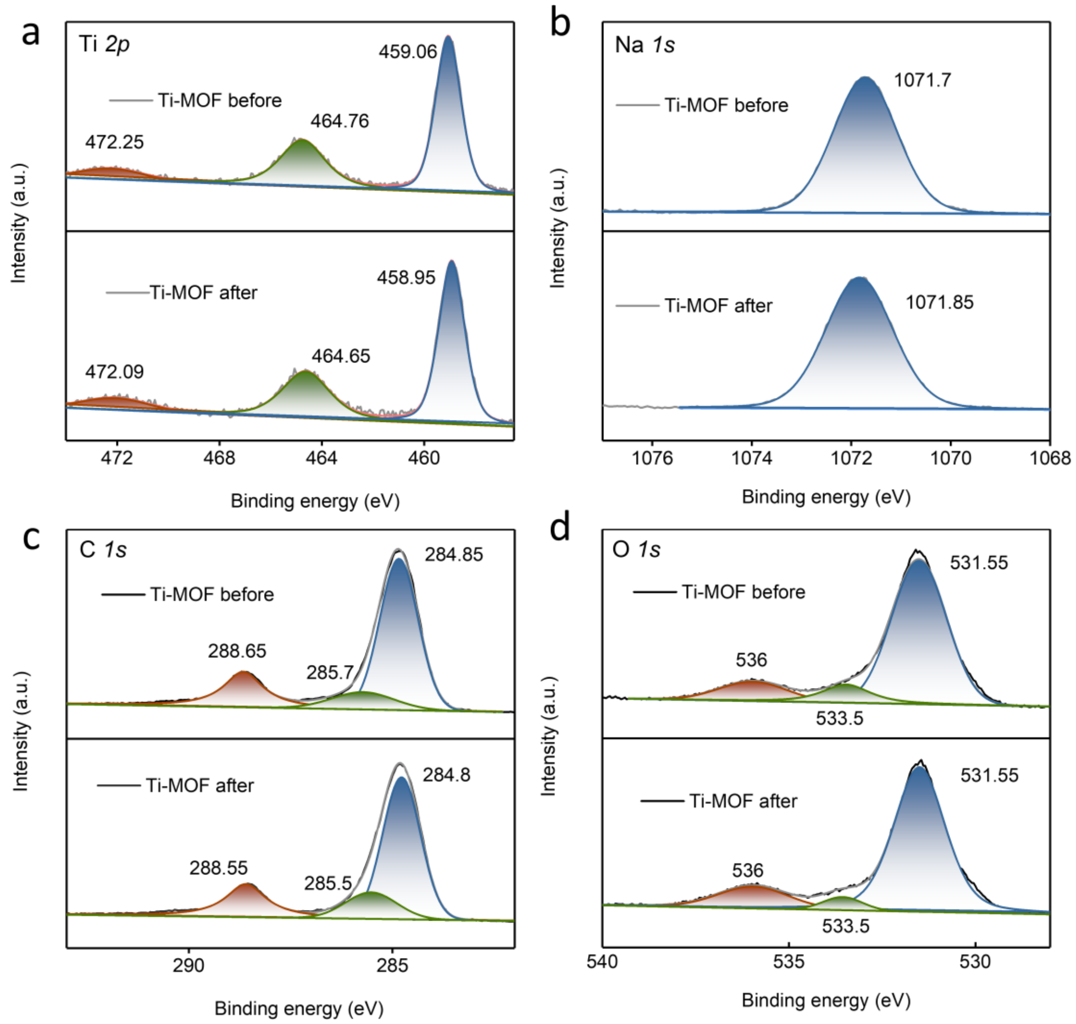

Supplementary Figure 12. XPS spectrum of Ti-MOF before and after calcination at 450 °C for 3 hrs at a heating rate of 5 °C min^{-1} under argon atmosphere. a, Ti 2p, **b,** Na 1s, **c,** C 1s, **d,** O 1s.

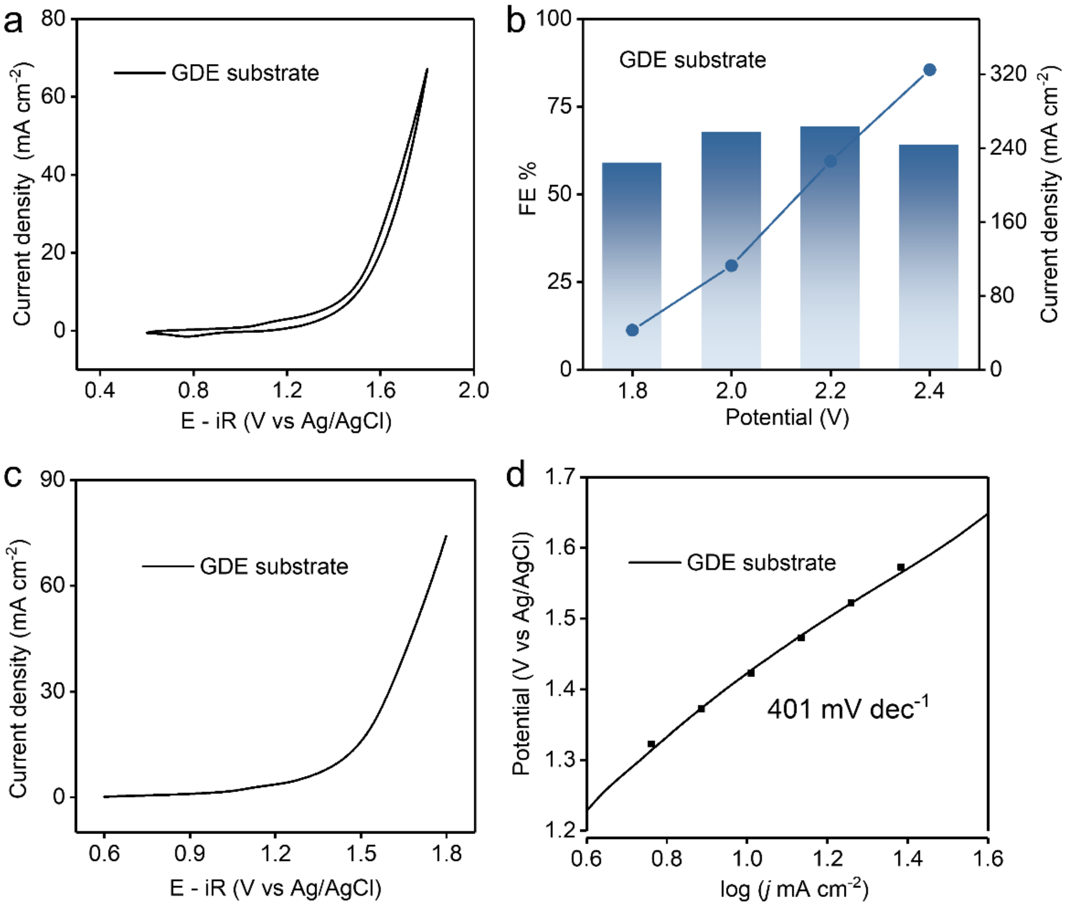

Supplementary Figure 13. Electrochemical tests of GDE substrate in 5 M NaCl at pH=7. a, CV. **b,** Cl_2 Faradic efficiencies and current densities from 1.8 to 2.4 V (*vs.* Ag/AgCl). **c,** LSV. **d,** Tafel slope from LSV.

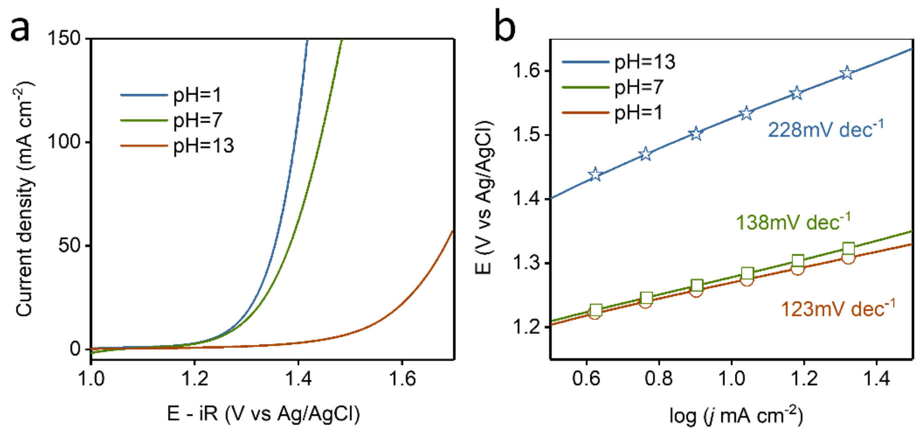

Supplementary Figure 14. Electrochemical data of Ti-MOF in 5 M NaCl at different pHs and test compensation for 90%. a, LSVs. **b,** Tafel slopes from LSVs.

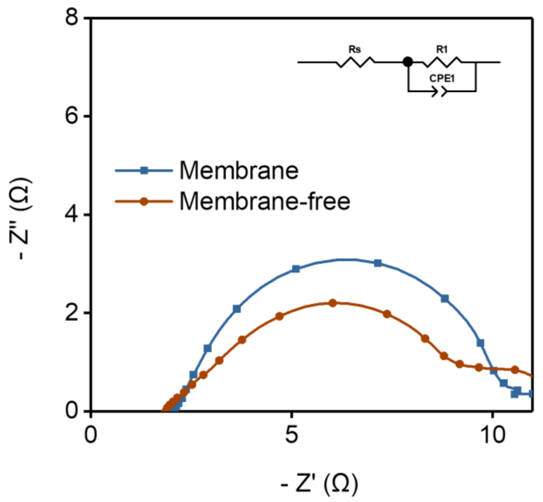

Supplementary Figure 15. Electrochemical impedance spectroscopy (EIS) measurements for membrane-based and -free electrochemical cells.

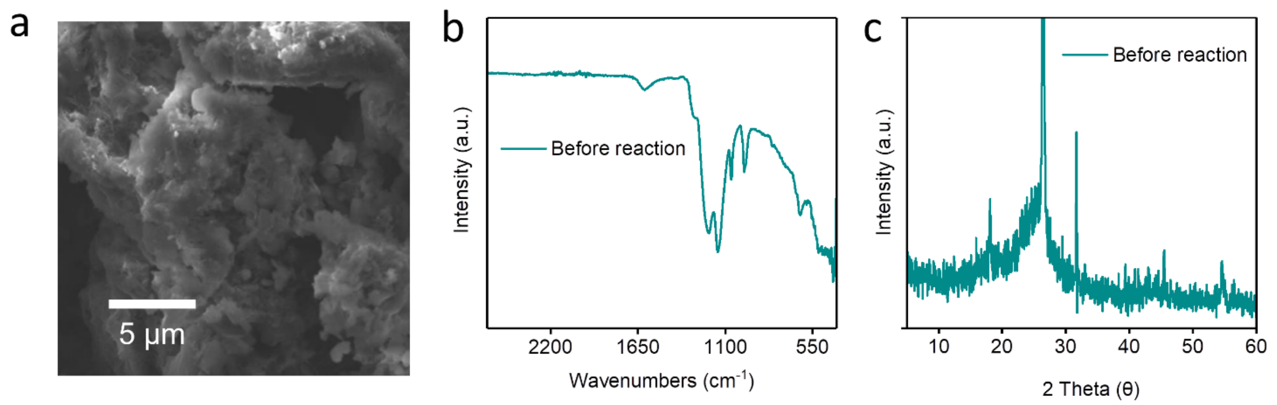

Supplementary Figure 16. The characterizations of Ti-MOF on gas diffusion electrode (GDE) before chlorine evolution reaction (CER). a, SEM. **b,** FTIR. **c,** XRD.

To explore the changes of Ti-MOF before and after the reaction, we conducted a series of studies on it before and after the reaction:

1) Firstly, the Ti-MOF was characterized by XRD (Supplementary Figure 16c). After loading Ti-MOF on GDE (gas-diffusion electrode) substrate, there is a large carbon peak around 26°. However, many crystal plane peaks belonging to Ti-MOF still exist (15.9°, 18.1°, 31.7 ° and 45.5°). Even after extreme acidic/base conditions and long-term stability tests (Supplementary Figures 20c, 23c and 49a), these crystal plane peaks still exist and show little change.

2) Secondly, we have conducted Fourier transform infrared spectroscopy analysis (Supplementary Figure 16b). Many vibration signals exist after Ti-MOF loaded on GDE substrate: Ti-O (629 cm^{-1}), organic-ligant (C=C 1604 cm^{-1}, C-H 979, 1061 cm^{-1}), and C-O (1147, 1207 cm^{-1}).[17] These peaks show insignificant change after extreme acidic/base condition or long-term stability tests (Supplementary Figures 20b, 23b and 49b).

3) Further, XPS was conducted for both before and after electrochemical tests (Supplementary Figure 19). The Ti 2p of Ti-MOF was detected before and after reaction. In the O 1s spectrum, Ti-O (lattice oxygen), C=O and C-O were detected. Compared with the characteristic peaks before test, the binding energies of all subsequent peaks changed insignificantly. Moreover, in C 1s spectrum, C=C, C-C, C-O and π-π* peaks were detected. The positions of these peaks rarely changed, which indicates Ti-MOF maintains good reaction stability.

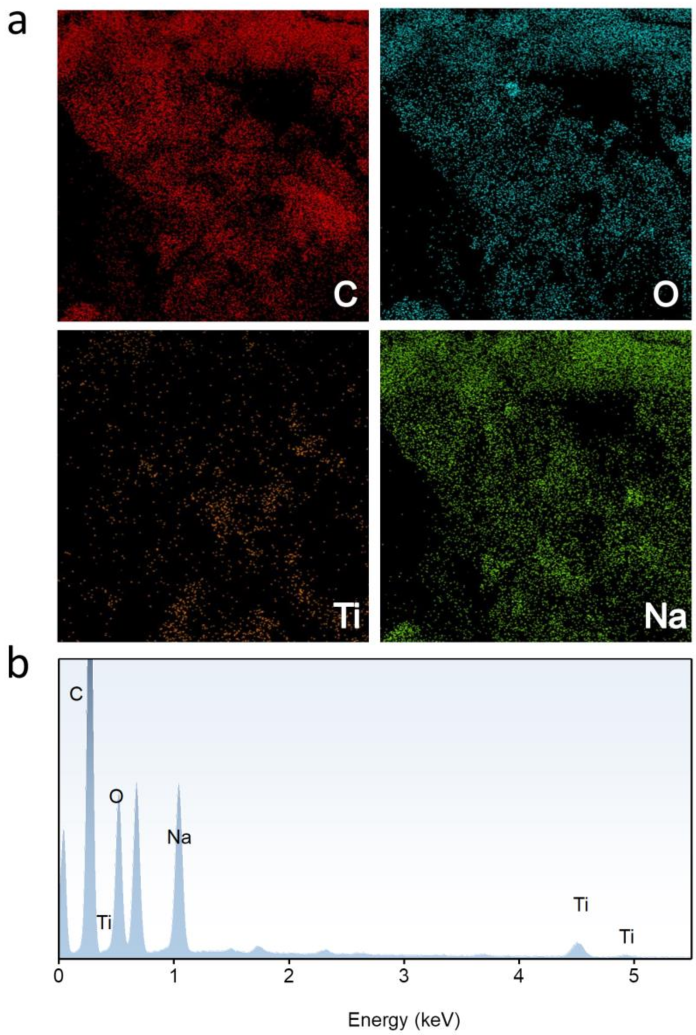

Supplementary Figure 17. Morphology characterization of Ti-MOF on GDE before CER. a, EDS mappings. **b,** Element percentages from EDS.

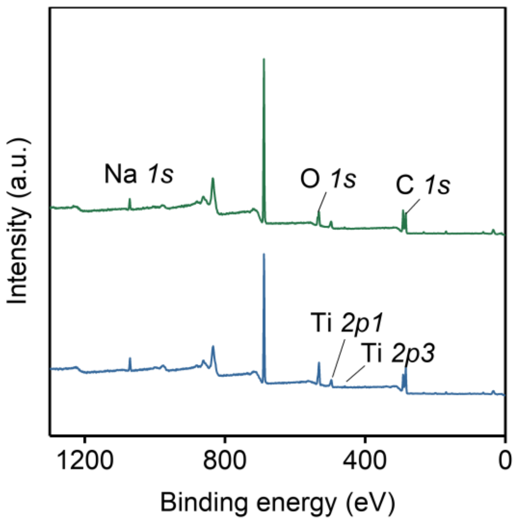

Supplementary Figure 18. XPS overall survey of Ti-MOF on GDE before and after CER in 5 M NaCl at pH=13

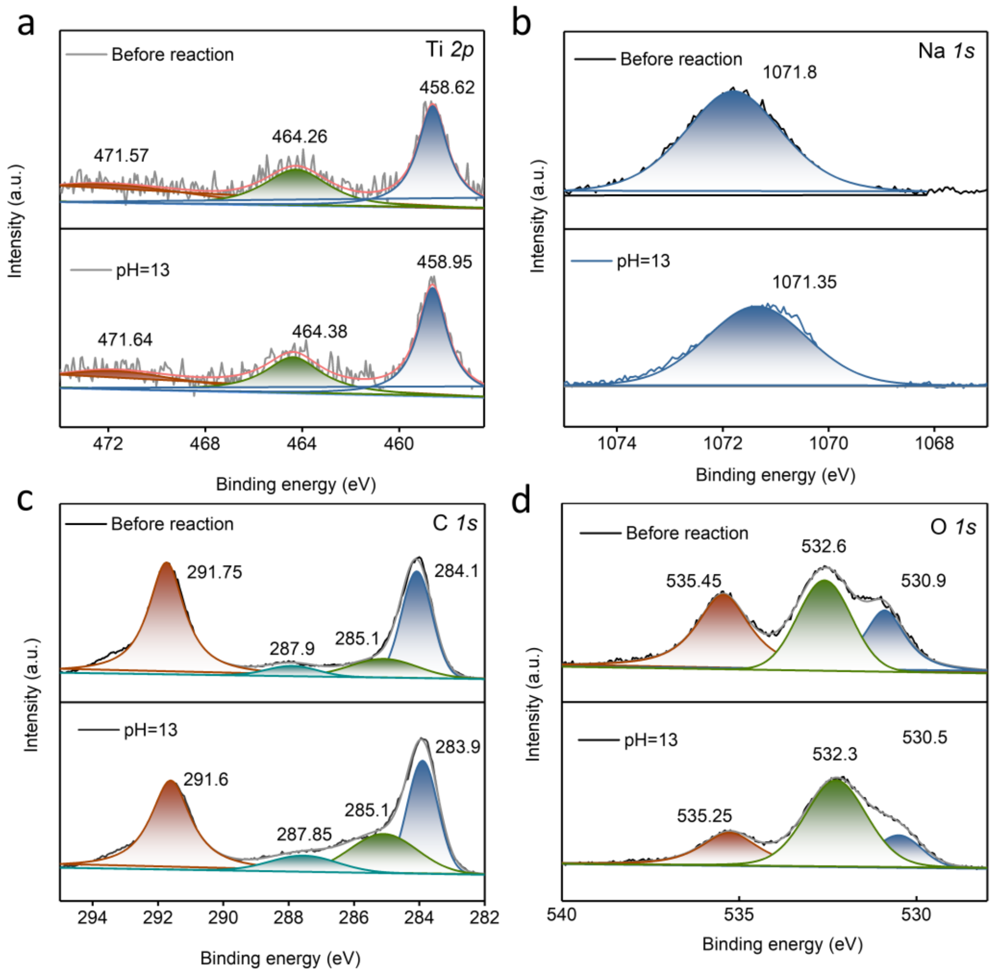

Supplementary Figure 19. XPS spectra of Ti-MOF on GDE before and after CER in 5 M NaCl at pH=13. a, Ti 2p, **b,** Na 1s, **c,** C 1s, **d,** O 1s.

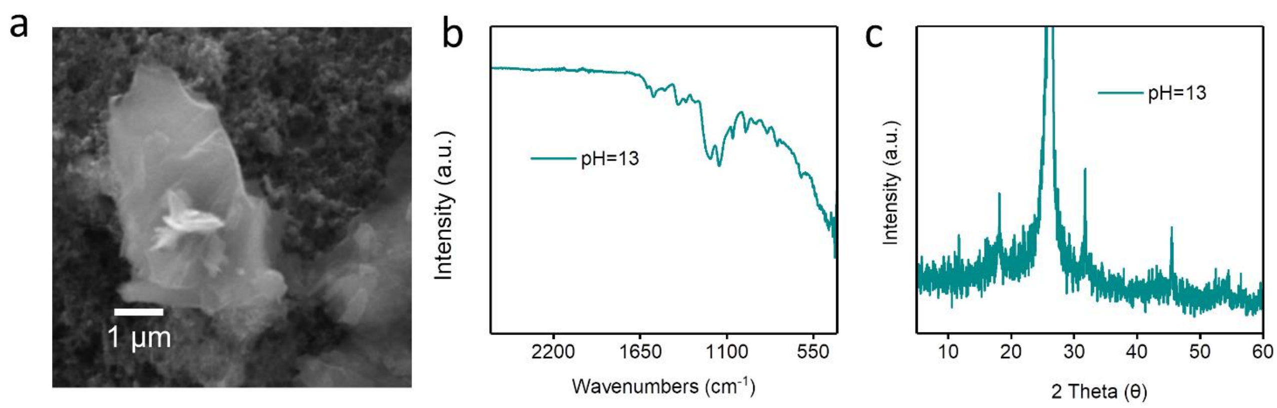

Supplementary Figure 20. Characterization of Ti-MOF on GDE after CER in 5 M NaCl at pH=13. a, SEM. **b,** FTIR. **c,** XRD.

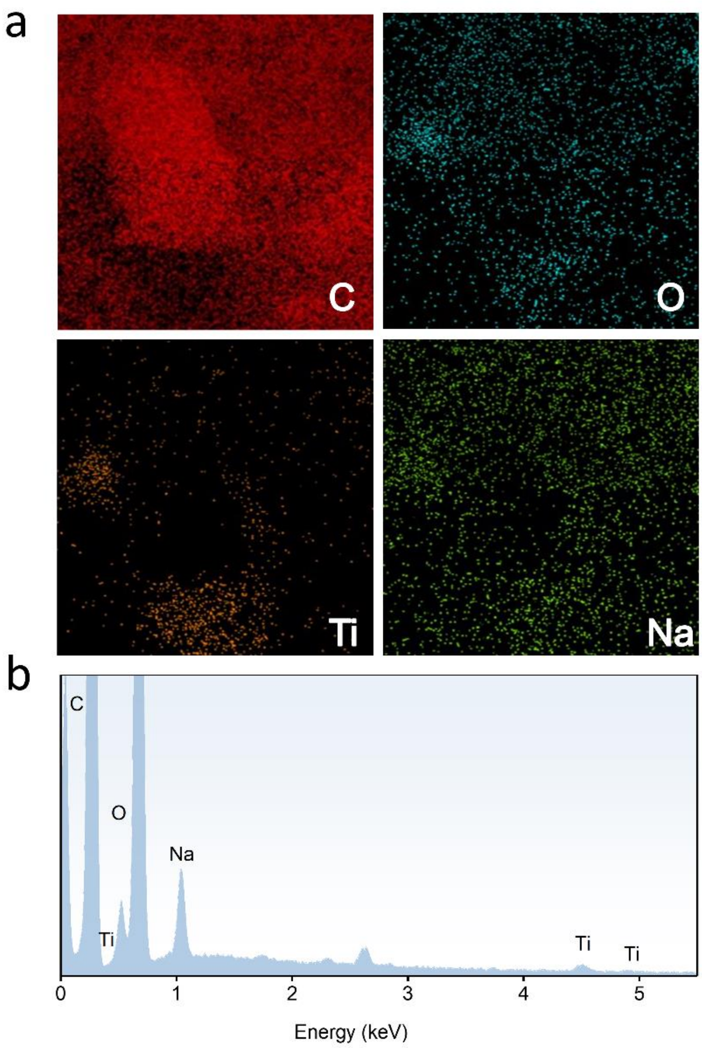

Supplementary Figure 21. Morphology characterization of Ti-MOF on GDE after CER in 5 M NaCl at pH=13. a, EDS mappings. **b,** Element percentages from EDS.

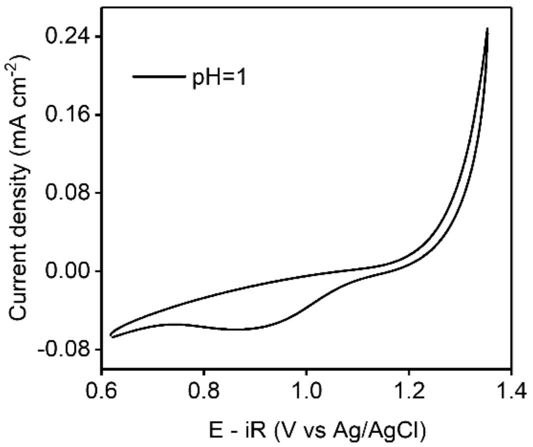

Supplementary Figure 22. Electrochemical data (CV) of Ti-MOF in 5 M NaCl at pH=1 and test compensation for 90%.

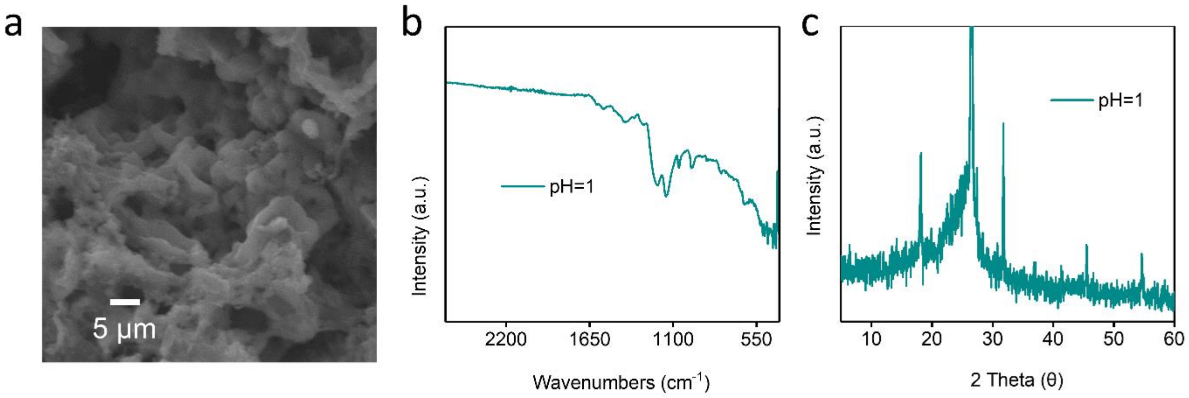

Supplementary Figure 23. Characterization of Ti-MOF on GDE after CER in 5 M NaCl at pH=1. a, SEM. **b,** FTIR. **c,** XRD.

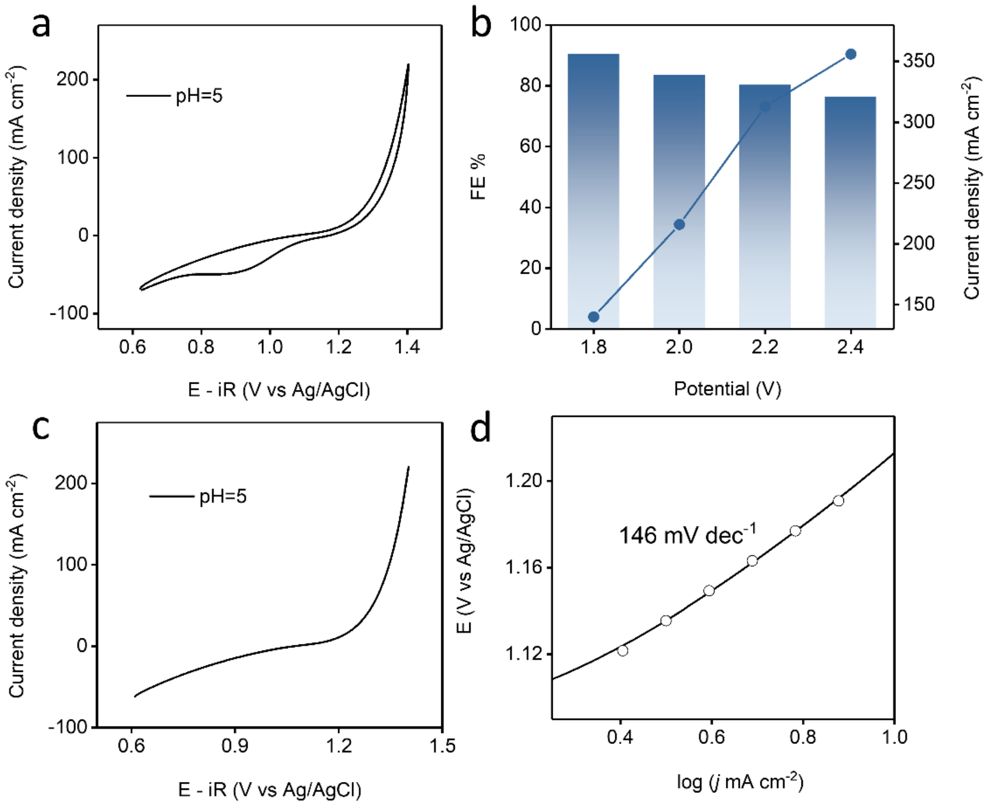

Supplementary Figure 24. Electrochemical data of Ti-MOF in 5 M NaCl at pH=5. a, CV. **b,** Cl_2 Faradic efficiencies and current densities from 1.8 to 2.4 V (*vs.* Ag/AgCl). **c,** LSV. d, Tafel slope from LSV, all except b are compensated at 90%.

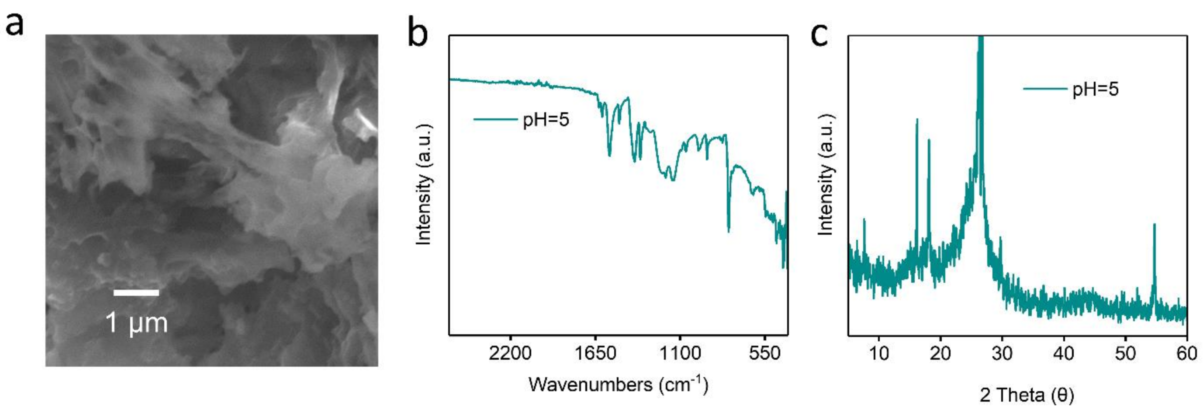

Supplementary Figure 25. Characterization of Ti-MOF on GDE after CER in 5 M NaCl at pH=5. a, SEM. **b,** FTIR. **c,** XRD.

.

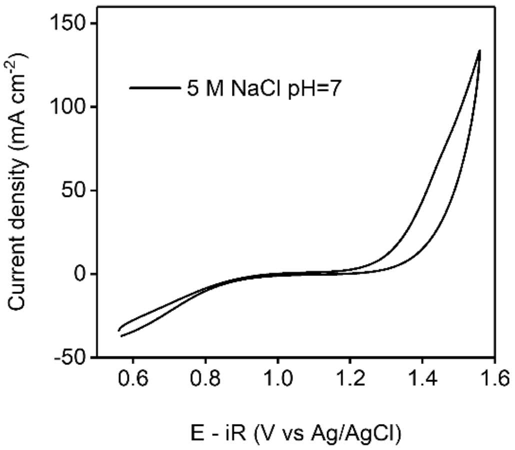

Supplementary Figure 26. Electrochemical data (CV) of Ti-MOF in 5 M NaCl at pH=1 and test compensation for 90%.

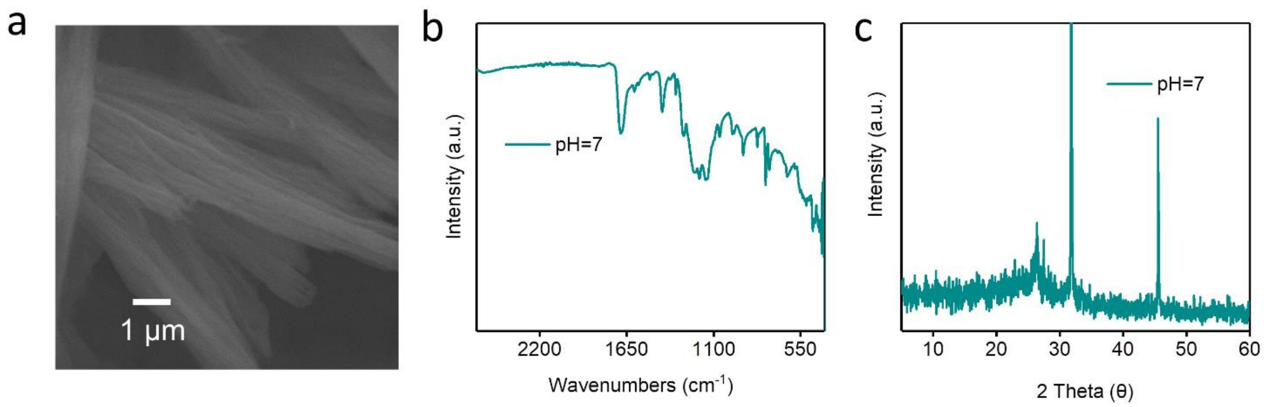

Supplementary Figure 27. Characterization of Ti-MOF on GDE after CER in 5 M NaCl at pH=7. a, SEM. **b,** FTIR. **c,** XRD.

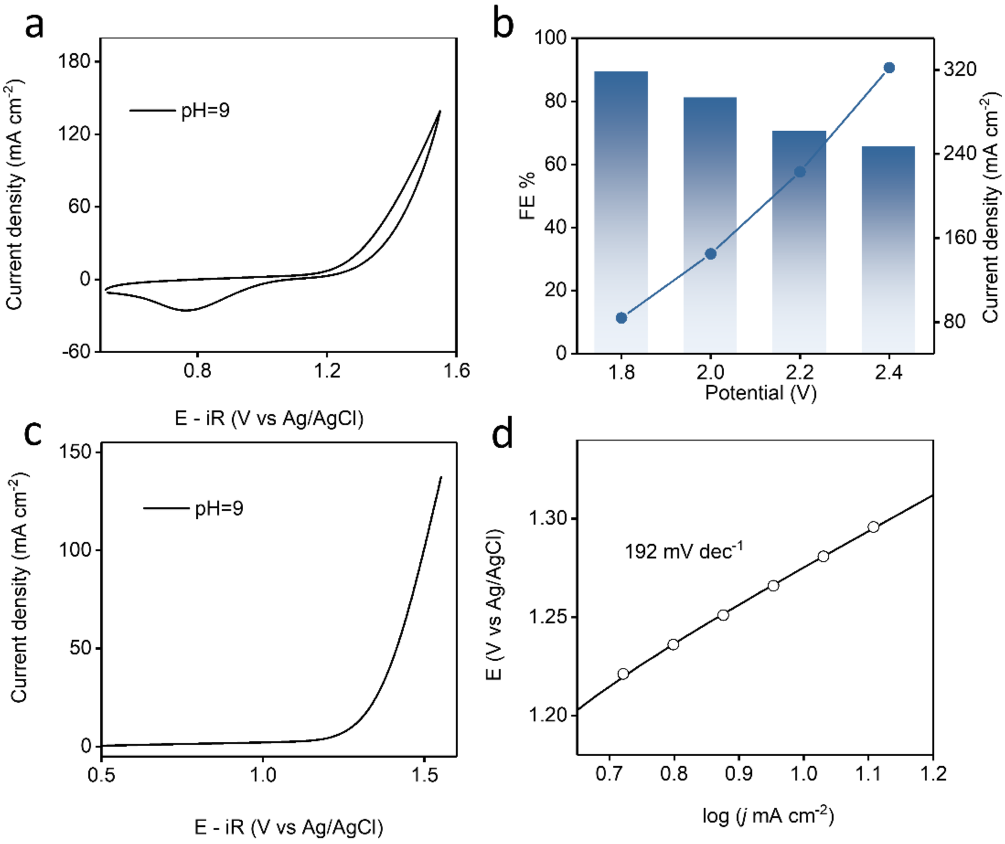

Supplementary Figure 28. Electrochemical data of Ti-MOF in 5 M NaCl at pH=9. a, CV. **b,** Cl_2 Faradic efficiencies and current densities from 1.8 to 2.4 V (*vs.* Ag/AgCl). **c,** LSV. d, Tafel slope from LSV, all except b are compensated at 90%.

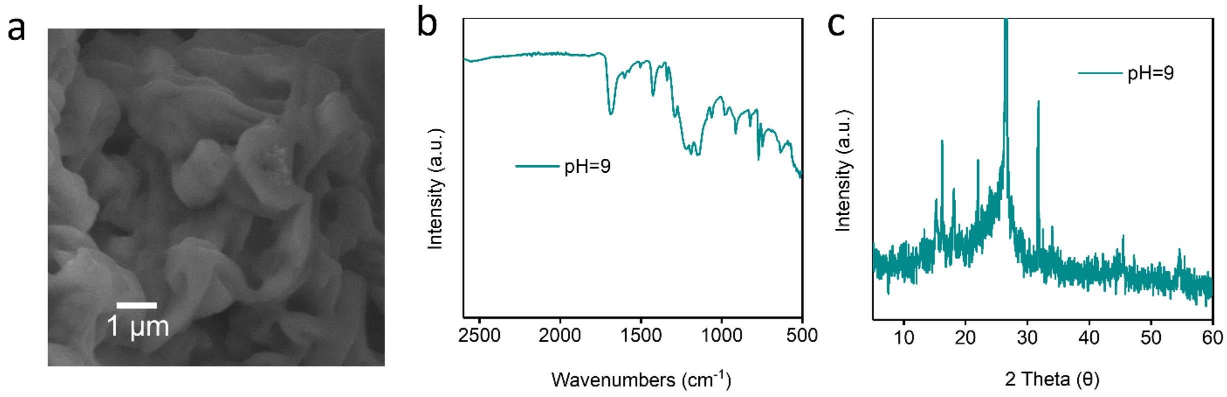

Supplementary Figure 29. Characterization of Ti-MOF on GDE after CER in 5 M NaCl at pH=9. a, SEM. **b,** FTIR. **c,** XRD.

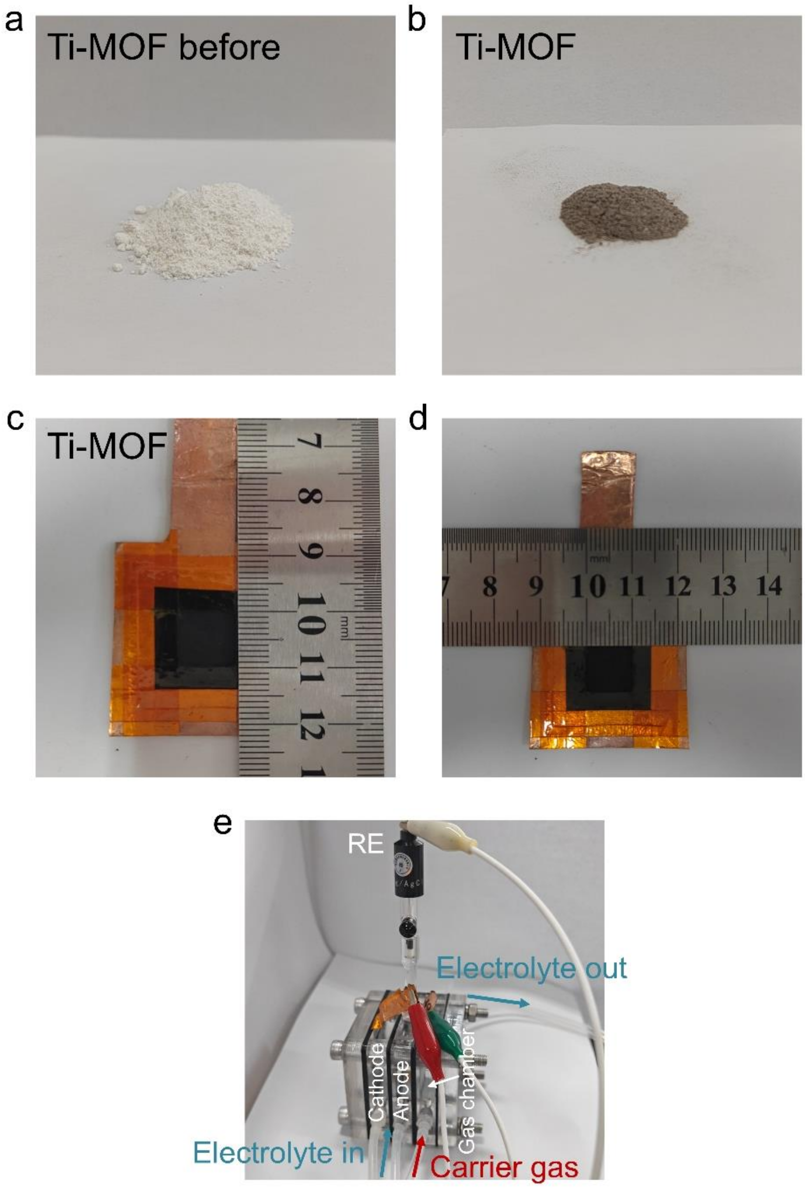

Supplementary Figure 30. Optical pictures of the synthetic processes and electrode preparation. **a-b,** Optical pictures of Ti-MOF-before and Ti-MOF. **c-d,** electrode have an effective active area of 1×1 cm^2. **e,** Electrolysis reaction schematic diagram.

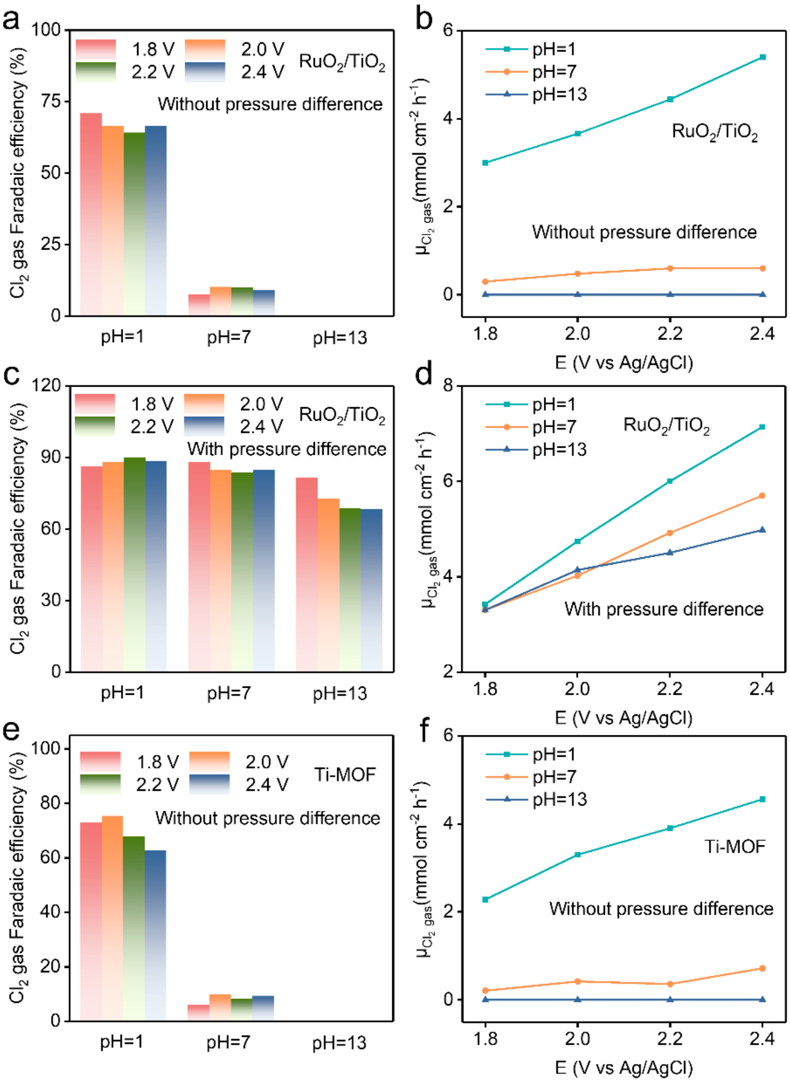

Supplementary Figure 31. Comparison experiments for different samples. a-d, The Cl_2 Faradic efficiencies and yield rates without pressure difference for GDE loaded (RuO_2/TiO_2) as a surrogate of DSA. **c-d,** Cl_2 Faradic efficiency and yield rate of Ti-MOF materials without pressure difference.

We have tried to tune the pressure difference for DSA-based system, however, cannot achieve this operation. This is because of DSA fabricated by coating bulk Ti substrate with electrocatalytic metal oxide layers (*e.g.*, RuO_2, IrO_2)[18] The bulk Ti substrate does not allow gas penetration at three-phase boundary during electrochemical reaction. Consequently, we have replaced Ti substrate of DSA with gas diffusion electrode (GDE), and synthesized the electrode of metal oxide RuO_2/TiO_2@GDE.

Particularly, we have tested the performance of RuO_2/TiO_2@GDE electrode under the same condition. Without pressure difference, the RuO_2/TiO_2@GDE electrode has demonstrated diminished activities with elevated pH values, *i.e.*, Faradaic efficiency of 70.9% (yield rate of 3 mmol cm^{-2} h^{-1}) at pH =1, 7.3% (yield rate of 0.3 mmol cm^{-2} h^{-1}) at pH =7 and 0% (yield rate of 0 mmol cm^{-2} h^{-1}) at pH =13. In great contrast, with pressure difference, the activities of RuO_2/TiO_2@GDE increased significantly, *i.e.*, Faradaic efficiency of 86.3% (yield rate of 3.42 mmol cm^{-2} h^{-1}) at pH =1, 88% (yield rate of 3.3 mmol cm^{-2} h^{-1}) at pH =7 and 81.4% (yield rate of 3.3 mmol cm^{-2} h^{-1}) at pH =13.

The same phenomenon has also been observed for Ti-MOF. Without pressure difference, Ti-MOF has demonstrated diminished activities with elevated pH values, *i.e.*, Faradaic efficiency of 72.8% (yield rate of 2.28 mmol cm^{-2} h^{-1}) at pH =1, 5.8% (yield rate of 0.21 mmol cm^{-2} h^{-1}) at pH =7 and 0% (yield rate of 0 mmol cm^{-2} h^{-1}) at pH =13. In great contrast, with pressure difference, the activities of Ti-MOF increased significantly, *i.e.*, Faradaic efficiency of 93.4% (yield rate of 1.68 mmol cm^{-2} h^{-1}) at pH =1, 94% (yield rate of 0.93 mmol cm^{-2} h^{-1}) at pH =7 and 88.8% (yield rate of 0.78 mmol cm^{-2} h^{-1}) at pH =13.

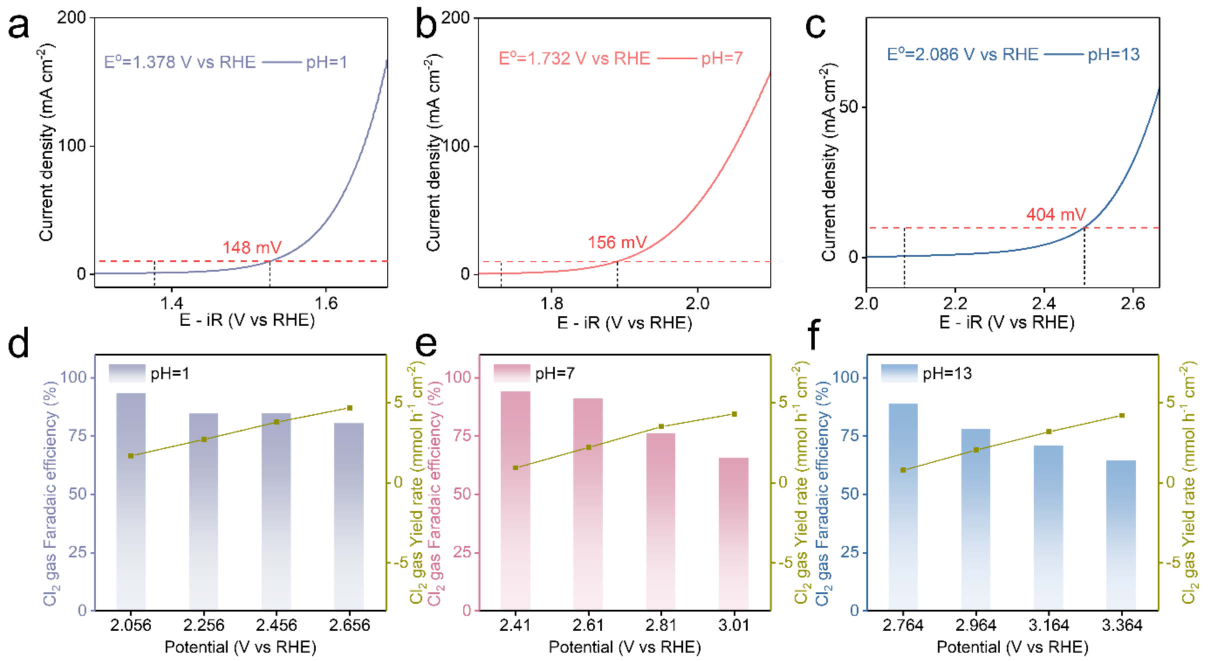

Supplementary Figure 32. Electrochemical performance data of Ti-MOF by potential conversion to reversible hydrogen electrode (RHE) and test compensation for 90%. **a-c,** LSV. **d-f,** Faradaic efficiency and gas production rate.

According to the standard electrochemical principle, the conversion relationship between the potential of Ag/AgCl electrode (*vs* Ag/AgCl) and RHE is (at 25 °C):

$$E_{RHE} = E_{Ag/AgCl} + 0.059 \times pH + E^{o}_{Ag/AgCl} \tag{11}$$

Here, $E^{o}_{Ag/AgCl}$ is the standard electrode potential of Ag/AgCl, which is approximately 0.197 V in a saturated KCl solution. From this equation, the potential at each pH can be calculated.

Equation $2Cl^{-} - 2e^{-} \rightarrow Cl_2$, E^0=1.36 V *vs.* RHE is the standard electrode potential measured at pH = 0. However, in the actual situation of this work, further calculations are required:

$$E_{eq} = E^{o} + \frac{RT}{nF} ln \frac{[p^{\theta}_{Cl_2}]}{[Cl^{-}]^2} + E^{o}_{Ag/AgCl} \tag{12}$$

In the equation, *R is* gas constant (8.314 J mol^{-1} K^{-1}), *T:* absolute temperature (K), *n*: number of transferred electrons, *F*: Faraday constant (96485 C mol^{-1}), $[p^{\theta}_{Cl_2}]$: partial pressure of chlorine gas (is the constant 1 bar), $[Cl^{-}]^2$: chloride ion concentration (5 mol L^{-1}) and the $E^{o}_{Ag/AgCl}$ is the standard electrode potential of the reference electrode (is the constant 0.197 V) Substituting into the formula and calculating, we obtain Eeq=1.3186 V *vs.* SHE. And by converting the SHE and RHE

potentials, we can obtain:

$$E_{RHE} = E_{SHE} + 0.059 \times pH \tag{13}$$

The standard electrode potentials at pH values of 1, 7 and 13 can be calculated:

$$E_{RHE} = 1.3186 + 0.059 \times 1 = 1.3777\, V\ vs\ RHE\ (pH = 1)$$

$$E_{RHE} = 1.3186 + 0.059 \times 7 = 1.7316\, V\ vs\ RHE\ (pH = 7)$$

$$E_{RHE} = 1.3186 + 0.059 \times 13 = 2.0856\, V\ vs\ RHE\ (pH = 13)$$

From above equations, we can calculate the overpotential of Ti-MOF at each pH value. According to the formula:

$$\eta = E_{measured} - E_{theoretical} \tag{14}$$

It can be calculated that: $\eta_{pH=1} = 1.5259 - 1.3777 = 0.1482\, V\ vs\ RHE$; $\eta_{pH=7} = 1.888 - 1.7316 = 0.1482\, V\ vs\ RHE$, $\eta_{pH=13} = 2.49 - 2.0856 = 0.404\, V\ vs\ RHE$.

By comparing with the state-of-the-art literature in Supplementary Table 3, we can see almost all of the previous studies have operated in strong acid electrolyte (pH <3), and used membrane-based systems and noble-metal catalysts. Under such harsh testing condition, the overpotentials for most previous studies (at 10 mA cm^{-2}) are still in the range of 30~220 mV, which is comparable to our system with membrane-free configuration and non-precious metal catalyst (148 mV at pH=1; 156 mV at pH=7).

To the best of our knowledge, there is no study operating in strong alkaline condition (pH = 13) because of the Pourbaix diagram limitation. It is common concept that no Cl_2 can evolve from alkaline electrochemical system because of chemical reaction between Cl_2 and alkaline ($Cl_2 + 2OH^- \rightarrow 2Cl^- + H_2O$). In this work, our system can operate at strong alkaline condition (pH = 13) for chlorine evolution with overpotentials comparable to some previously reported studied in acidic condition.[19,20]

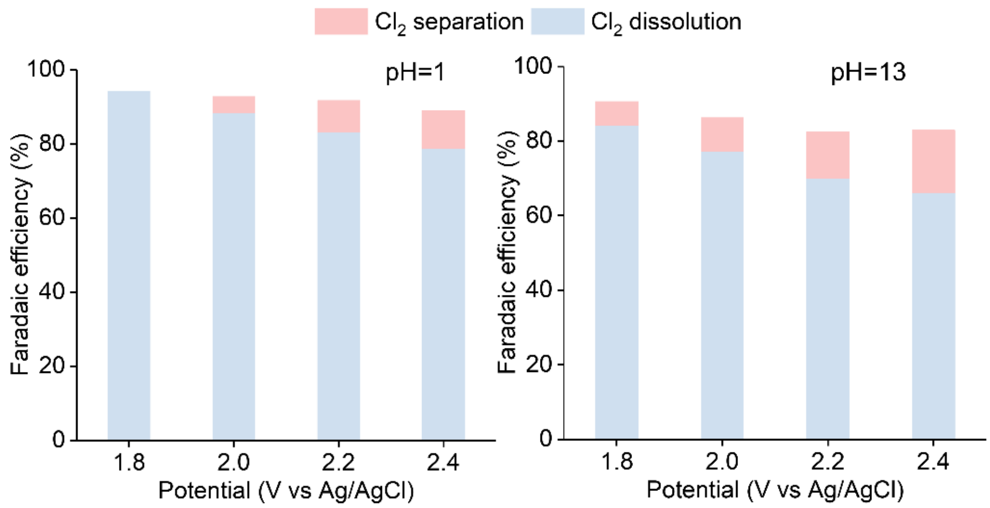

Supplementary Figure 33. Performance comparison of Cl_2 separation and Cl_2 dissolution under different pH conditions.

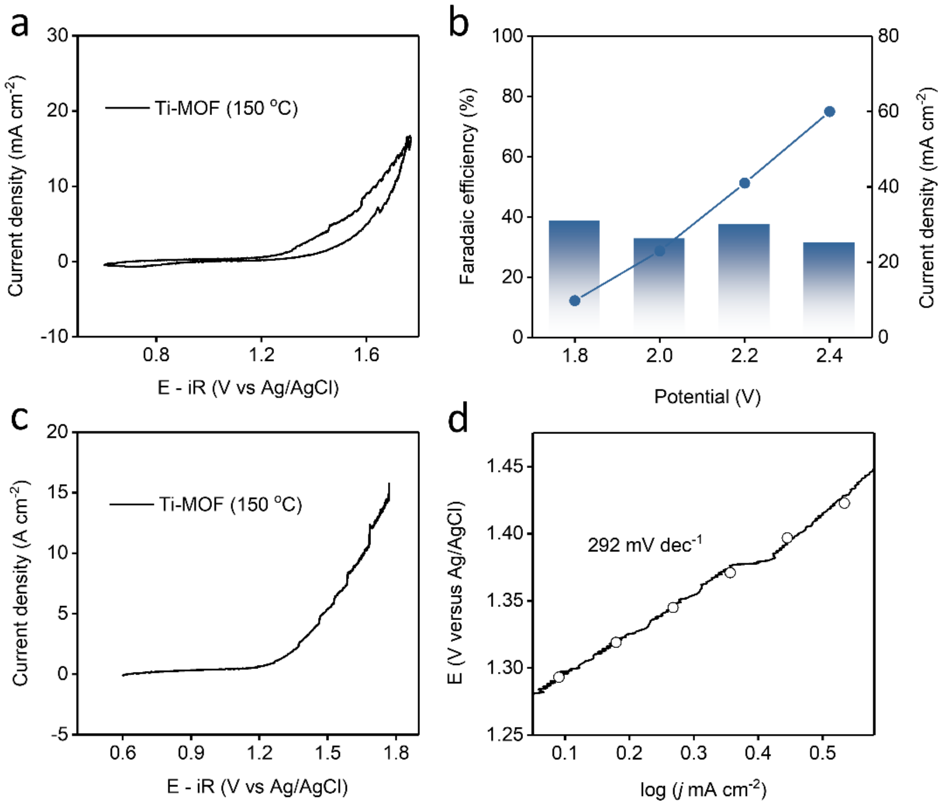

Supplementary Figure 34. Electrochemical data of Ti-MOF (150 °C) in 5 M NaCl at pH=13. a, CV. **b,** Cl_2 Faradic efficiencies and current densities from 1.8 to 2.4 V (*vs.* Ag/AgCl). **c,** LSV. **d,** Tafel slope from LSV, all except b are compensated at 90%.

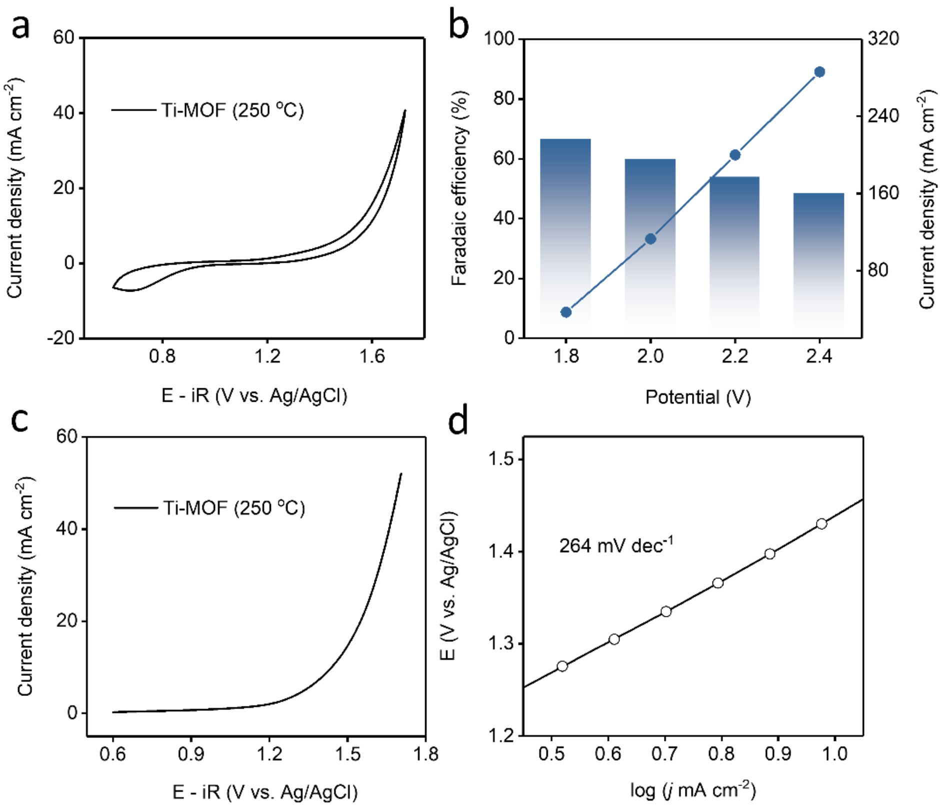

Supplementary Figure 35. Electrochemical data of Ti-MOF (250 °C) in 5 M NaCl at pH=13. a, CV. **b,** Cl_2 Faradic efficiencies and current densities from 1.8 to 2.4 V (*vs.* Ag/AgCl). **c,** LSV. **d,** Tafel slope from LSV, all except b are compensated at 90%.

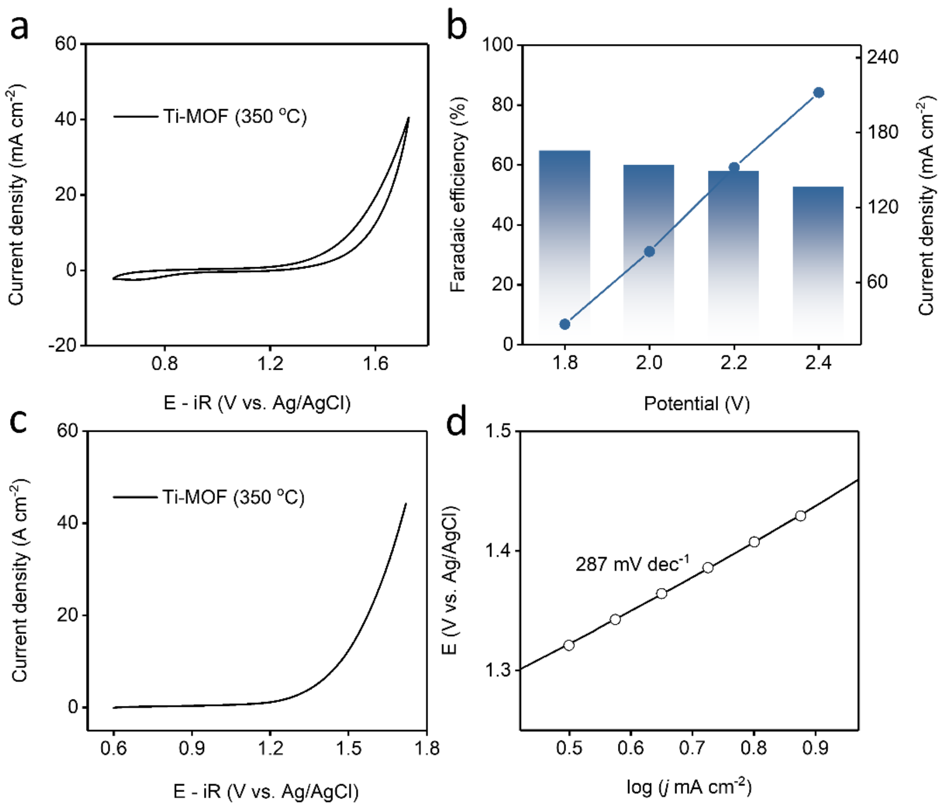

Supplementary Figure 36. Electrochemical data of Ti-MOF (350 °C) in 5 M NaCl at pH=13. a, CV. **b,** Cl_2 Faradic efficiencies and current densities from 1.8 to 2.4 V (*vs.* Ag/AgCl). **c,** LSV. **d,** Tafel slope from LSV, all except b are compensated at 90%.

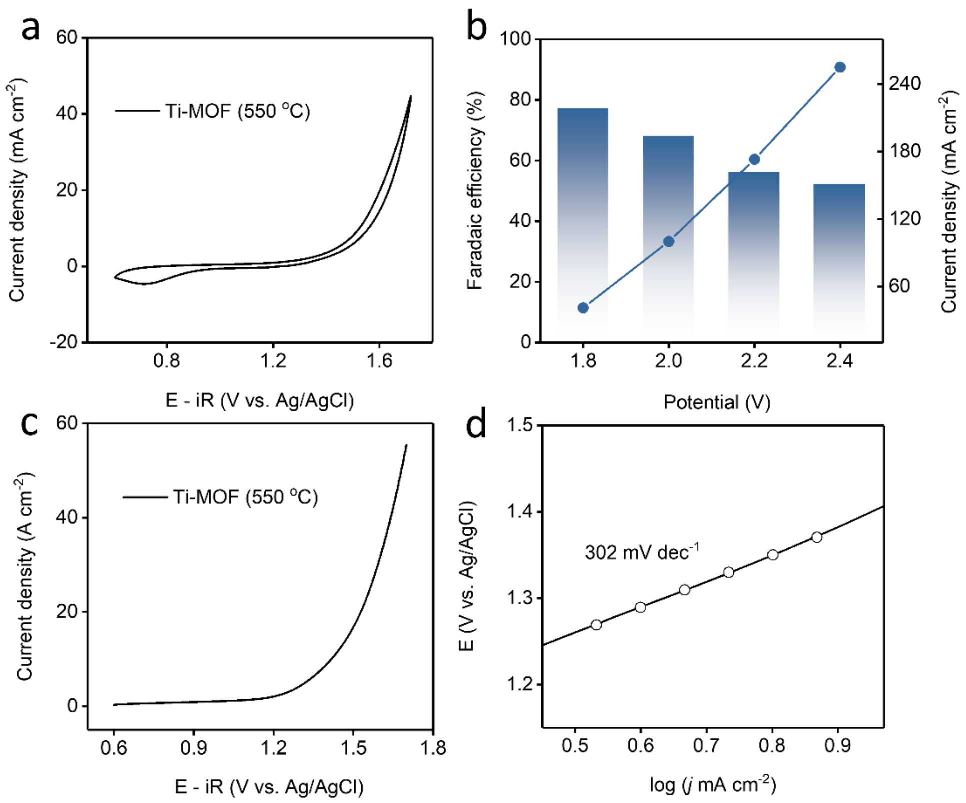

Supplementary Figure 37. Electrochemical data of Ti-MOF (550 °C) in 5 M NaCl at pH=13. a, CV. **b,** Cl_2 Faradic efficiencies and current densities from 1.8 to 2.4 V (*vs.* Ag/AgCl). **c,** LSV. **d,** Tafel slope from LSV, all except b are compensated at 90%.

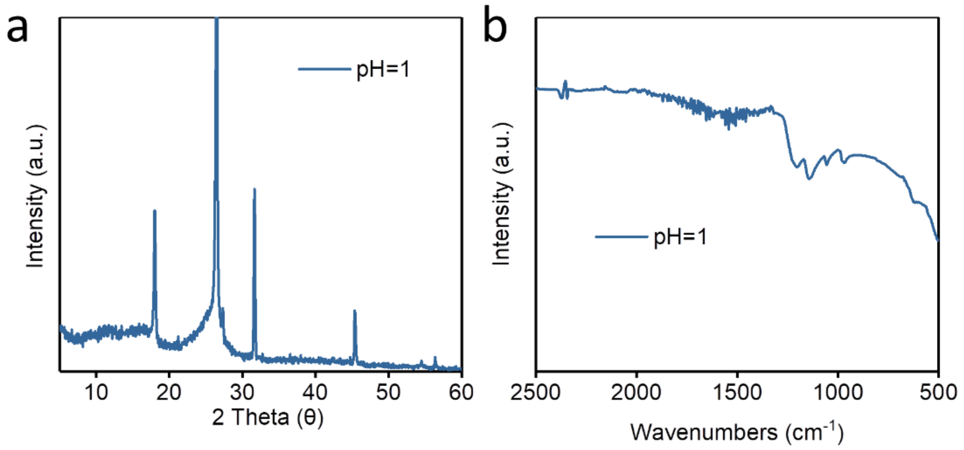

Supplementary Figure 38. Characterization of Ti-MOF on GDE after 50-hrs CER test in 5 M NaCl at pH=1. a, XRD. **b,** FTIR.

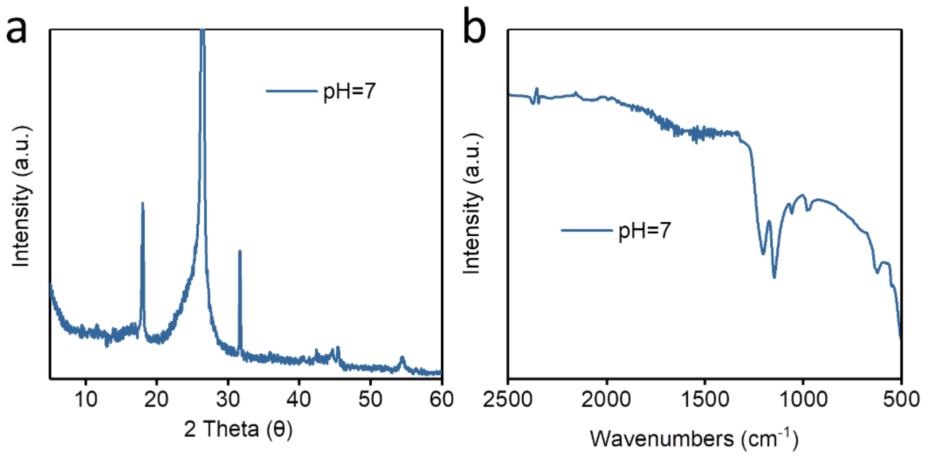

Supplementary Figure 39. Characterization of Ti-MOF on GDE after 50-hrs test in 5 M NaCl at pH=7. a, XRD. **b,** FTIR.

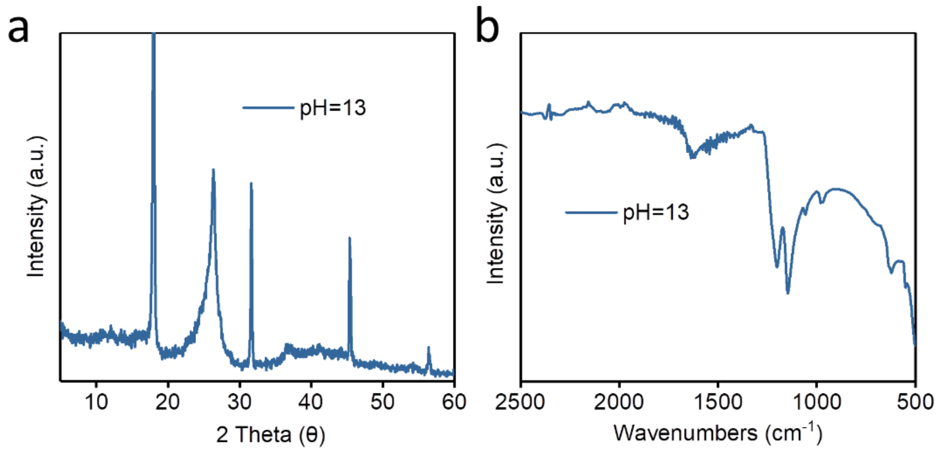

Supplementary Figure 40. Characterization of Ti-MOF on GDE after 50-hrs test in 5 M NaCl at pH=13. a, XRD. **b,** FTIR.

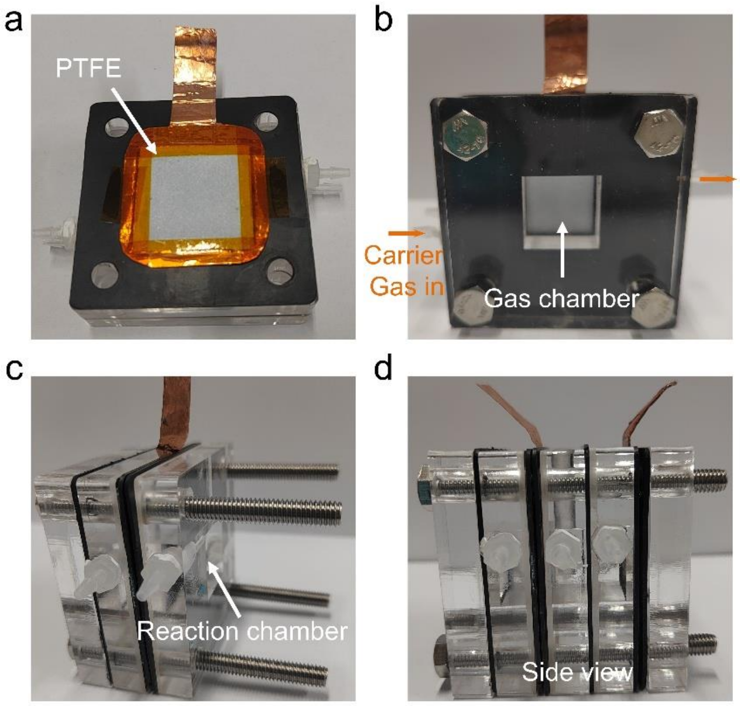

Supplementary Figure 41. a, working electrode assembly. **b,** anode/gas chamber unit. **c,** reaction chamber. **d,** Overview of reactor configuration.

Supplementary Figure 41a shows a photograph of working electrode assembly, with white PTFE hydrophobic membrane laminated on the backside. Supplementary Figure 41b presents the assembled anode/gas chamber unit, where the carrier gas flow path is indicated by orange arrows (inlet/outlet). Supplementary Figures 41c, d display the reaction chamber diagram.

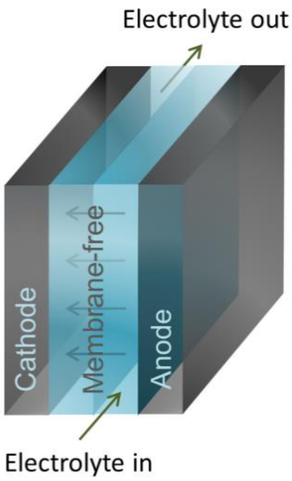

Supplementary Figure 42. Structure diagram of membrane-free devices

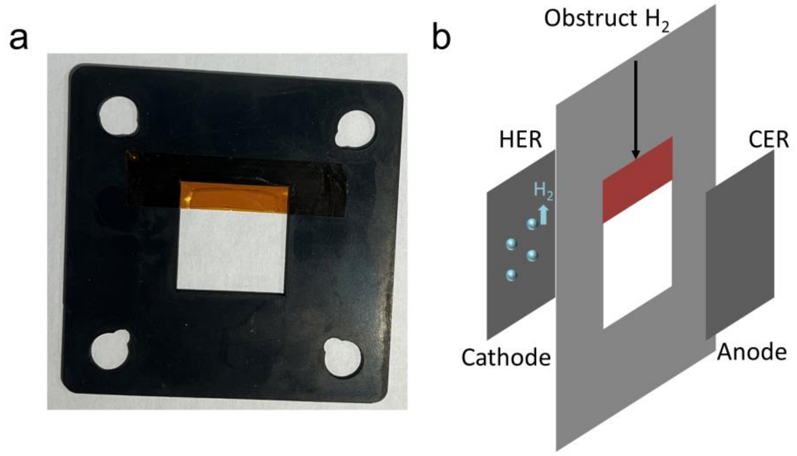

Supplementary Figure 43. Internal details and demonstration for membrane-free devices. a, Adhesive insulation tape part of the gasket exposed to the window. **b,** Block generated hydrogen gas demonstration diagram.

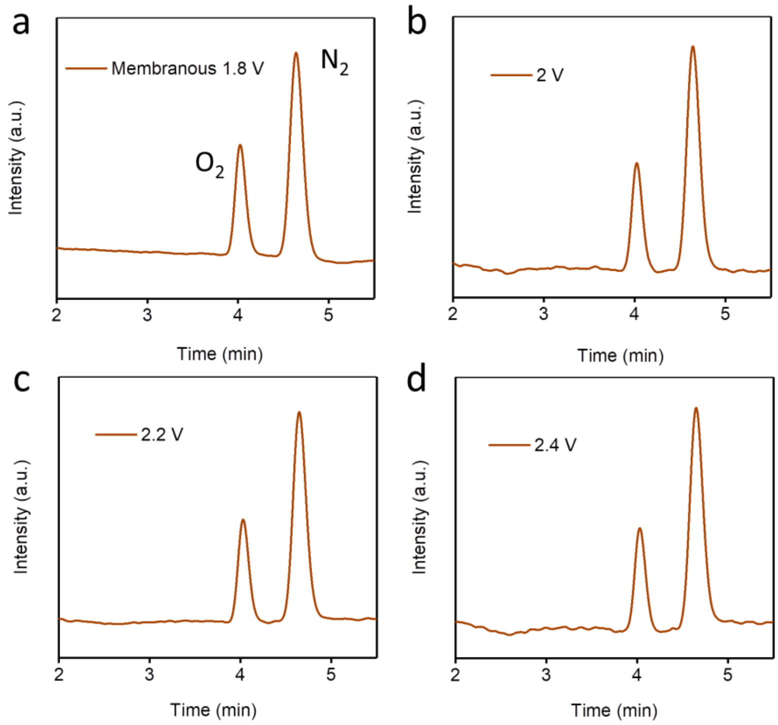

Supplementary Figure 44. Gas chromatography data for Ti-MOF in membrane-based device.

a-d, From 1.8 to 2.4 V *vs.* Ag/AgCl.

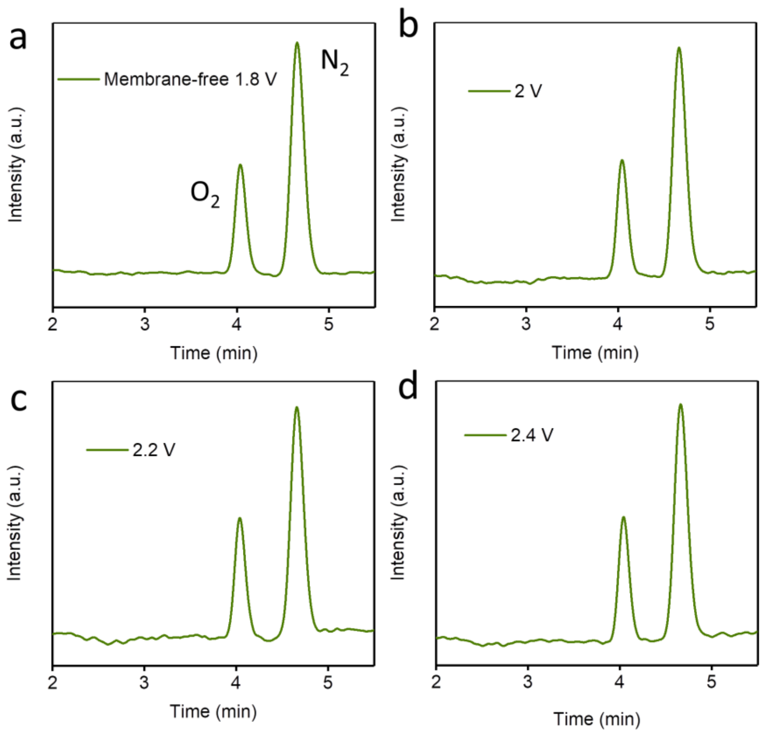

Supplementary Figure 45. Gas chromatography data for Ti-MOF in membrane-free device. a-d, From 1.8 to 2.4 V *vs.* Ag/AgCl.

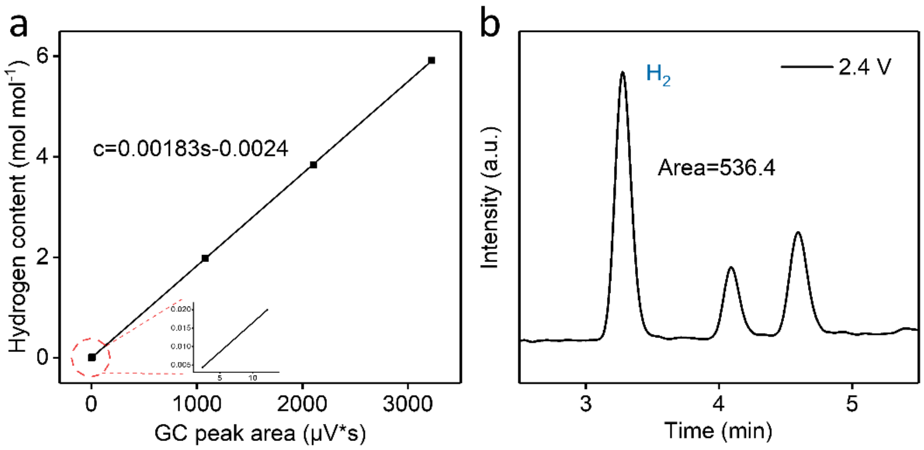

Supplementary Figure 46. Gas-phase testing of hydrogen. a, Standard curve of hydrogen gas. b, Gas chromatography (GC) profile of cathodic products.

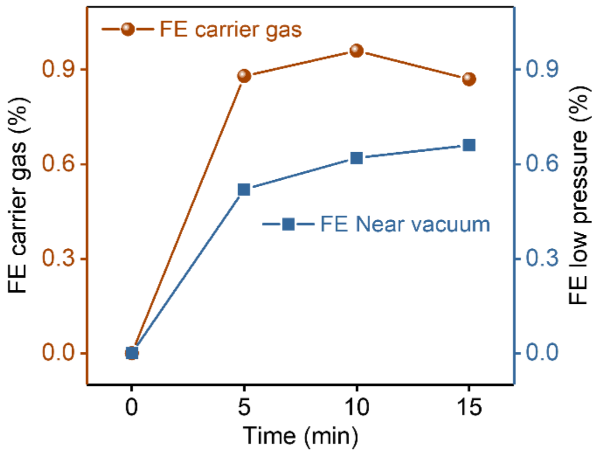

Supplementary Figure 47. Cl_2 Faraday efficiency (FE) by using carrier gas (CO_2) and vacuum extraction methods.

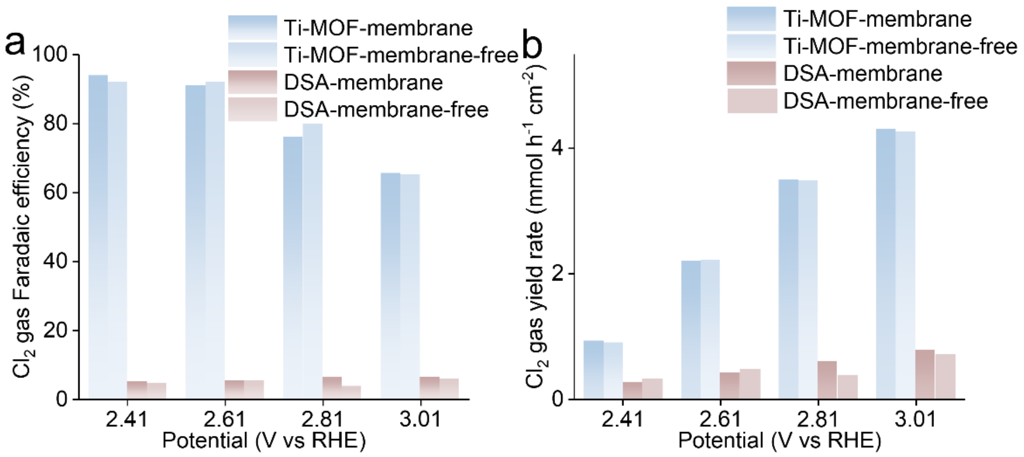

Supplementary Figure 48. Electrochemical data of Ti-MOF and DSA after potential conversion (*vs.* RHE). a, Faradaic efficiency. **b,** yield rate.

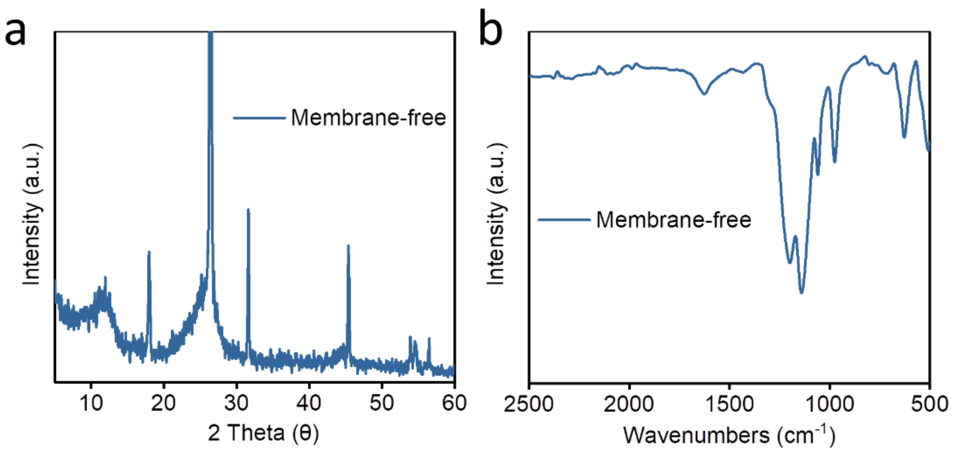

Supplementary Figure 49. Characterization of Ti-MOF on GDE after 200-hrs CER test in 5 M NaCl. a, XRD. **b,** FTIR.

Based on above experimental data, we conclude that Ti-MOF is a stable metal-organic framework material. One of the reasons is that the voltage applied to chlorine evolution reaction is positive, which makes it difficult to further oxidize Ti-MOF, as it already in a relatively high oxidation state.[21] Similar to previous literature, Ti-based MOF materials are often used as stable active materials in catalytic oxidation reactions.[22] Secondly, the strong Ti-O coordination interaction inside Ti-MOF also stabilizes the structure, especially the Ti-MOF has been subjected to a low-temperature calcination to enhance its crystallinity (Figure 2b) in our work.[23] Therefore, we believe that Ti-MOF exhibits excellent stability under harsh electrochemical conditions.

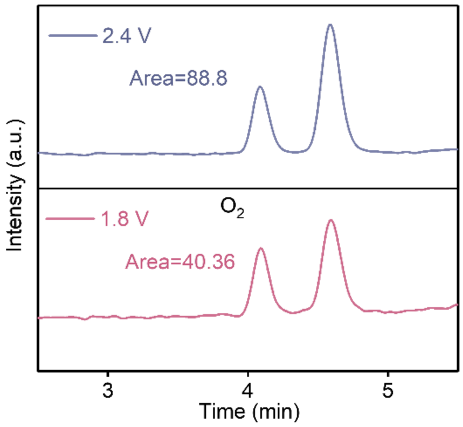

Supplementary Figure 50. GC data of O_2 under 1.8V and 2.4V conditions.

We have conducted additional experiment of determining O_2 byproduct from side OER. At different applied potentials, O_2 was collected and measured by gas chromatography (GC, Supplementary Figure 50). We have annotated the GC peak areas of O_2 as 40.36 at 1.8 V, which has doubled at 2.4 V (*i.e.*, 88.8), respectively. This result confirms an increase in O_2 levels with rising applied potentials, thereby reducing overall efficiency of Cl_2 production.

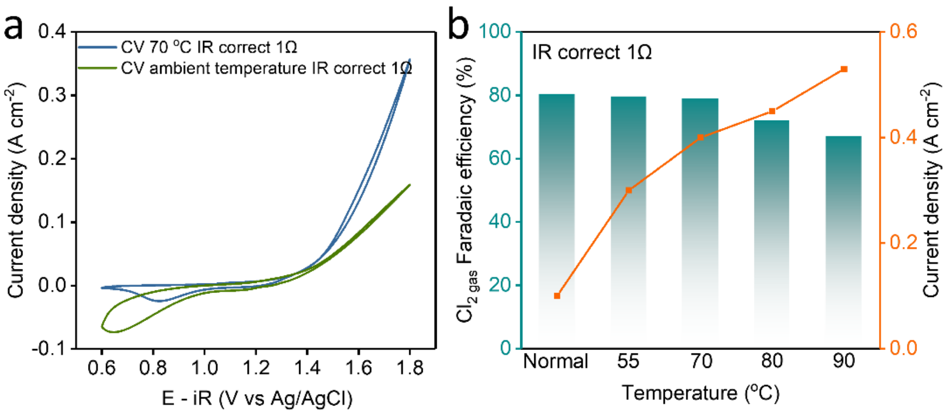

Supplementary Figure 51. Electrochemical data of Ti-MOF tested in 0.1 M NaOH+5 M NaCl.

a, CV curves at different temperatures under IR correct compensation at 1Ω. **b,** Cl_2 gas Faradic efficiencies and current density at different temperatures under IR correct compensation at 1Ω.

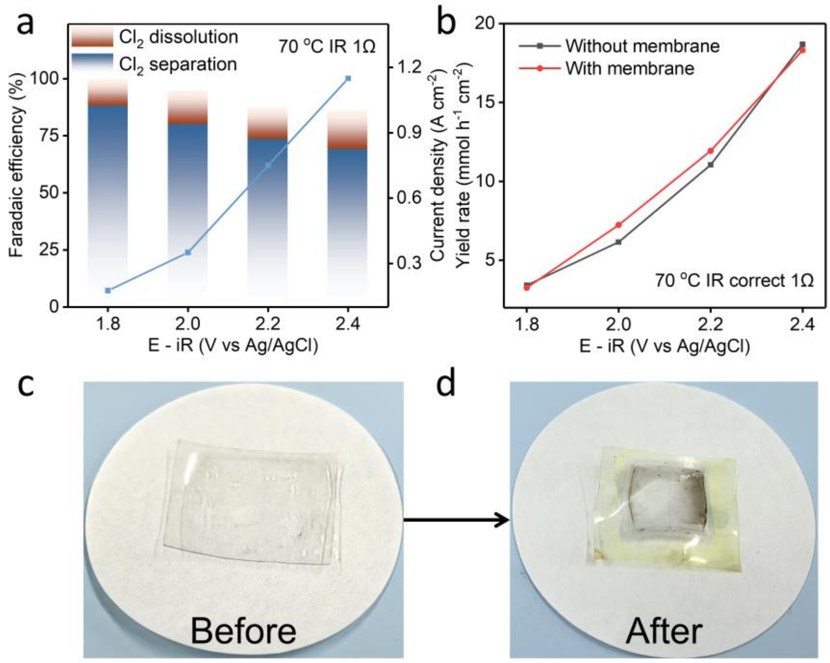

Supplementary Figure 52. Electrochemical data and membrane change of Ti-MOF tested in 0.1 M NaOH+5 M NaCl under IR correct compensation and 70 °C. a, Cl_2 separation and dissolution Faradic efficiencies and current density tested by with membrane device. **b,** Active chlorine yield rate in membrane and membrane-free devices. **c-d,** Changes of membrane before and after reaction in a device with membrane. The ion membrane used is Nafion 117, with a size of 3×3 cm and a thickness of approximately 0.2 mm. It is usually stored in deionized water for daily use. When not in use for a long time, it needs to be soaked alternately in 5% H_2O_2 and 0.1 M H_2SO_4, then rinsed with deionized water.

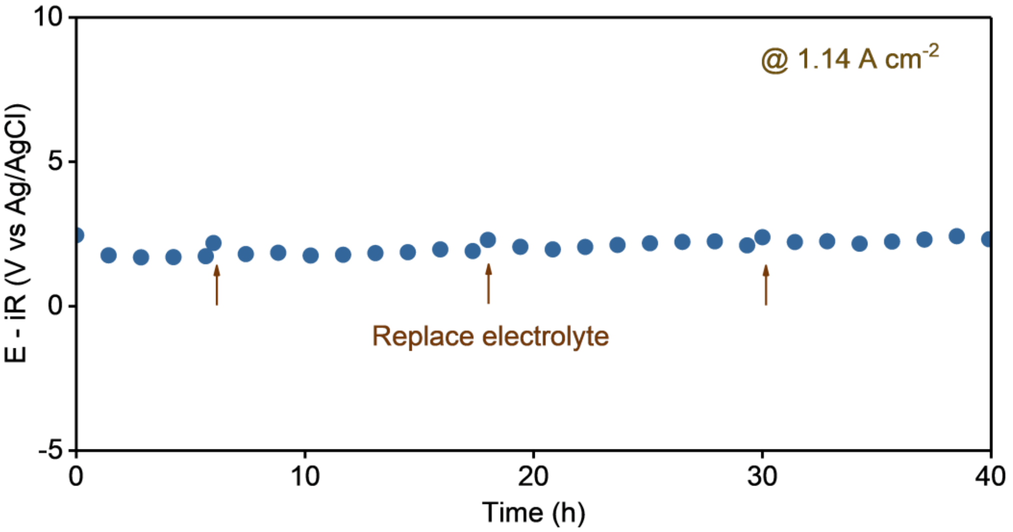

Supplementary Figure 53. Ti-MOF stability experiments at the current density of 1.14 A cm^{-2} under IR correct compensation at 1Ω.

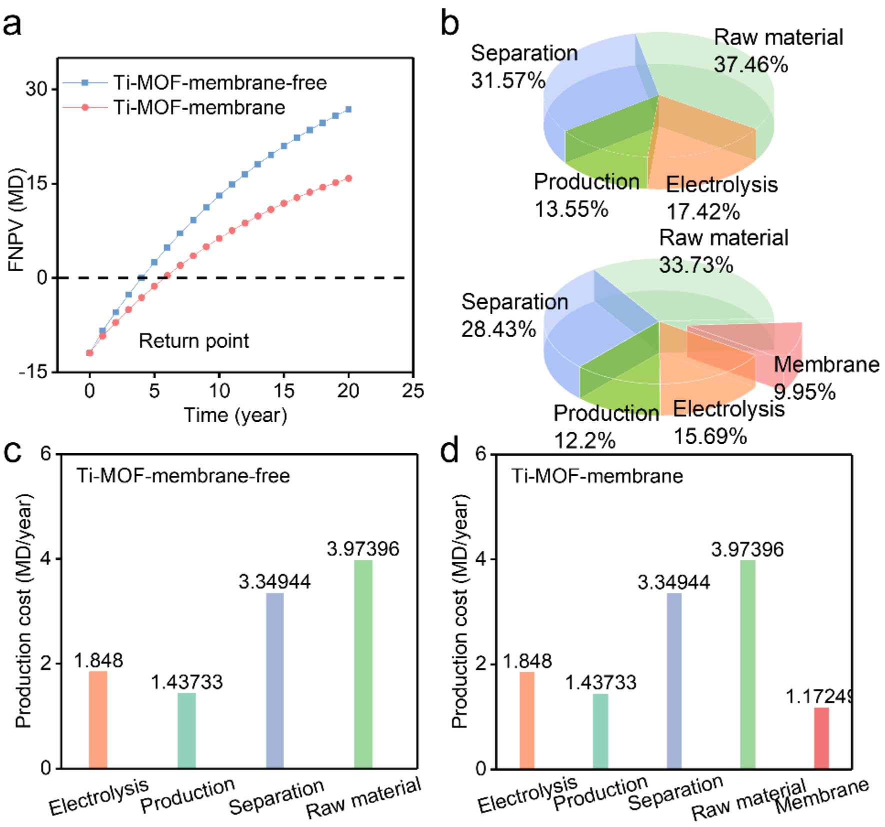

Supplementary Figure 54. Techno-economic analyses for Ti-MOF systems with/without membrane at 0.7 A cm^{-2}. a, FNPV analyses. **b,** Cost distribution percentages in an operating cycle. **c-d,** Production cost distribution in an operating cycle.

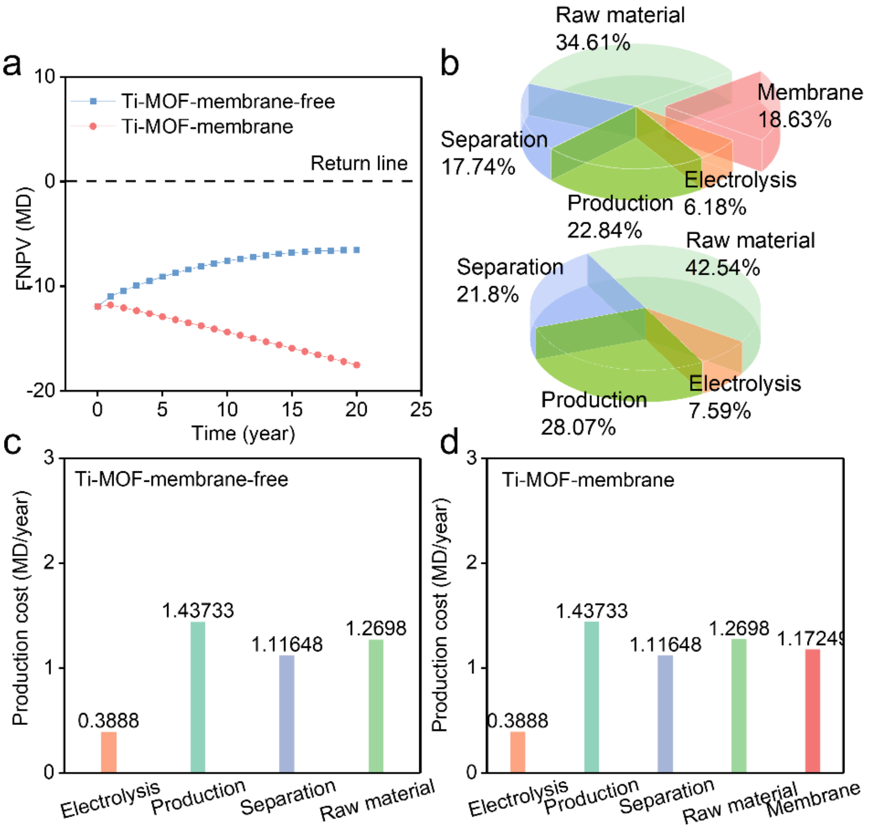

Supplementary Figure 55. Techno-economic analyses for Ti-MOF systems with/without membrane at 0.34 A cm^{-2}. a, FNPV analyses. **b,** Cost distribution percentages in an operating cycle. **c-d,** Production cost distribution in an operating cycle.

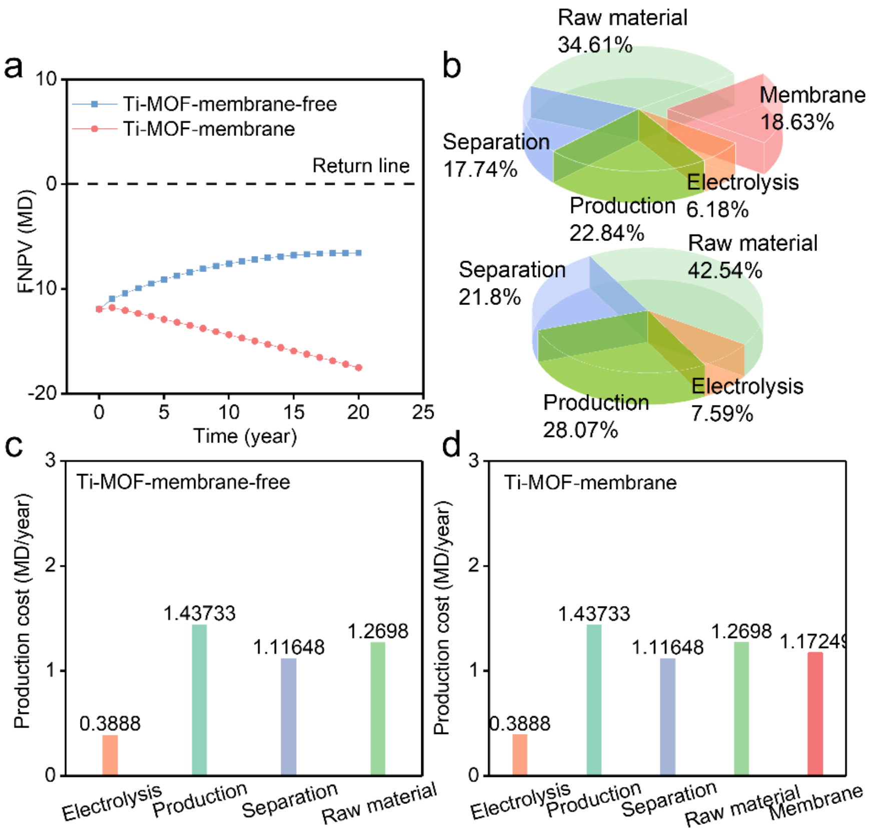

Supplementary Figure 56. Techno-economic analyses for Ti-MOF systems with/without membrane at 0.18 A cm^{-2}. a, FNPV analyses. b, Cost distribution percentages in an operating cycle. c-d, Production cost distribution in an operating cycle.

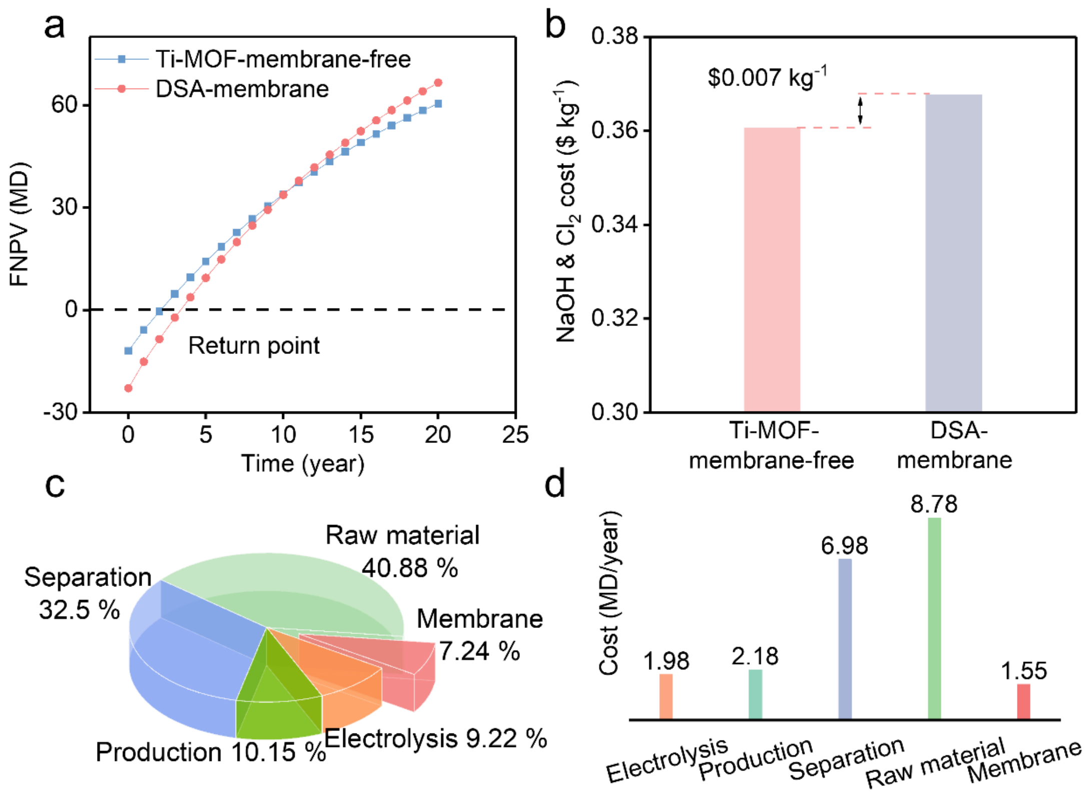

Supplementary Figure 57. Techno-economic analyses. a, FNPV analyses for Ti-MOF-based membrane-free system and DSA-based membrane system. **b,** The production cost of electrochemical production per kilogram of Cl_2 and NaOH for Ti-MOF-based membrane-free system and DSA-based membrane system. **c-d,** cost distribution percentages and production cost distribution in an operating cycle for DSA-based membrane system.

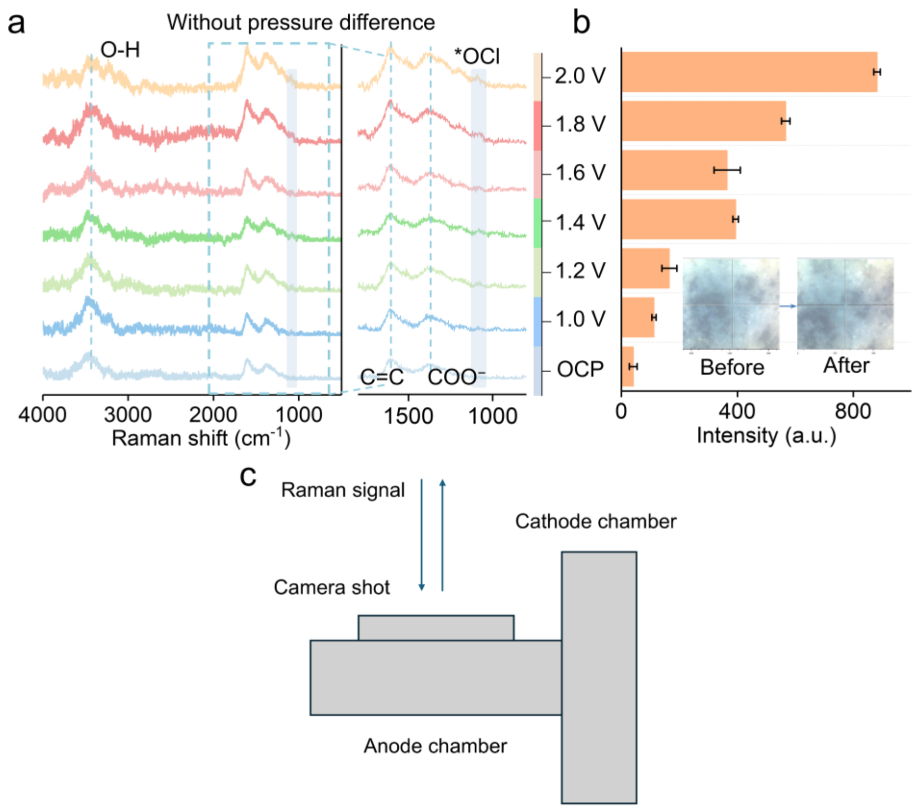

Supplementary Figure 58. The *operando* Raman spectra without pressure difference. a, *operando* Raman spectra. **b,** Ti-*OCl Raman peak intensity. **c,** Schematic diagram of *operando* Raman electrochemical cells testing.

Generally, Raman spectra is a scattering signal with intrinsic weak intensity. When used to study the structural characteristics of a bulk material, obvious signal peaks can be seen. This is due to the structural crystal lattice that contributes to overall vibration of the bulk material, leading to enhanced scattered signals.

While in this work, the *operando* Raman is used to probe the transient surface-adsorbed species (*OCl intermediates) during electrocatalysis. The scattered Raman signals are mainly focused on bond vibrations of adsorbed species on catalyst surfaces, which are known to be very weak as comparison to structural crystal lattices in bulk materials. Consequently, the *operando* Raman signals for catalytic reactions often demonstrate characteristically low signal-to-noise ratios in the literatures

(like ORR, NRR, CRR).[14,24,25] Our *operando* Raman signals are comparable to the above literature.

To confirm the accuracy of the experimental results, we performed five *operando* Raman tests. We directly determined the positions and intensities of the peaks using Raman spectrometer software, and updated the *operando* Raman data with error bars (Figures 5a-b and Supplementary Figure 58). By comparing the Raman vibration at different electrolysis potentials, we conclude the characteristic *OCl Raman peak at approximately 1093 cm^{-1} (inside the blue transparent frame), and the intensity of *OCl peak changes consistently with applied potentials, reaching maximum intensity at 2.0 V vs Ag/AgCl (Figure 5b). Further, the data demonstrate significantly greater accumulation of the *OCl intermediate under zero pressure differential conditions. This observation suggests that the Bernoulli principle leveraged in our reaction system generates an immediate pressure gradient, enabling continuous removal of Cl_2 product and thereby enhancing chlorine evolution kinetics. All of these results provide the validity of our *operando* synthesis condition.

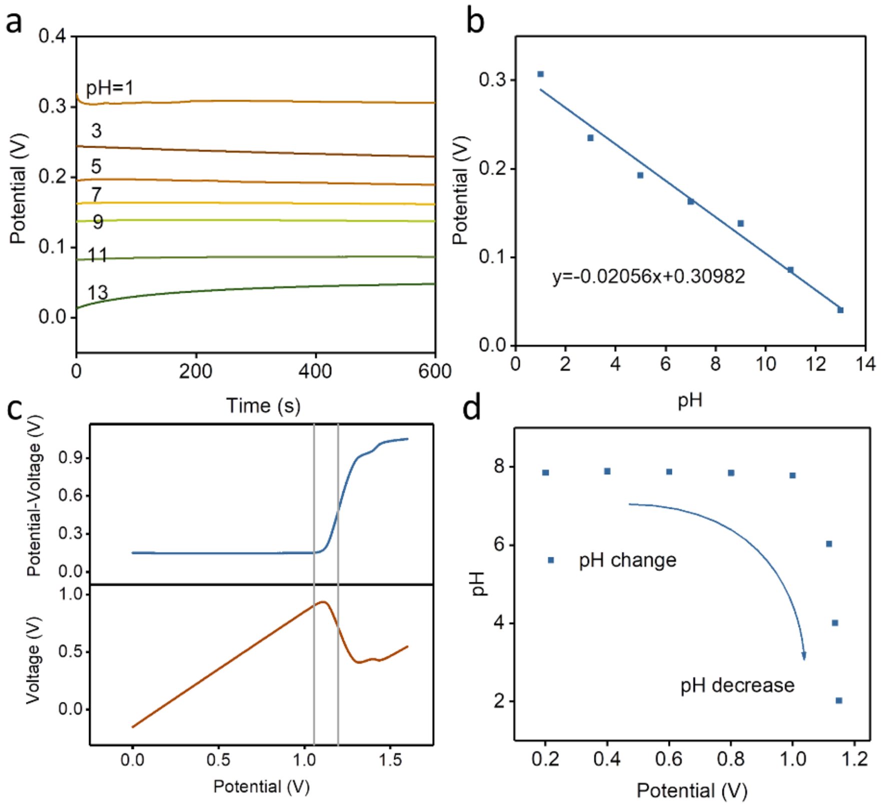

Supplementary Figure 59. Local pH test. a, The calibration curves for local pHs by using RRDE. **b,** pH dependent open circuit potential (E_{oc}) for Pt-ring electrode. The measurement was performed in NaCl, and the pH of the electrolyte was changed by adding HCl or NaOH. **c,** LSVs and corresponding current at different potentials. **d,** pH changes relevant to potentials.

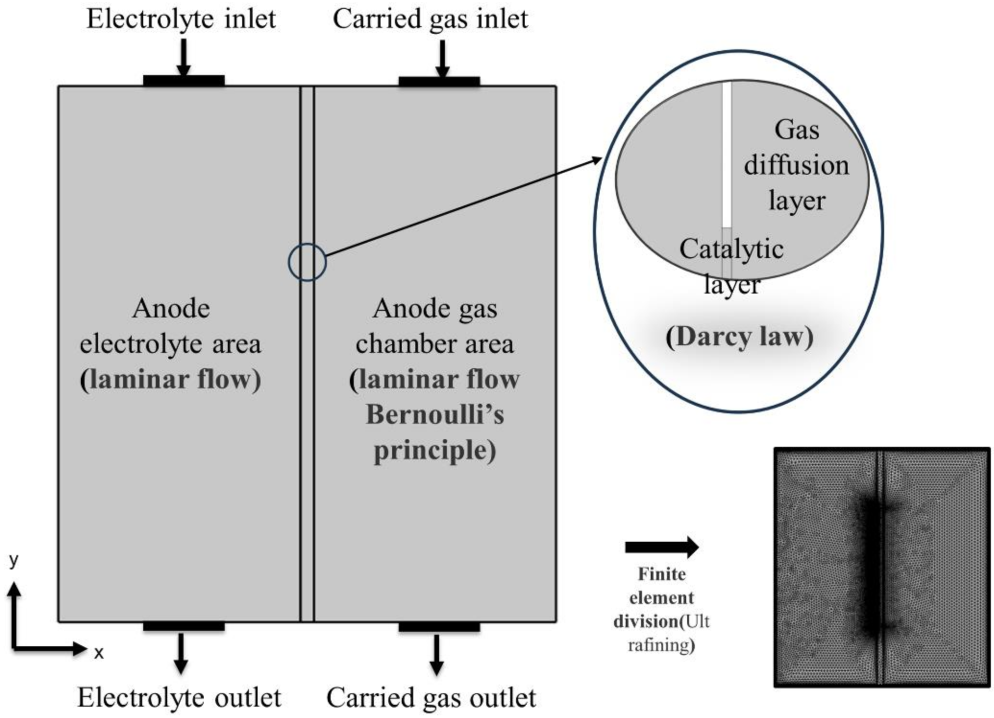

Supplementary Figure 60. Steady-state complete models of anode gas chamber, gas diffusion layer, catalytic layer and electrolyte chamber.

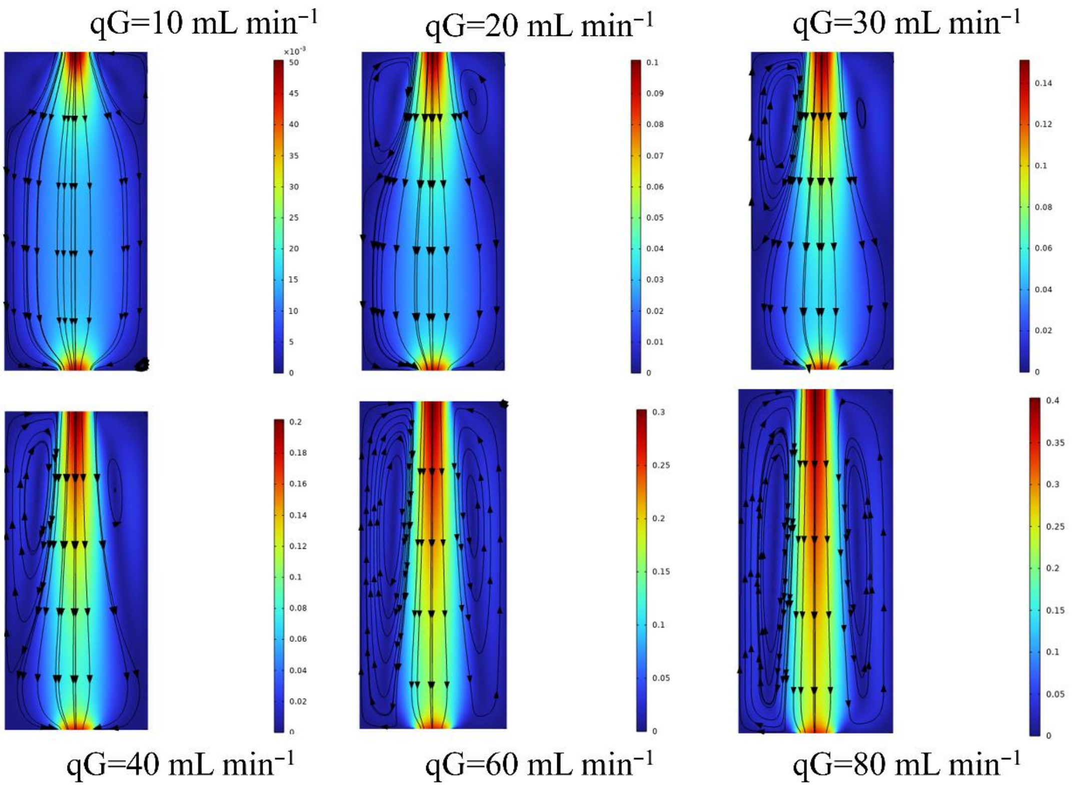

Supplementary Figure 61. Different flow rates download gas flow direction.

It should be noted that Bernoulli principle played a key role in illustrating the interfacial pressure difference at three-phase boundary, which promote oriented Cl_2 migration to gas chamber during reaction process. Theoretically, Bernoulli principle a basic rule in fluid dynamics illustrating the relationship between the surface velocity and pressure. In the present work, two fluids have presented at the three-phase boundary, carrier gas flow and electrolyte flow, and according to Bernoulli principle that can be illustrated as:

$$P_1 + \frac{1}{2}\rho_1 V_1^2 + \rho_1 g h_1 = P_2 + \frac{1}{2}\rho_2 V_2^2 + \rho_2 g h_2 \quad (15)$$

where 1 represents carrier gas flow and 2 represents electrolyte flow. So, the interfacial pressure difference (∇p) can be illustrated as:

$$\nabla p = \frac{1}{2}\rho_1 V_1^2 + \rho_1 g h_1 - \frac{1}{2}\rho_2 V_2^2 - \rho_2 g h_2 \quad (16)$$

Therefore, when high-velocity carrier gas flow and low electrolyte flow have been applied to three-phase boundary, it will generate a large localized pressure gradient. This can promote Cl_2 bubbles migration away from electrolyte flow, and into carrier gas. To clearly clarify this mechanism, we have provided a schematic animation in Supplementary Video 2.

Quantitively, the function of pressure difference has been described by finite element simulations (Figure 5c-d, Supplementary Video 1) and experimental phenomenon of Cl_2 evolution (Figure 3c). Our simulation based on Bernoulli's principle shows the pressure difference in the range of 0 ~ 116.3 mPa with the carrier flow of 0~80 mL min^{-1}, which will drive as-generated Cl_2 migration to gas chamber. This prediction is well aligned with experimental results: by tunning the carrier flow rate from 0 to 80 mL min^{-1}, the Faradaic efficiencies of Cl_2 gas products increases from 17.6% to 82.6% (Figure 3c). Moreover, mechanism study has been conducted by *operando* Raman spectra for chlorine evolution reaction (CER) with/without interfacial pressure difference (Figure 5a and Supplementary Figure 58). The decreased *OCl peak signal unambiguously confirm the role of interfacial pressure difference, *i.e.*, Cl_2 produced at the electrode/electrolyte interfaces intermediately taken away by gas flow following Bernoulli's principle.

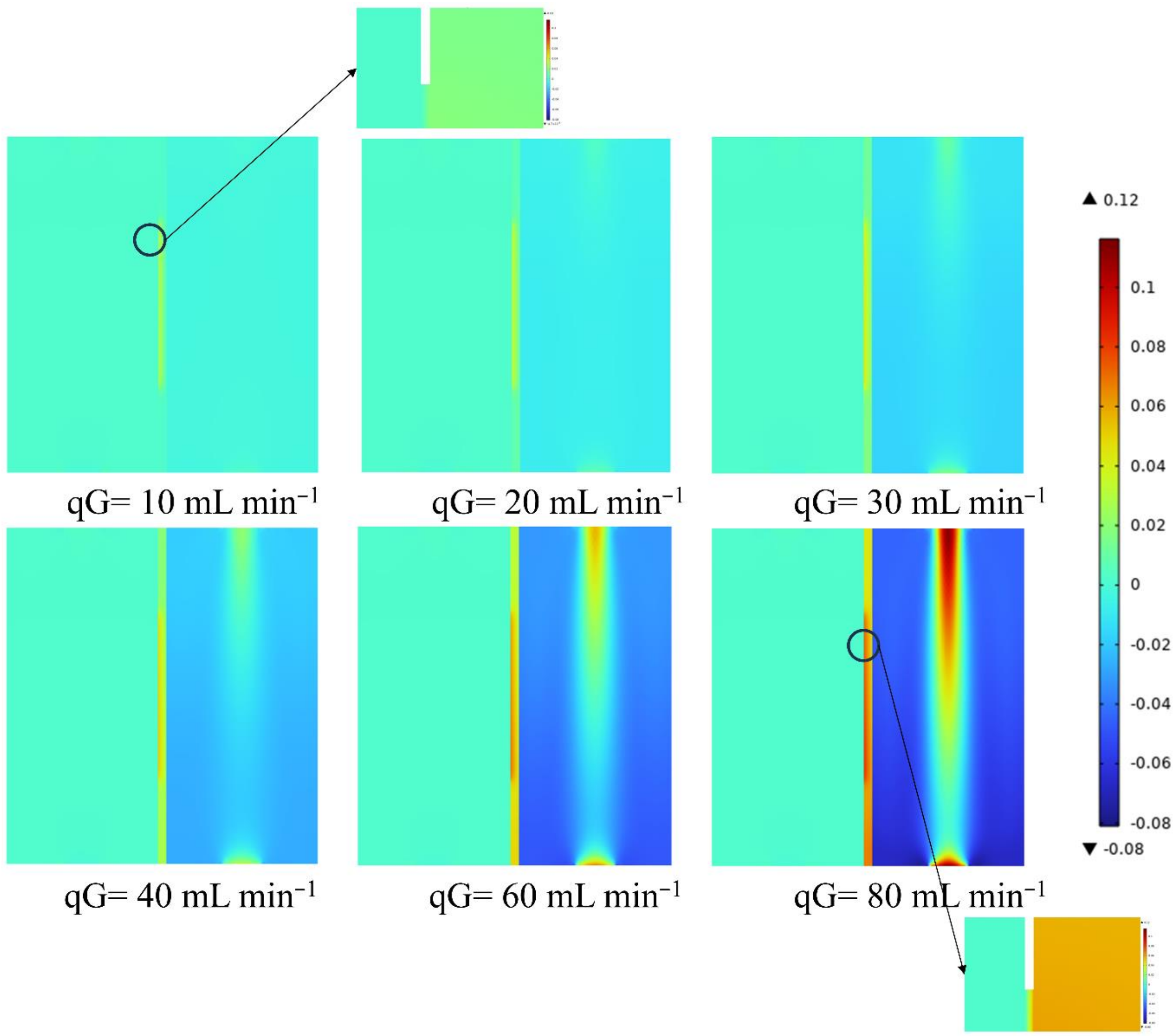

Supplementary Figure 62. Pressure distribution of anode chamber and gas chamber at different carrier gas flow rates.

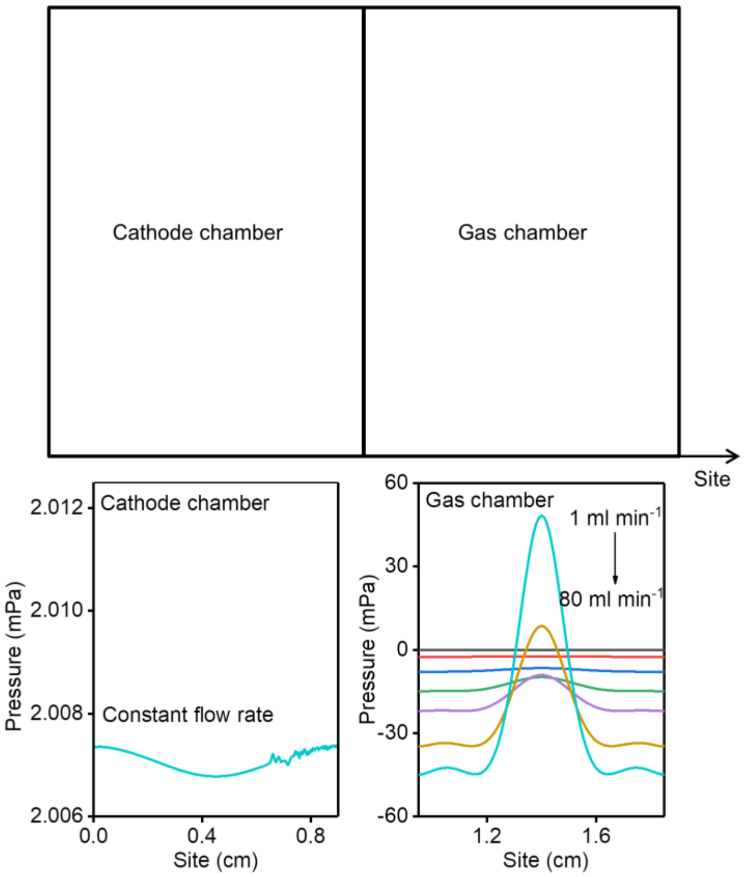

Supplementary Figure 63. Numerical quantization of pressure values at different carrier gas flow rates.

As shown in Supplementary Figures 60-63, the value of interfacial pressure difference is very small (milli-Pa level), and there are many factors influencing the pressure difference, such as gas product evolution (Cl_2 and O_2 byproduct) and interfacial mass transfer (like electrolyte). So, it is very difficult to directly monitor the pressure difference experimentally. Although there is already significant advance in *operando* characterization techniques (like *operando* Raman, FT-IR and XPS). There are seldom reports of *operando* manometer for measure pressures yet. Therefore, we would like to provide some indirect evidence for the presence, stability and function of interfacial pressure difference.

Firstly, the presence/function of interfacial pressure difference can be confirmed by both theoretical simulations and experimental phenomenon of Cl_2 evolution. Theoretically, our simulation based on Bernoulli's principle (Figure 5c-d) shows the pressure difference in the range of 0 ~ 116.3 10^{-3} Pa with the carrier flow of 0~80 mL min^{-1}, which will drive as-generated Cl_2 migration to gas chamber. This prediction is well aligned with experimental results: by tunning the carrier flow rate from 0 to 80 mL min^{-1}, the Faradaic efficiencies of Cl_2 gas products increases from 18.5% to 82.1% (Figure 3c). Moreover, mechanism study has been conducted by *operando* Raman spectra for chlorine evolution reaction (CER) with/without interfacial pressure difference (Figure 5a and Supplementary Figure 58). The decreased *OCl peak signal unambiguously confirm the role of interfacial pressure difference, *i.e.*, Cl_2 produced at the electrode/electrolyte interfaces intermediately taken away by gas flow following Bernoulli's principle.

Further, the stability of interfacial pressure difference can be revealed by stability test of chlorine evolution reaction (CER) with interfacial pressure difference. Ti-MOF electrode exhibits strong stability in universal pH conditions (pH=1, 7, 13) with little morphology and structure decay after stability test after 50 hrs (Figure 2h; Supplementary Figures 38−40). The prototype device shows long-term stability for Cl_2 electrosynthesis with little fluctuation for 200 hrs at a current density of 100 mA cm^{-2} (Figure 3g). Even at extremely high current density of 1.14 A cm^{-2}, our system can still demonstrate excellent stability for 40 hrs (Supplementary Figure 53). All of above results confirm the interfacial pressure difference stable for Cl_2 electrosynthesis.

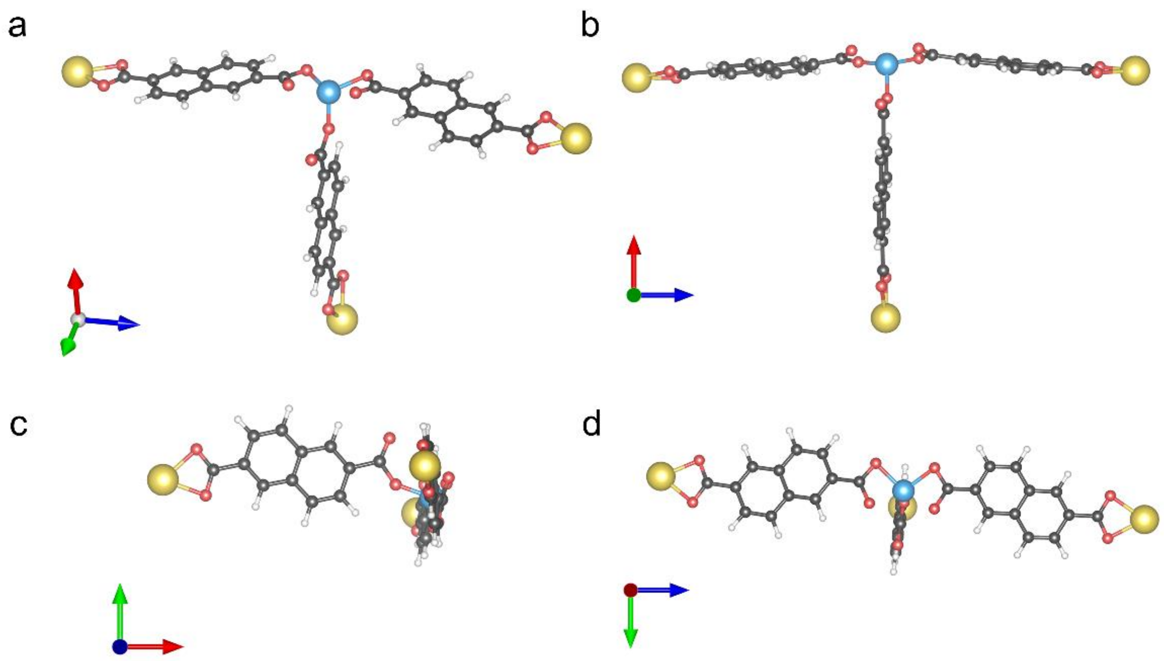

Supplementary Figure 64. a, Three-dimensional perspective view. **b-d,** Observe the modeling structure from three different perspectives.

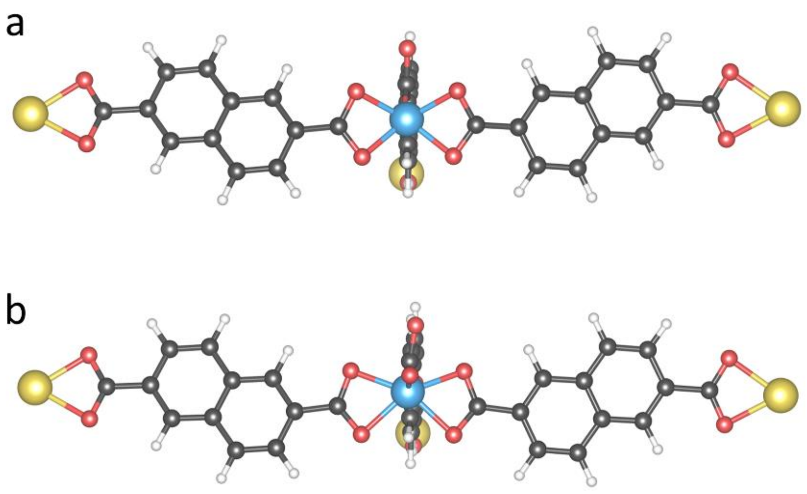

Supplementary Figure 65. Theoretical modeling structures. a, Ti-MOF. **b,** Ti-MOF(O).

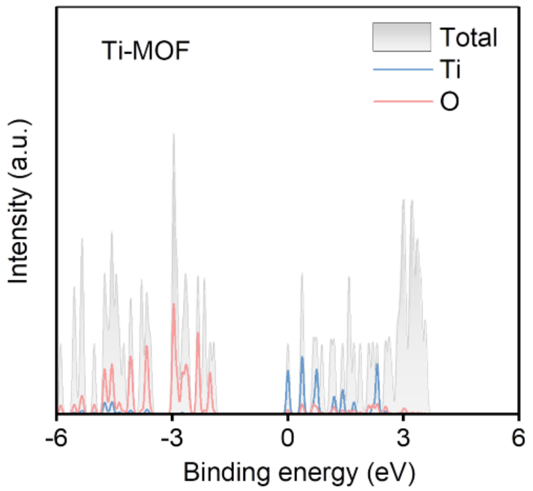

Supplementary Figure 66. Density of states (DOS) profiles of Ti-MOF.

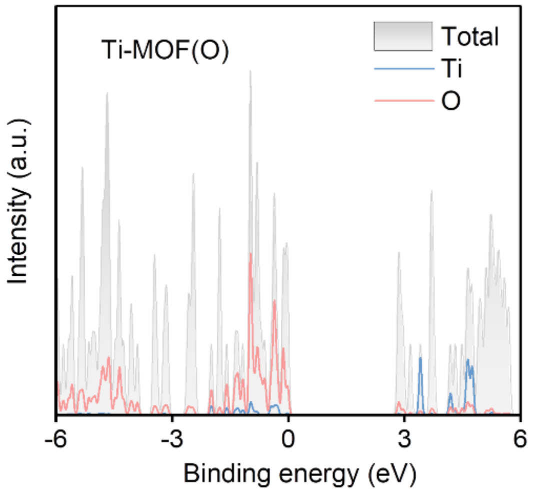

Supplementary Figure 67. Density of states (DOS) profiles of Ti-MOF (O).

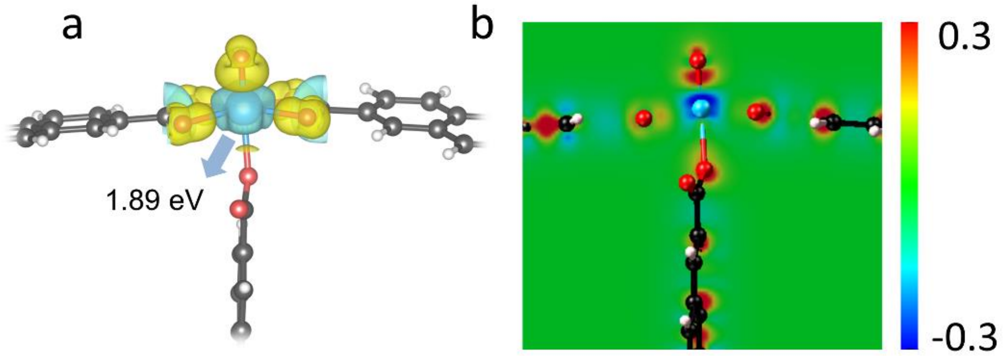

Supplementary Figure 68. Charge transfer profiles of Ti-MOF (O). a, 3D differential charge transfer of Ti-MOF (O). The blue isosurface (1×10^{-2} e/Å^3) represents electron accumulation, and the yellow isosurface (1×10^{-2} e/Å^3) represents electron depletion in 3D differential charge transfer. **b,** 2D differential charge transfer of Ti-MOF (O).

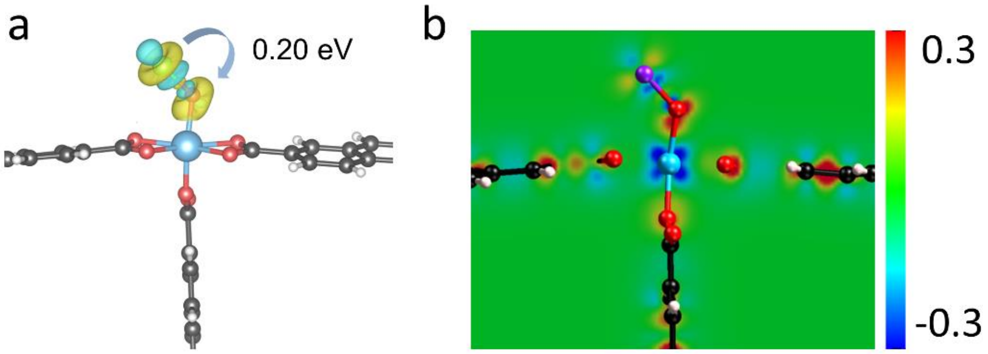

Supplementary Figure 69. Charge transfer profiles of Ti-MOF (O)-*Cl. a, 3D differential charge transfer of Ti-MOF (O)-*Cl. **b,** 2D differential charge transfer of Ti-MOF (O)-*Cl.

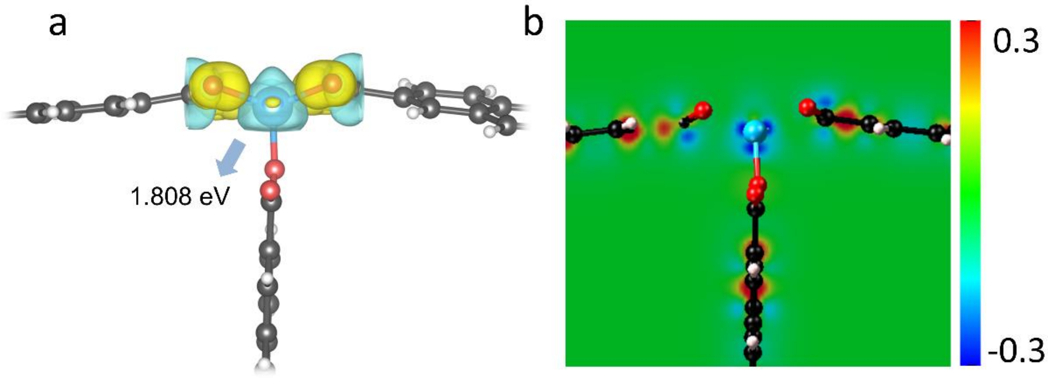

Supplementary Figure 70. Charge transfer profiles of Ti-MOF. a, 3D differential charge transfer of Ti-MOF. **b,** 2D differential charge transfer of Ti-MOF.

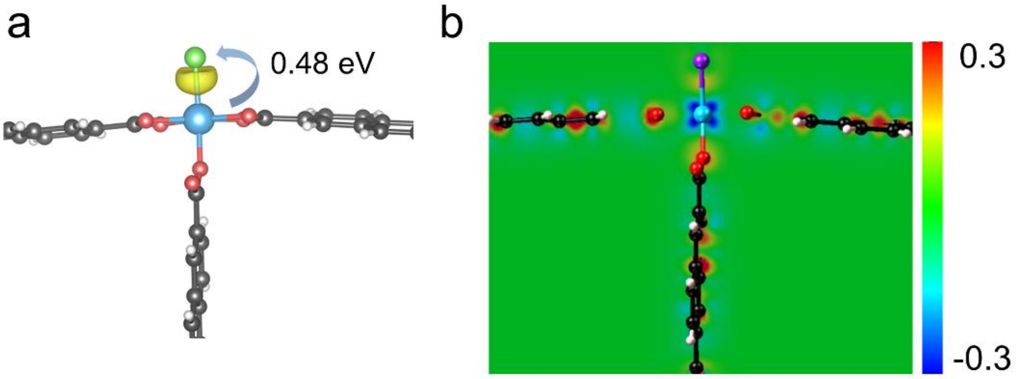

Supplementary Figure 71. Charge transfer profiles of Ti-MOF-*Cl. a, 3D differential charge transfer of Ti-MOF-*Cl. **b,** 2D differential charge transfer of Ti-MOF-*Cl.

The Cl-O bond is covalent in nature that can be formed by adsorbing Cl to oxygen species, as demonstrated in the common chemical of HClO. Inspired by this structure, we have conducted theoretical calculations of charge transfer profiles, where the increase in Ti's electron transfer number indicates that the bonded O acquires electrons (1.89 *vs.* 1.808 eV). Subsequently, the elevated electron density around O enhances its orbital overlap with Cl (Figure 5g), as demonstrated by distinct p-p orbital overlap between O and Cl (green and red lines). This will prompt formation of chemical bonds of Cl-O, and consequently *OCl intermediate during CER process.

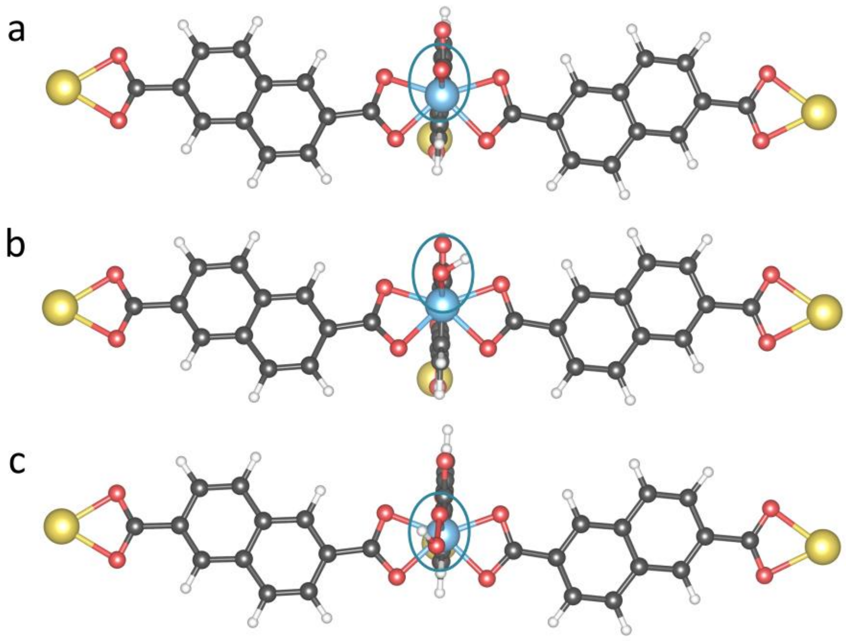

Supplementary Figure 72. Modeling structure demonstration of OER intermediate. a, Ti-MOF-*O. **b,** Ti-MOF-*OH, **c,** Ti-MOF-*OOH.

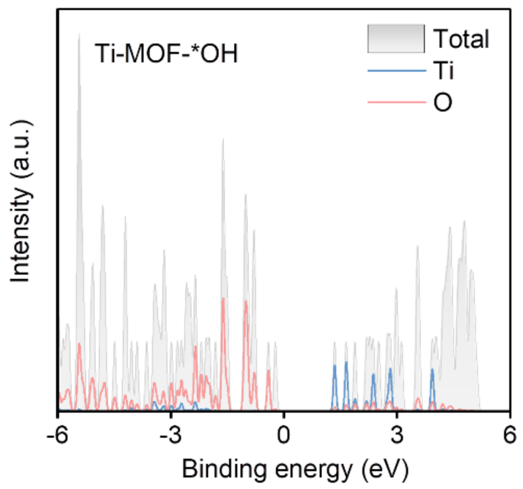

Supplementary Figure 73. Density of states (DOS) profiles of Ti-MOF-*OH.

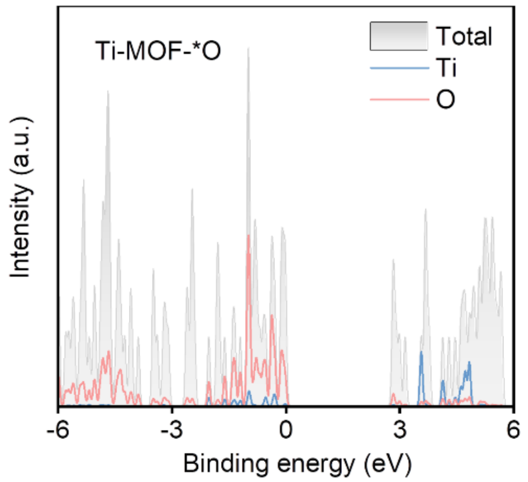

Supplementary Figure 74. Density of states (DOS) profiles of Ti-MOF-*O.

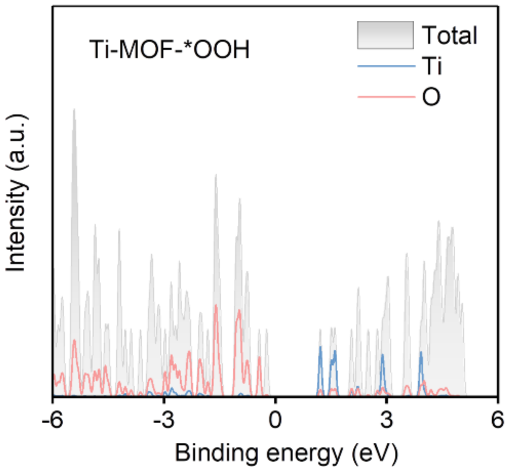

Supplementary Figure 75. Density of states (DOS) profiles of Ti-MOF-*OOH.

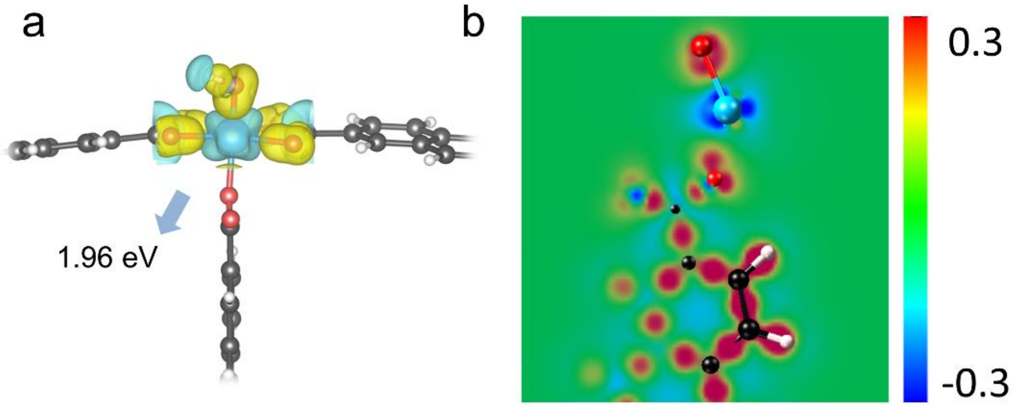

Supplementary Figure 76. Charge transfer profiles of Ti-MOF-*OH. a, 3D differential charge transfer of Ti-MOF-*OH. **b,** 2D differential charge transfer of Ti-MOF-*OH.

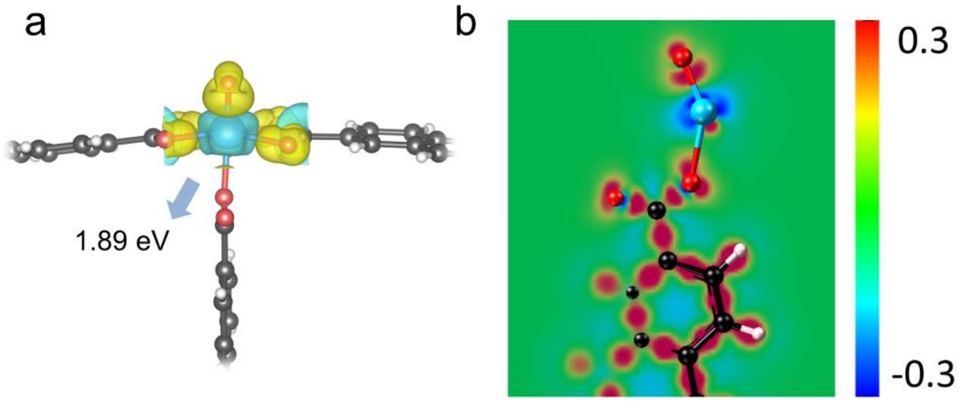

Supplementary Figure 77. Charge transfer profiles of Ti-MOF-*O. a, 3D differential charge transfer of Ti-MOF-*O. **b,** 2D differential charge transfer of Ti-MOF-*O.

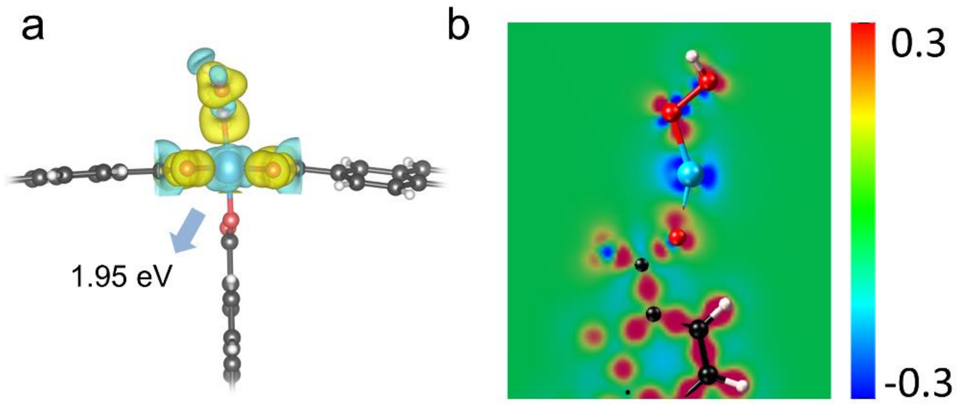

Supplementary Figure 78. Charge transfer profiles of Ti-MOF-*OOH. a, 3D differential charge transfer of Ti-MOF-*OOH. **b,** 2D differential charge transfer of Ti-MOF-*OOH.

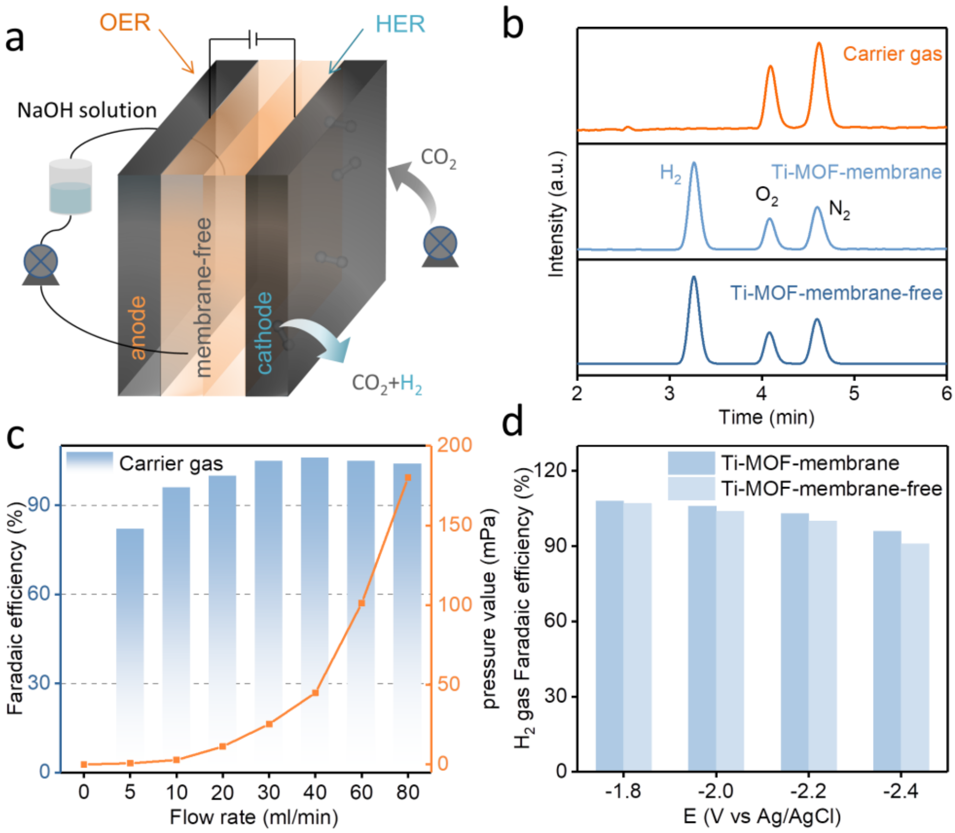

Supplementary Figure 79. Membrane-free water splitting (in 1 M KOH electrolyte) for hydrogen production. a, Schematic diagram of the device. **b,** Gas phase data for carrier gas and reaction products. **c,** The relationship between carrier gas flow rate and pressure (hydrogen Faraday efficiency). **d,** Hydrogen selectivity tested by Ti-MOF in the presence and absence of membrane at different potentials.

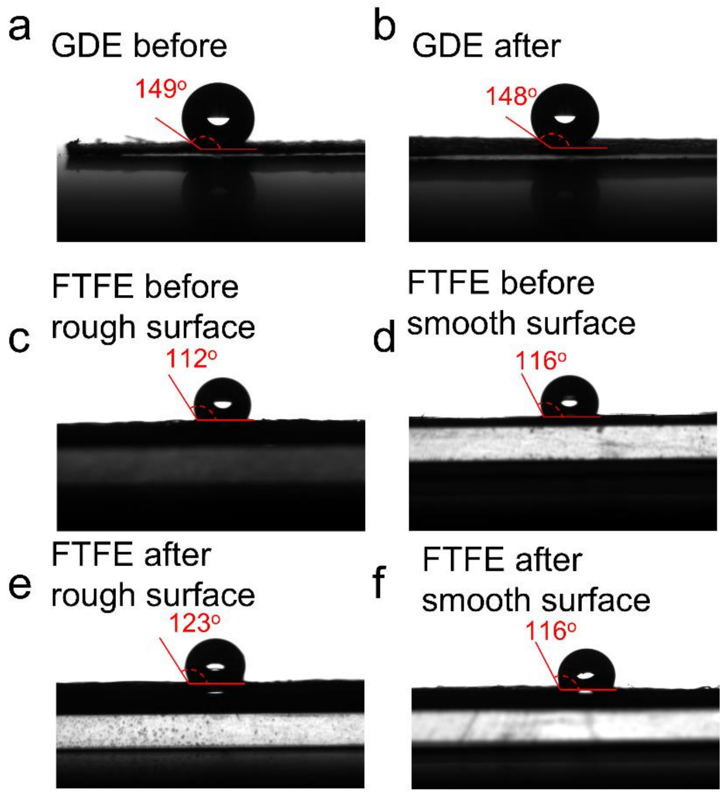

Supplementary Figure 80. Contact Angle test. a-b, GDE before and after testing. **c-d,** rough and smooth PTFE surfaces before testing. **e-f,** rough and smooth PTFE surfaces after testing.

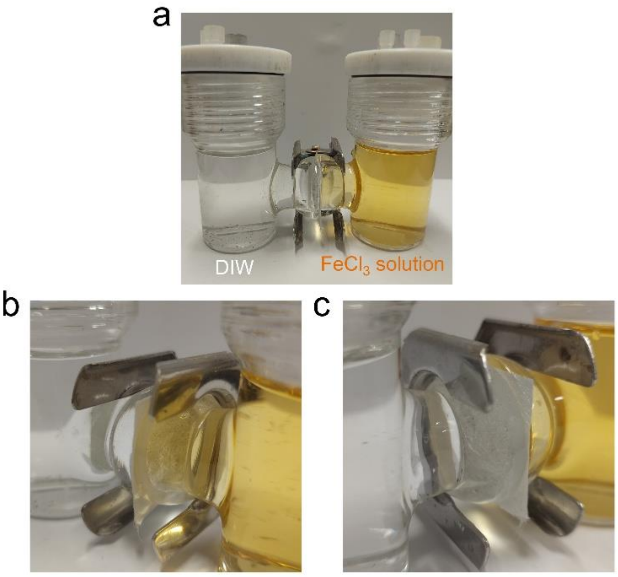

Supplementary Figure 81. Test of PTFE water resistance in H-cell. a-c, observe the water resistance of PTFE from different perspectives.

In terms of the water leakage concern, we consider the multilayered waterproofing design of GDE/PTFE robustly preventing aqueous phase penetration into the gas chamber. More specifically, the gas diffusion electrode (GDE) maintained near-constant hydrophobicity on its gas-facing side (149° pre-reaction *vs* 148° post-reaction; Supplementary Figures 80a-b), confirming its effectiveness as the barrier against electrolyte penetration. Secondly, the hydrophobicity has been further reinforced by the PTFE partition layer (Supplementary Figure 41a), which exhibits persistent hydrophobicity on both interfaces: GDE-facing side (152°→150°) and gas chamber side (153°→151°) as shown in Supplementary Figures 80c-f. As a consequence, the minimal variations of contact angle ($\Delta\theta \leq 2°$) demonstrate robust waterproof integrity throughout operation.

To further verify above hypothesis, we have conducted long-term barrier integrity and mass transfer resistance of PTFE membrane in H-cell experiment (Supplementary Figure 81). Deionized water (DIW) and yellow $FeCl_3$ solution were physically separated by the PTFE membrane in adjacent chambers. We can see that no color change in the DIW sample after one week, which reveals the effective liquid isolation function of PTFE.

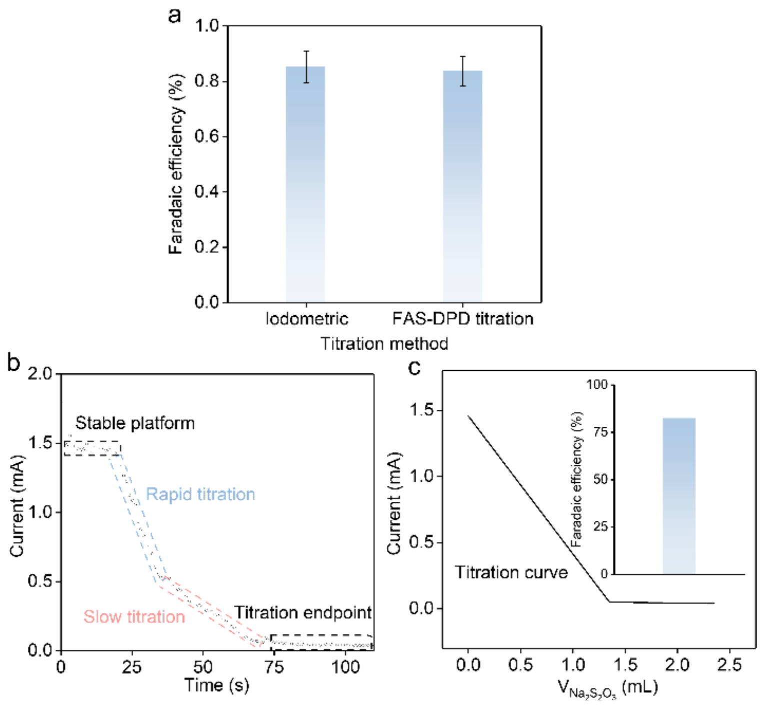

Supplementary Figure 82. Error determination. a, The Faraday efficiency of Cl_2 at 1.8 V and the errors observed in repeated tests using the KI titration method and the FAS-DPD titration method were represented as error bars. **b,** The measured current-time relationship of the ampere titration method. **c,** The typical I_2-$Na_2S_2O_3$ titration curve and the calculated Faraday efficiency of the Cl2 method.

The specific details of the FAS-DPD titration method: A DPD indicator is added to deionized water, which reacts with chlorine to show a pink color. Then add FAS drop by drop until the pink color completely disappears, which marks the arrival of the endpoint. The number of drops used corresponds to the concentration of chlorine. Its reaction equation is

$$Cl_2 + H_2O = HCl + HClO \tag{17}$$

$$HClO + DPD \rightarrow Cl^- + H_2O + 2DPD^+ \tag{18}$$

$$DPD^+ + (NH_4)_2Fe(SO_4)_2 \rightarrow DPD + NH_4Fe(SO_4)_2 + NH_4^+ \tag{19}$$

Based on the above equation, the formula for calculating the production of Cl_2 can be derived:

$$c_{Cl_2} = \frac{c_{FAS} \times V_{FAS} \times 1000}{2 \times V_{sample}} \tag{20}$$

In the formula, c_{FAS} represents the concentration of the FAS, mol L^{-1}; V_{FAS} is the volume of the FAS taken, L; V_{sapmle} is the volume of the absorbent solution, L.

We have quantitatively titrated five times by using FAS-DPD titration method (Faradaic efficiency: 83.7 ± 5.3%, Supplementary Figure 82a), which is close to the result of direct KI-thiosulfate titration method (Faradaic efficiency: 85.2 ± 5.6%).. Notably, the results are comparable for KI-thiosulfate titration method and FAS-DPD titration method, thereby confirming the reliability of our work (Supplementary Figure 82a).

When using the amperometric method to determine Cl_2 efficiency, the separated Cl_2 is first absorbed by KI solution. The entire absorption solution is then transferred to a single-compartment electrochemical cell. Two clean Pt electrodes are used as redox electrodes, with the working electrode and counter electrode clamped to the two Pt electrodes, respectively. A potential of E=0.3 V is applied (Supplementary Figure 82b). In the first stage, the stable platform period, where no $Na_2S_2O_3$ is added, the current is observed to stabilize at around 1.4 mA. When continuous dripping of $Na_2S_2O_3$ begins (the rapid titration stage), the current drops sharply. Once the current decreases to 0.5 mA, the titration speed is reduced (entering the slow titration stage), and the current declines slowly. Titration is stopped when the current drops to approximately 0.04 mA. After waiting for half a minute, the current shows almost no change, and the yellow color of I_2 in the cell fades without returning. The consumption of $Na_2S_2O_3$ at the endpoint is recorded and plotted as a typical amperometric I_2-$Na_2S_2O_3$ titration curve (Supplementary Figure 82c). The Faraday efficiency of Cl_2 is calculated to be 83±2.5% based on the formula, which is close to the result obtained from direct titration.

Supplementary Table 1. The crystal plane indices of Ti-MOF and the simulated value.

(h k l) (Simulation)	2θ (Simulation)	2θ (Experiment)
(1 0 0)	7.434	7.49
(1 0 1)	10.826	10.6
(2 0 1)	14.665	15
(2 1 0)	16.171	16
(0 −1 1)	19.759	19.3
(0 1 2)	25.79	25.9
(3 2 0)	29.104	29.5
(4 2 0)	32.676	32.5
(0 2 3)	43.289	43.3

Supplementary Table 2. Bond length and coordination of Ti-MOF-before and after calcination.

Material	shell	CN	R (Å)	ΔE_0（eV）	σ^2（10^{-3} Å^2）	R factor
Ti-foil	Ti-Ti	6.24±0.21	2.89±0.002	−7.05±0.18	0.006±0.0005	0.0084±0.00224
TiO_2	Ti-C	4.15±0.05	1.95±0.003	−1.55±0.26	0.006±0.0001	0.014±0.0012
	Ti-Ti	2.05±0.05	3.06±0.0004		0.0040±0.00025	
TiC	Ti-O	6.03±0.08	2.14±0.00039	−3.47±0.01	0.0002±0.0001	0.0095±0.00035
	Ti-Ti	11.93±0.14	3.05±0.0002		0.0009±0.0001	
Ti-MOF (before)	Ti-O	3.98±0.04	1.90±0.0005	−7.04±0.10	0.016±0.0001	$0.0034\pm4.1\times10^{-5}$
	Ti-Ti	4.05±0.002	3.10 ± 10^{-7}		$0.019\pm1.5\times10^{-5}$	
Ti-MOF	Ti-O	3.36±0.11	1.92±0.002	−4.21±0.21	0.011±0.0009	0.013±0.0043
	Ti-Ti	4.05±0.09	3.12±0.00008		0.018±0.00064	

Supplementary note: CN: coordination number; R: bond lengths between central atoms and surrounding coordination atoms; ΔE_0: the difference of the zero kinetic energy value between the sample and theoretical model; σ^2: Debye-Waller factor to account for both thermal and structural disorders; R factor is used to measure the goodness of the fitting. All the data in the figure are the average results after five repeated fittings, and the standard deviation of each average value is added to indicate the relative deviation of the value.

Supplementary Table 3. The comparison of the overpotential (10 mA cm^{-2}) of CER.

Electrocatalysts	Overpotential (mV)	Reaction conditions	References
RuO_x/2D TiO_x	90	1 M pH=1	[26]
Ru-O_4	30	1 M pH=1	[27]
CoO_xCl_y	100	0.5 M pH=2	[28]
RCON-H	89 (1 A cm^{-2})	5 M pH=2	[5]
MoO_x@IrO_2-Ta_2O_5	30	4 M pH=2	[29]
Pt_1/p-NC@CNTs	40	1M 0.1 $HClO_4$	[30]
Ir_1O_6	71.8	4 M pH=2	[25]
RuO_2/TiO_2	220	4 M pH=3	[31]
Ni(Co/Mn)Sb_2O_x	450	4 M pH=2	[19]
Co_3O_4/FTO	200	Saturated pH=3	[32]
$Ti_{0.35}V_{0.35}Sn_{0.25}Sb_{0.05}$-oxide	987	5 M NaCl pH≈2	[20]
Ti-MOF	148	5 M NaCl pH=1	This work
	156	5 M NaCl pH=7	
	404	5 M NaCl pH=13	

Supplementary Table 4. The pressure difference generated by air at different flow rates as carrier gas and the magnitude of the driving force produced by this pressure difference.

Air flow velocity/mL min^{-1}	Pressure/10^{-3} Pa	Generate pushing weight/mg cm^{-2}
0	0	0
10	1.817141784	0.017807989
20	7.268567137	0.071231958
30	16.35427606	0.160271905
40	29.07426855	0.284927832
60	65.41710423	0.641087621
80	116.2970742	1.139711327

Supplementary Table 5. The pressure difference generated by CO_2 at different flow rates as carrier gas and the magnitude of the driving force produced by this pressure difference.

CO_2 flow velocity/mL min^{-1}	Pressure/10^{-3} Pa	Generate pushing weight/mg cm^{-2}
0	0	0
10	2.813048173	0.027567872
20	11.25219269	0.110271488
30	25.31743356	0.248110849
40	45.00877	0.441085954
60	101.2697	0.992443395
80	180.0351	1.764343814

In Figure 3c, the plateau in Cl_2 Faradic efficiency beyond 40 mL min^{-1} gas flow stems from kinetic bottlenecks, *i.e.*, the competition between Cl_2 dissolution in aqueous electrolyte (driven by gas-liquid equilibrium) and Cl_2 migration to gas chamber (driven by Bernoulli's principle).

Firstly, Cl_2 is a soluble gas in water (0.79 g per100 g of water), and according to Pourbaix diagram

(Figure 1c), rapidly hydrolyzes to produce hypochlorous acid (HClO) and hypochlorous acid which then dissociates into hypochlorite ion (OCl^-) and hydrogen ion (H^+):

$$Cl_2 + H_2O \rightarrow HCl + HClO \tag{21}$$

$$HClO \leftrightarrow ClO^- + H^+ \tag{22}$$

We have calculated the mass of Cl_2 theoretically produced per second with Faradaic efficiency of Cl_2 generation assumed to be 100% at 100 mA cm^{-2}:

$$m = \frac{I \times T \times M}{n \times F} \tag{23}$$

where m represents the mass of Cl_2, I is the current density, T is time, M is the molar mass of Cl_2 (71 g mol^{-1}), n is the number of transferred electrons, and F is the Faraday constant (96485 C mol^{-1}). Based on above formula, it is estimated Cl_2 generation rate as 0.0000368 g cm^{-2} s^{-1}. By comparison with the Cl_2 solubility in water (0.79 g per100 g of water), most of the produced Cl_2 would be dissolved and dissociated into OCl^-/ Cl^- couple driven by gas-liquid equilibrium.

On the other hand, we have calculated the driven force of Cl_2 migration to gas chamber according to Bernoulli's principle (Supplementary Table 4). By calibration into electrode area, the generate pushing weight of Cl_2 is in the range of 0~0.16 mg cm^{-2} for 0~30 mL min^{-1}, and 0.28~1.14 mg cm^{-2} for 40~80 mL min^{-1}. By dividing Cl_2 theoretical productivity (0.0000368 g cm^{-2} s^{-1}), it can be concluded that the at least seven folds of Cl_2 migration driven force can overcome the Cl_2 dissolution in aqueous electrolyte driven by gas-liquid equilibrium.

Supplementary Table 6. The comparison of the performance of CER.

Catalyst	System configuration	FE (%)	Current density (mA cm^{-2})	Yield rate (mmol h^{-1} cm^{-2})	Reference
Ti-MOF	Membrane-free	87.6	1138.9	18.675	This work
NCOOH	Membrane-based	99.6	1000	18.562	[5]
Ru-O_4 SAM	Membrane-based	98	1000	18.283	[27]
Ti/RuO_2–Sb_2O_5–SnO_2	Membrane-based	82.5	1000	15.391	[33]
RuO_2-TiO_2 NBs-Ti	Membrane-based	95	300	5.317	[34]
Pt_2/F-CNTs	Membrane-based	98	200	3.657	[1]
Co_3O_4 NPs	Membrane-based	80	20	0.298	[35]
CoO_xCl_y	Membrane-based	100	250	4.664	[28]
RuO_2@TiO_2	Membrane-based	90	250	4.198	[36]
RuO_2/Nb:TiO_2NPs	Membrane-based	97.7	50	0.911	[17]
RuO_2@TP	Membrane-based	96.5	100	1.800	[37]
Ti-mesh electrode	Redox mediator -based	47.4	-	8.85	Ref 14 in manuscript
RuO_2/IrO_2-coated Ti-mesh	Redox mediator -based	45.1	-	1.68	Ref 6 in manuscript

Supplementary Table 7. The prices of various raw materials.

Item	Producer/References	Price ($ Unit price)	Price ($ total)
2,6 naphthoic acid	Reagent Suppliers: Bide Pharmatech	$438.36 kg^{-1}	$4109.59
NaOH	Industrial supplier: Dezhou Zhongzhiyuan Water Purification Materials	$452 ton^{-1}	$1.57
N, N-Dimethylformamide	Industrial supplier: Shandong Jinshengrun Chemical Industry	$500 ton^{-1}	$52.5
methanol	Industrial supplier: Shandong Mingshui Dahua Co., LTD	$ 332.7 ton^{-1}	$3.74
Tetraisopropyl titanate	Reagent Suppliers: MERYER	$42.6 kg^{-1}	$184.2
GDE	Industrial supplier: Shanghai Jiazhi Materials	$94.52 m^{-2}	$47415.95
DSA	[38]	$4800 m^{-2}	$2400000
water	https://www.fbgtx.org/673/Industrial-Water-Rates	$0.409/ton	/
electricity	[6,7,8,9]	$0.03 kWh	/
membrane	Industrial supplier: Asahi Kasei Singapore Branch	$700 m^{-2}	$350000
NaCl	[39]	$0.0928 kg^{-1}	/

Supplementary Table 8. CER simulation electrolysis part data of Ti-MOF.

Current density (A cm^{-2})	0.18	0.34	0.7	1.14
Potential (V)	1.8	2	2.2	2.4
Materials	Ti-MOF	Ti-MOF	Ti-MOF	Ti-MOF
FE (%)	0.87	0.79	0.74	0.7
Electrolytic area (m^2)	500	500	500	500
Current (A)	900000	1700000	3500000	5700000
Cl_2 production (kg h^{-1})	1037.1291	1778.881691	3430.605794	5284.99
NaOH production (kg h^{-1})	1168.5962	2004.373737	3865.471317	5954.92
H2O consumption (kg h^{-1})	302.22314	570.8659377	1175.312225	1914.08
NaCl consumption (kg h^{-1})	1709.0719	2931.39659	5653.251801	8709.06
Electrolytic power (kW)	1620	3400	7700	13680
Separation power (kW)	4652	9304	13956	20934

Supplementary Table 9. FNPV ($) analysis of Ti-MOF-membrane-free for CER at different current densities.

J (A cm^{-2}) / Time (year)	1.14	0.7	0.34	0.18
0	−11941000	−11941000	−11941000	−11941000
1	−5827126.4	−8397349.173	−10306896.35	−10946665.68
2	−427552.84	−5445606.889	−9173770.417	−10422843.87
3	4697891.33	−2651430.564	−8111609.669	−9940972.77
4	9562259.94	−7317.05775	−7117034.806	−9499054.71
5	14177985.1	2493879.479	−6186827.454	−9095187.171
6	18556907.1	4858964.616	−5317922.493	−8727558.222
7	22710302.2	7094419.849	−4507400.761	−8394442.216
8	26648909.7	9206418.03	−3752482.105	−8094195.679
9	30382957.7	11200838.07	−3050518.76	−7825253.4
10	33922187.1	13083278.92	−2398989.042	−7586124.698
11	37275874.9	14859072.92	−1795491.353	−7375389.881
12	40452856.6	16533298.51	−1237738.451	−7191696.857
13	43461546.6	18110792.26	−723552.0135	−7033757.923
14	46309958.8	19596160.47	−250857.4475	−6900346.693
15	49005725.5	20993790.05	182321.0507	−6790295.181
16	51556115.6	22307859.03	577865.1986	−6702491.021
17	53968051.8	23542346.51	937567.1083	−6635874.813
18	56248127	24701042.06	1263133.553	−6589437.609
19	58402620.5	25787554.83	1556190.031	−6562218.503
20	60437512.2	26805322.09	1818284.635	−6553302.348

Supplementary Table 10. FNPV ($) analysis of Ti-MOF-membrane for CER at different current densities.

Time (year) \ J (A cm^{-2})	1.14	0.7	0.34	0.18
0	−11941000	−11941000	−11941000	−11941000
1	−6664621.225	−9234844.03	−11144391.2	−11784160.54
2	−2062661.848	−7080715.896	−10808879.42	−12057952.88
3	2303149.805	−5046172.095	−10506351.2	−12335714.3
4	6444058.861	−3125518.134	−10235235.88	−12617255.79
5	10370774.97	−1313330.689	−9994037.622	−12902397.34
6	14093497.79	395555.3135	−9781331.796	−13190967.52
7	17621941.3	2006058.989	−9595761.621	−13482803.08
8	20965356.88	3522865.211	−9436034.924	−13777748.5
9	24132555.4	4950435.762	−9300921.063	−14075655.7
10	27131928.13	6293019.961	−9189247.998	−14376383.65
11	29971466.79	7554664.776	−9099899.501	−14679798.03
12	32658782.52	8739224.464	−9031812.497	−14985770.9
13	35201124.04	9850369.745	−8983974.534	−15294180.44
14	37605394.86	10891596.54	−8955421.372	−15604910.62
15	39878169.8	11866234.31	−8945234.686	−15917850.92
16	42025710.56	12777453.95	−8952539.884	−16232896.1
17	44053980.67	13628275.38	−8976504.018	−16549945.94
18	45968659.68	14421574.7	−9016333.806	−16868904.97
19	47775156.7	15160091.06	−9071273.74	−17189682.27
20	49478623.25	15846433.17	−9140604.289	−17512191.27

Supplementary Table 11. CER simulation electrolysis and FNPV ($) analysis of DSA-membrane for CER at 1 A cm^{-2}.

Current density (A cm^{-2})	1.14	J (A cm^{-2}) / Time (year)	1.14
Potential (V)	1.42	0	-22908800
Materials	DSA	1	-15127547.82
FE (%)	0.9	2	-8513049.138
Electrolytic area (m^2)	500	3	-2230533.39
Current (A)	5700000	4	3735808.139
Cl_2 production (kg h^{-1})	6589.6772	5	9401031.364
NaOH production (kg h^{-1})	7655.7665	6	14779475.25
H_2O consumption (kg h^{-1})	3639.839025	7	19884795.96
NaCl consumption (kg h^{-1})	11807.81997	8	24729999.36
Electrolytic power (kW)	8094	9	29327471.98
Separation power (kW)	29075	10	33689010.53
/	/	11	37825849.97
/	/	12	41748690.25
/	/	13	45467721.81
/	/	14	48992649.83
/	/	15	52332717.32
/	/	16	55496727.19
/	/	17	58493063.11
/	/	18	61329709.57
/	/	19	64014270.82
/	/	20	66553989.02

The incorporation of DSA significantly elevates NaOH/Cl_2 production costs from traditional membrane system, which underperform Ti-MOF-based membrane-free system in the range of 1~10

year's operation. After 11 tears, traditional DSA-membrane system begins to gain advantage due to its lower power consumption. Overall, the production cost per kg of Cl_2/NaOH are comparable for DSA-based membrane system and our Ti-MOF-based membrane-free system ($0.36768 *vs* $0.36069).

Supplementary Table 12. Frequency (f), vibrational wave number (v) and entropy of *OCl adsored on Ti-MOF.

Ti-MOF-*OCl	f THz	v cm^{-1}	entropy $J\cdot mol^{-1}\cdot K^{-1}$
γ point 1	12.400054	413.621268	0.011815283
γ point 2	2.834387	94.544967	0.046073464
γ point 3	1.757446	58.622082	0.058216775

Supplementary Table 13. Frequency (f), vibrational wave number (v) and entropy of *Cl adsored on Ti-MOF.

Ti-MOF-*Cl	f THz	v cm^{-1}	entropy $J\cdot mol^{-1}\cdot K^{-1}$
γ point 1	9.008457	300.489785	0.018282827
γ point 2	3.684842	122.91309	0.039483684
γ point 3	2.080592	69.401087	0.053914559

Supplementary Table 14. Frequency (f), vibrational wave number (v) and entropy of *OH adsored on Ti-MOF.

Ti-MOF-*OH	f THz	v cm^{-1}	entropy $J \cdot mol^{-1} \cdot K^{-1}$
γ point 1	112.392648	3749.015203	6.8162E−09
γ point 2	18.214914	607.584128	0.005646998
γ point 3	17.304733	577.223751	0.006341717
γ point 4	9.439334	314.862307	0.017280621
γ point 5	6.393711	213.27124	0.026056894
γ point 6	3.40717	113.650963	0.041442737

Supplementary Table 15. Frequency (f), vibrational wave number (v) and entropy of *O adsored on Ti-MOF.

Ti-MOF-*O	f THz	v cm^{-1}	entropy $J{\cdot}mol^{-1}{\cdot}K^{-1}$
γ point 1	24.851245	828.948295	0.002391744
γ point 2	5.837739	194.726023	0.028214256
γ point 3	3.999466	133.407837	0.037444507

Supplementary Table 16. Frequency (f), vibrational wave number (v) and entropy of *OOH adsored on Ti-MOF.

Ti-MOF-*OOH	f THz	v cm^{-1}	entropy $J\cdot mol^{-1}\cdot K^{-1}$
γ point 1	108.843791	3630.638083	1.17069E−08
γ point 2	39.461594	1316.297088	0.000329792
γ point 3	27.475096	916.470548	0.001690598
γ point 4	14.426695	481.222742	0.00913736
γ point 5	5.387763	179.71642	0.030140481
γ point 6	3.560783	118.77492	0.040339025
γ point 7	2.196693	73.273795	0.052533204

Reference

1. Shao, X. et al. Coordination Environment and Distance Optimization of Dual Single Atoms on Fluorine - Doped Carbon Nanotubes for Chlorine Evolution Reaction. *Angew. Chem., Int. Ed.* **63**, e202406273 (2024).

2. Sumaria, V., Krishnamurthy, D.,Viswanathan, V. Quantifying Confidence in DFT Predicted Surface Pourbaix Diagrams and Associated Reaction Pathways for Chlorine Evolution. *ACS Catal.* **8**, 9034−9042 (2018).

3. Exner, K. S., Anton, J., Jacob, T.,Over, H. Chlorine Evolution Reaction on RuO_2(110): Ab initio Atomistic Thermodynamics Study-Pourbaix Diagrams. *Electrochim. Acta* **120**, 460−466 (2014).

4. Gao, G., O'Mullane, A. P.,Du, A. 2D MXenes: A New Family of Promising Catalysts for the Hydrogen Evolution Reaction. *ACS Catal.* **7**, 494−500 (2016).

5. Yang, J. et al. CO_2-mediated organocatalytic chlorine evolution under industrial conditions. *Nature* **617**, 519−523 (2023).

6. Chen, F.-Y. et al. Electrochemical nitrate reduction to ammonia with cation shuttling in a solid electrolyte reactor. *Nat. Catal.* **7**, 1032−1043 (2024).

7. Zhang, L. et al. High-efficiency ammonia electrosynthesis from nitrate on ruthenium-induced trivalent cobalt sites. *Energy Environ. Sci.* **18**, 5622−5631 (2025).

8. Gao, Y. et al. Membrane-Free Electrosynthesis of Epichlorohydrins Mediated by Bromine Radicals over Nanotips. *J. Am. Chem. Soc.* **146**, 714−722 (2023).

9. Cheng, C. et al. Selective electrosynthesis of 1,3-butadiene by tailoring the coverage of acetylene and water. *Nat. Commun.* **16**, 5685 (2025).

10. Chu, S., Cui, Y.,Liu, N. The path towards sustainable energy. *Nat. Mater.* **16**, 16−22 (2017).

11. Huang, Z., Grim, R. G., Schaidle, J. A.,Tao, L. The economic outlook for converting CO_2 and electrons to molecules. *Energy Environ. Sci.* **14**, 3664−3678 (2021).

12. Feng, X. et al. Rational Construction of an Artificial Binuclear Copper Monooxygenase in a Metal-Organic Framework. *J. Am. Chem. Soc.* **143**, 1107−1118 (2021).

13. Dan-Hardi, M. et al. A New Photoactive Crystalline Highly Porous Titanium(IV) Dicarboxylate. *J. Am. Chem. Soc.* **131**, 10857−10859 (2009).

14. Huang, Q. et al. Single-zinc vacancy unlocks high-rate H_2O_2 electrosynthesis from mixed dioxygen beyond Le Chatelier principle. *Nat. Commun.* **15**, 4157 (2024).

15. Stern, E. A.Kim, K. Thickness effect on the extended-x-ray-absorption-fine-structure amplitude. *Phys. Rev. B* **23**, 3781-3787 (1981).

16. Näslund, L.-Å.Magnuson, M. The origin of Ti 1s XANES main edge shifts and EXAFS oscillations in the energy storage materials Ti_2CT_x and $Ti_3C_2T_x$ MXenes. *2D Mater* **10**, 035024 (2023).

17. Lim, H. W. et al. Rational Design of Dimensionally Stable Anodes for Active Chlorine Generation. *ACS Catal.* **11**, 12423−12432 (2021).

18. Karlsson, R. K. B.Cornell, A. Selectivity between Oxygen and Chlorine Evolution in the Chlor-Alkali and Chlorate Processes. *Chem. Rev.* **116**, 2982−3028 (2016).

19. Moreno-Hernandez, I. A., Brunschwig, B. S.,Lewis, N. S. Crystalline nickel, cobalt, and manganese antimonates as electrocatalysts for the chlorine evolution reaction. *Energy Environ. Sci.* **12**, 1241−1248 (2019).

20. Mirzaei Alavijeh, M. et al. A selective and efficient precious metal-free electrocatalyst for chlorine evolution reaction: An experimental and computational study. *Chem. Eng. J.* **421**,

127785 (2021).

21. Rasmi, K. R., Vanithakumari, S. C., George, R. P., Mallika, C.,Kamachi Mudali, U. Development and performance evaluation of nano platinum coated titanium electrode for application in nitric acid medium. *Mater. Chem. Phys.* **151**, 133−139 (2015).

22. Pirkarami, A., Javanmard, A.,Ghasemi, E. A highly-stable and low-energy-barrier photoelectrocatalyst (NiFe-LDH@Ti-MOF) for water splitting at high current densities via breaking the atomic structure symmetry. *J. Ind. Eng. Chem.* **137**, 606−618 (2024).

23. Wang, S. et al. Toward a Rational Design of Titanium Metal-Organic Frameworks. *Matter* **2**, 440−450 (2020).

24. Quan, L., Zhao, X., Yang, L. M., You, B.,Xia, B. Y. Intrinsic Activity Identification of Noble Metal Single‐Sites for Electrocatalytic Chlorine Evolution. *Angew. Chem., Int. Ed.* **64**, 202414202 (2024).

25. Wang, J. et al. Engineering the Coordination Environment of Ir Single Atoms with Surface Titanium Oxide Amorphization for Superior Chlorine Evolution Reaction. *J. Am. Chem. Soc.* **146**, 11152−11163 (2024).

26. Ji, J. et al. Ruthenium Oxide Clusters Immobilized in Cationic Vacancies of 2D Titanium Oxide for Chlorine Evolution Reaction. *Small Struct.* **5**, 2300240 (2023).

27. Liu, Y. et al. Electrosynthesis of chlorine from seawater-like solution through single-atom catalysts. *Nat. Commun.* **14**, 2475 (2023).

28. Xiao, M. et al. Self-adaptive amorphous CoO_xCl_y electrocatalyst for sustainable chlorine evolution in acidic brine. *Nat. Commun.* **14**, 5356 (2023).

29. Zhan, Y. et al. Efficient electrocatalytic chlorine evolution of MoO_x modified IrO_2-Ta_2O_5 and its

application in the seawater electrolysis. *J. Electroanal. Chem.* **992**, 119283 (2025).

30. Quan, L. et al. Atomic Pt‐N_4 Sites in Porous N‐Doped Nanocarbons for Enhanced On‐Site Chlorination Coupled with H_2 Evolution in Acidic Water. *Adv. Funct. Mater.* **33**, 2307643 (2023).
31. Menzel, N., Ortel, E., Mette, K., Kraehnert, R.,Strasser, P. Dimensionally Stable Ru/Ir/TiO_2-Anodes with Tailored Mesoporosity for Efficient Electrochemical Chlorine Evolution. *ACS Catal.* **3**, 1324−1333 (2013).
32. Zhu, X. et al. Co_3O_4 nanobelt arrays assembled with ultrathin nanosheets as highly efficient and stable electrocatalysts for the chlorine evolution reaction. *J. Mater. Chem. A* **6**, 12718−12723 (2018).
33. Chen, S., Zheng, Y., Wang, S.,Chen, X. Ti/RuO_2–Sb_2O_5–SnO_2 electrodes for chlorine evolution from seawater. *Chem. Eng. J.* **172**, 47−51 (2011).
34. Hu, M. et al. Ultralow Ru loading RuO_2-TiO_2 with strong oxide-support interaction for efficient chlorine evolution and ammonia-nitrogen-elimination. *Chem. Eng. J.* **465**, 143001 (2023).
35. Ha, H. et al. Highly Selective Active Chlorine Generation Electrocatalyzed by Co_3O_4 Nanoparticles: Mechanistic Investigation through in Situ Electrokinetic and Spectroscopic Analyses. *Phys. Chem. Lett* **10**, 1226−1233 (2019).
36. Jiang, M. et al. Superaerophobic RuO_2‐Based Nanostructured Electrode for High‐Performance Chlorine Evolution Reaction. *Small* **13**, 1602240 (2016).
37. Qiu, L. et al. Europium doped RuO_2@TP enhanced chlorine evolution reaction performance by charge redistribution. *Chem. Eng. J.* **464**, 142623 (2023).
38. Yang, J., Zhu, C.,Wang, D. A Simple Organo‐Electrocatalysis System for the Chlor‐Related Industry. *Angew. Chem., Int. Ed.* **63**, e202406883 (2024).

39. Micari, M. et al. Techno-economic analysis of integrated processes for the treatment and valorisation of neutral coal mine effluents. *J. Cleaner Prod.* **270**, 122472 (2020).
