# Supplementary material for: Bernoulli’s principle-mediated Cl2 electrosynthesis
Source: Nat Commun. 2026 Jan 24;17:1062. doi: 10.1038/s41467-025-66643-6 (PMC12852893; doi:10.1038/s41467-025-66643-6)
Supplement: Supplementary file 2 — Description of Additional Supplementary Files [file 41467_2025_66643_MOESM2_ESM.pdf]

Description of Additional Supplementary Files

Supplementary Data 1. Atomic coordinates of the initial and minimized computational models of Ti-MOF and Ti-MOF(O).

Supplementary Video S1. The process of pressure variation in the chamber due to changes in the carrier gas flow rate was demonstrated through COMSOL.

Supplementary Video S2. Schematic video illustrating the effect of the high-speed flowing carrier gas on the separation of Cl_2.

Supplementary Video S3. The operation status of the independent membrane-free chlor-alkali reaction.
